# Characterizing Conical Intersections in DNA/RNA Nucleobases with Multiconfigurational Wave Functions of Varying Active Space Size

**DOI:** 10.1021/acs.jctc.3c00577

**Published:** 2023-10-26

**Authors:** Juliana Cuéllar-Zuquin, Ana Julieta Pepino, Ignacio Fdez. Galván, Ivan Rivalta, Francesco Aquilante, Marco Garavelli, Roland Lindh, Javier Segarra-Martí

**Affiliations:** † Instituto de Ciencia Molecular, Universitat de Valencia, P.O. Box 22085, ES-46071 Valencia, Spain; ‡ Dipartimento di Chimica Industriale “Toso Montanari”, Università di Bologna, Viale del Risorgimento 4, I-40136 Bologna, Italy; § Department of Chemistry − BMC, Uppsala University, P.O. Box 576, SE-75123 Uppsala, Sweden; ∥ ENSL, CNRS, Laboratoire de Chimie UMR 5182, 46 Allée d’Italie, 69364 Lyon, France; ⊥ Theory and Simulation of Materials (THEOS), and National Centre for Computational Design and Discovery of Novel Materials (MARVEL), École Polytechnique Fédérale de Lausanne, CH-1015 Lausanne, Switzerland

## Abstract

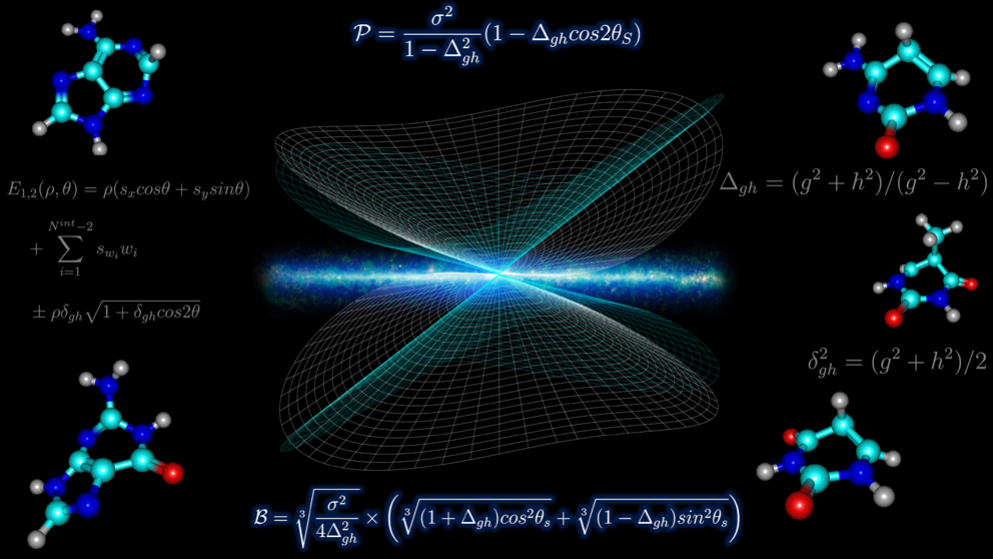

We characterize the photochemically relevant conical intersections between the lowest-lying accessible electronic excited states of the different DNA/RNA nucleobases using Cholesky decomposition-based complete active space self-consistent field (CASSCF) algorithms. We benchmark two different basis set contractions and several active spaces for each nucleobase and conical intersection type, measuring for the first time how active space size affects conical intersection topographies in these systems and the potential implications these may have toward their description of photoinduced phenomena. Our results show that conical intersection topographies are highly sensitive to the electron correlation included in the model: by changing the amount (and type) of correlated orbitals, conical intersection topographies vastly change, and the changes observed do not follow any converging pattern toward the topographies obtained with the largest and most correlated active spaces. Comparison across systems shows analogous topographies for almost all intersections mediating population transfer to the dark ^1^n_O/N_π* states, while no similarities are observed for the “ethylene-like” conical intersection ascribed to mediate the ultrafast decay component to the ground state in all DNA/RNA nucleobases. Basis set size seems to have a minor effect, appearing to be relevant only for purine-based derivatives. We rule out structural changes as a key factor in classifying the different conical intersections, which display almost identical geometries across active space and basis set change, and we highlight instead the importance of correctly describing the electronic states involved at these crossing points. Our work shows that careful active space selection is essential to accurately describe conical intersection topographies and therefore to adequately account for their active role in molecular photochemistry.

## 1 Introduction

DNA/RNA nucleobases are the chromophoric units of our genetic material and have been extensively studied over the years given their prominent role in deleterious photochemical mutations as well as due to their outstanding intrinsic photostability.^1−4^ Their photostability is strongly related to the ability possessed by DNA bases to dissipate the excess energy gained upon absorption in a nonradiative manner at ultrafast time scales, being mediated by a variety of conical intersections (CIs) connecting different excited electronic states with each other and with the ground state, and enabling an efficient funnelling down of excited-state population converting it into thermal energy.^5,6^ This self-protecting mechanism against UV-light damage has been proposed to be key for selecting the nucleobases as the building blocks of our genetic material at prebiotic times under extreme UV-light exposure,^7−10^ by choosing the most suitable (photostable) compounds and thus aiding in its photoprotective design, further securing efficient DNA replication.

The existence of conical intersections is nowadays widely recognized and their importance often highlighted in modern photochemistry,^11−13^ their direct characterization being still elusive by experimental means^14^ even though indirect fingerprints have been recorded.^15^ Given the importance of CIs for rationalizing the outcome of a given photochemical reaction,^16−18^ recent efforts have been put toward efficiently facilitating their characterization in realistic systems. This includes extending the available protocols to hybrid quantum mechanics/molecular mechanics (QM/MM) schemes,^19,20^ hence including solvent effects, or locating CIs with alternative penalty functions^21^ and updated branching plane techniques^22^ that do not require the evaluation of expensive nonadiabatic couplings.

Albeit difficult to describe, CIs have been reported in the literature for a number of years, being detailed first for crossings within states of different symmetry^23,24^ and later on within states of the same symmetry.^25,26^ Different efficient methodologies have been devised over the years for CI optimization: these range from linear-^27^ to quadratic-based^28^ projection techniques to the use of the nudged elastic band method,^29^ and being mostly combined with multiconfigurational techniques that possess first-order nonadiabatic coupling formulations and implementations.^30−34^ Characterizing the potential energy surfaces around conical intersections is important as their shape or topography is believed to influence excited-state reactivity^17,35−38^ and may therefore be fundamental to fully understand photochemistry.

**Figure 1:**
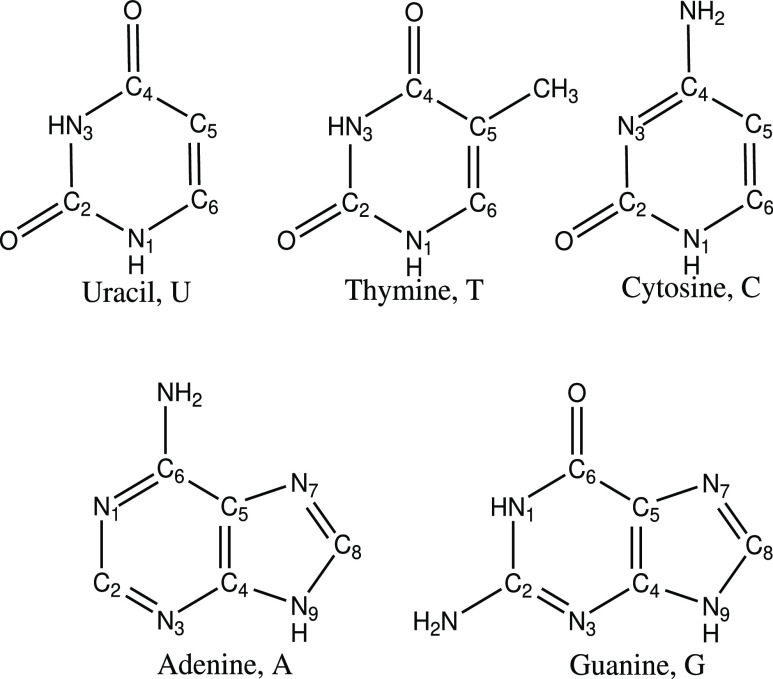
Chemical structure and atom labeling of the DNA and RNA pyrimidine-based (upper half) and purine-based (lower half) nucleobases.

Here, we present a systematic study of the topography of the lowest-lying conical intersections of the different pyrimidine (uracil, thymine, and cytosine) and purine (adenine and guanine) nucleobases (see Figure 1). Despite nucleobase CIs being reported in the literature over the years,^39−48^ this work represents to our knowledge the first systematic analysis of the active space dependence on the characterization of such critical points and provides also an overview of the potential impact shown by the diffuseness of the basis set employed in the optimization procedure.

Active space selection^49^ is a complex process that can potentially affect the outcome of simulations, and our thorough analysis reveals its impact. Using multiple different active spaces for each of the five canonical nucleobases (and totaling over 300 optimized CIs), we find that the different electron correlation included through varying active spaces strongly shapes CI topography: active space strongly affects conical intersection topographies in almost all cases studied. When examining pyrimidines, we observe that the states less affected by changes in the active space were those involving ^1^n_O_π*, with the exception of uracil, which showed significant sensitivity to these changes. On the other hand, conical intersections involving an excited state (regardless of its nature) and the ground state in purines were more influenced by changes in the (static) electron correlation retained in the model. Here, we focus on conical intersection topography due to active space size: other important aspects to excited-state reactivity, such as the accessibility of the different conical intersections characterized, are beyond the scope of the present manuscript.

Interestingly, comparing optimized structures for a given conical intersection reveals minimal changes in the resulting geometry due to active space size. These findings suggest that the topography of conical intersections may depend primarily on the accurate description of the electronic states involved in the crossing rather than on the optimization procedure itself. These results have significant implications for modeling and understanding photoinduced phenomena in DNA and RNA nucleobases.

The manuscript is organized as follows: we first cover the computational details describing the active space selection procedure and other simulation details. The results are separated between pyrimidine and purine derivatives to simplify the analysis, considering the different relevant low-lying (and thus photochemically accessible) CIs featured in the diverse DNA/RNA building blocks. A discussion follows from the similarities that emerge among the different nucleobases, ending with Section 5 that summarizes the present findings.

## 2 Computational Details

All computations were carried out with the OpenMOLCAS package.^50−52^ Different complete active space self-consistent field (CASSCF)^53^ schemes were considered as shown in Figures S1–S5 in the Supporting Information (SI): a systematic procedure was employed whereby active space size was reduced from their full π (occupied and virtual) and n_O/N_ (occupied) valence active space (14 electrons in 10 orbitals (or (14,10) from here onward) for pyrimidines, (16,12) for adenine, and (18,13) for guanine) by removing one by one the least contributing orbitals in terms of occupation number, i.e., those occupied closest to 2 and those unoccupied closest to 0. This leads to slightly different active spaces for the different systems and the different conical intersections studied but that are more consistent within specific systems and CIs. It is worth noting adenine and guanine feature (18,13) and (20,14) full π and n_O/N_ valence active spaces, respectively: in these cases, one n_N_ orbital was removed due to its low contribution and to make computations more feasible (see Figures S4 and S5). Tables S1–S36 in the SI contain the specific orbitals included and information about natural orbital occupation numbers and the natural intersection orbitals^54^ for each of the conical intersections for each of the nucleobases studied. To optimize conical intersections with different active spaces, we used the most correlated (i.e., CASSCF(14,10) for pyrimidines, CASSCF(16,12) for adenine, and CASSCF(18,13) for guanine) as the starting reference. An equal weights state averaging procedure was employed comprising the lowest-lying five roots for pyrimidines and seven roots for purines whenever feasible, with the smallest active spaces being averaged over three roots.

Atomic Natural Orbital basis sets with a large contraction (ANO-L)^55,56^ were used in their double-ζ (VDZP) and triple-ζ (VTZP, available in the SI) polarized contractions. The atomic compact Cholesky decomposition (acCD)^57^ was used throughout to speed-up the two-electron integrals^58−60^ as well as for computing analytic CASSCF gradients^61^ and nonadiabatic couplings.^34^ Conical intersections were characterized using the method introduced by Fdez. Galván et al.^34^ and available in OpenMolcas.^50,51^

All minimum energy conical intersections (referred to as conical intersections from here onward) optimized in this work were analyzed in terms of their  and  parameters, defined in previous work by Fdez. Galván et al.^34^ and that are related to those originally formulated by Ruedenberg and co-workers in their seminal work on conical intersections.^26^ These values are defined as
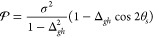
1
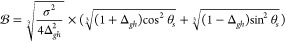
2where σ refers to the pitch, Δ_*gh*_ to the asymmetry, and θ_*s*_ to the tilt heading.^34^ Depending on the value taken by these two parameters ( and ), we have the following classification system
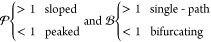


**Figure 2:**
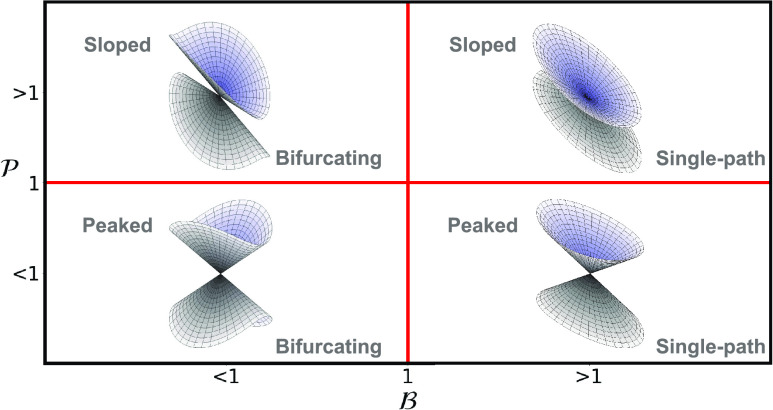
Classification of the conical intersections in terms of  and  parameters with a three-dimensional representation of how are the two potential energy surfaces in the branching space.

A three-dimensional view of the two potential energy surfaces in the branching space for each of the conical intersections resulting from the combination of the  and  parameters described above can be found in Figure 2.

## 3 Results and Discussion

The results are divided in two sections: one for pyrimidine-based (uracil, thymine, and cytosine) and one for purine-based (adenine and guanine) DNA/RNA nucleobases and their (photochemically) most relevant low-energy conical intersections. We start by analyzing the common trends across the different pyrimidine-based systems upon active space change, as they are often grouped together due to their similar photochemistry, and then move onto analyzing how strong correlation affects purine-based systems that display larger reactivity and structural differences. We end by discussing the diverse trends that emerge from comparison across different molecular structures and their resulting conical intersection topographies and attempt to draw some structure–function relationships based on this to compare against the different photochemical behaviors recorded experimentally in the literature for DNA/RNA nucleobases.

### 3.1 Pyrimidines

**Figure 3:**
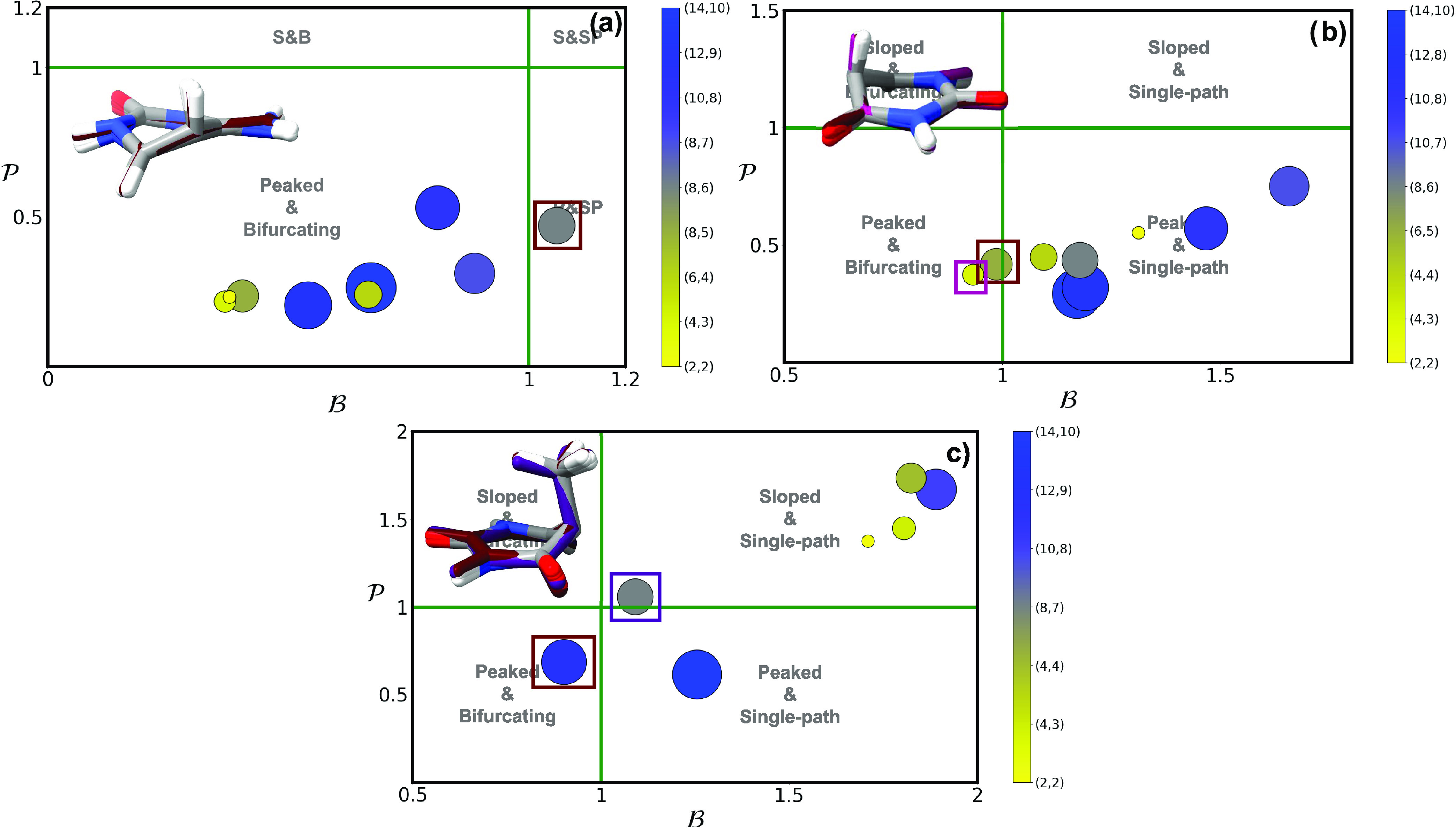
and  parameters of (^1^ππ*/S_0_)_CI_ using multiple different active spaces (see Section 2) for (a) cytosine, (b) uracil and (c) thymine. Active space size is denoted by both marker size and the contour gradient color provided in the right-hand side of each panel. A picture with the superimposed geometries of all optimized conical intersections are provided as insets, with the colored structures representing the outlier intersections marked with a square. An analogous picture with the results obtained using a triple-ζ basis set can be found in the SI.

**Figure 4:**
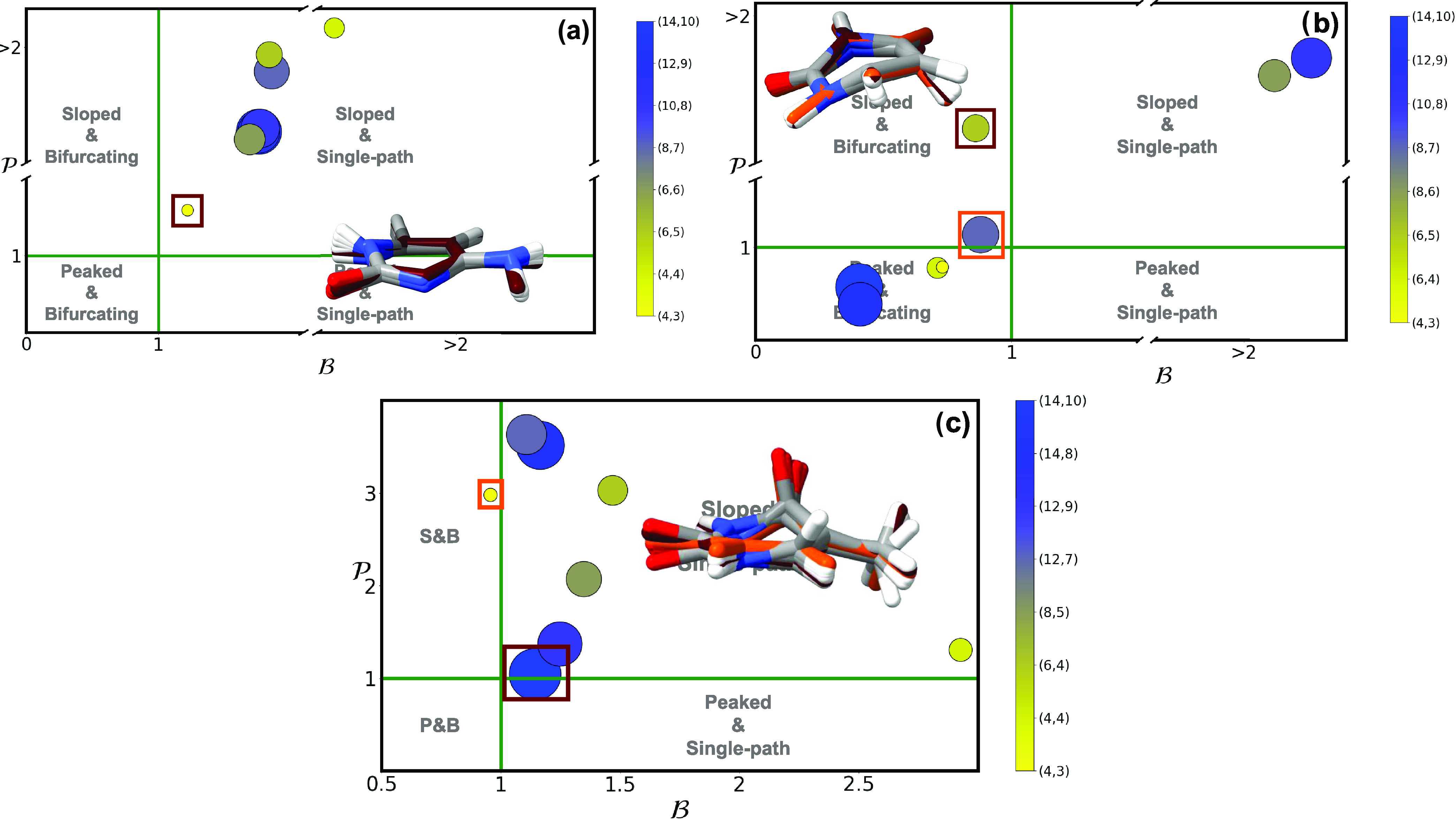
and  parameters of (^1^n_O_π*/^1^ππ*)_CI_ using multiple different active spaces (see Section 2) for (a) cytosine, (b) uracil, and (c) thymine. Active space size is denoted by both marker size and the contour gradient color provided in the right-hand side of each panel. A picture with the superimposed geometries of all optimized conical intersections are provided as insets, with the colored structures representing the outlier intersections marked with a square. An analogous picture with the results obtained using a triple-ζ basis set can be found in the SI.

**Figure 5:**
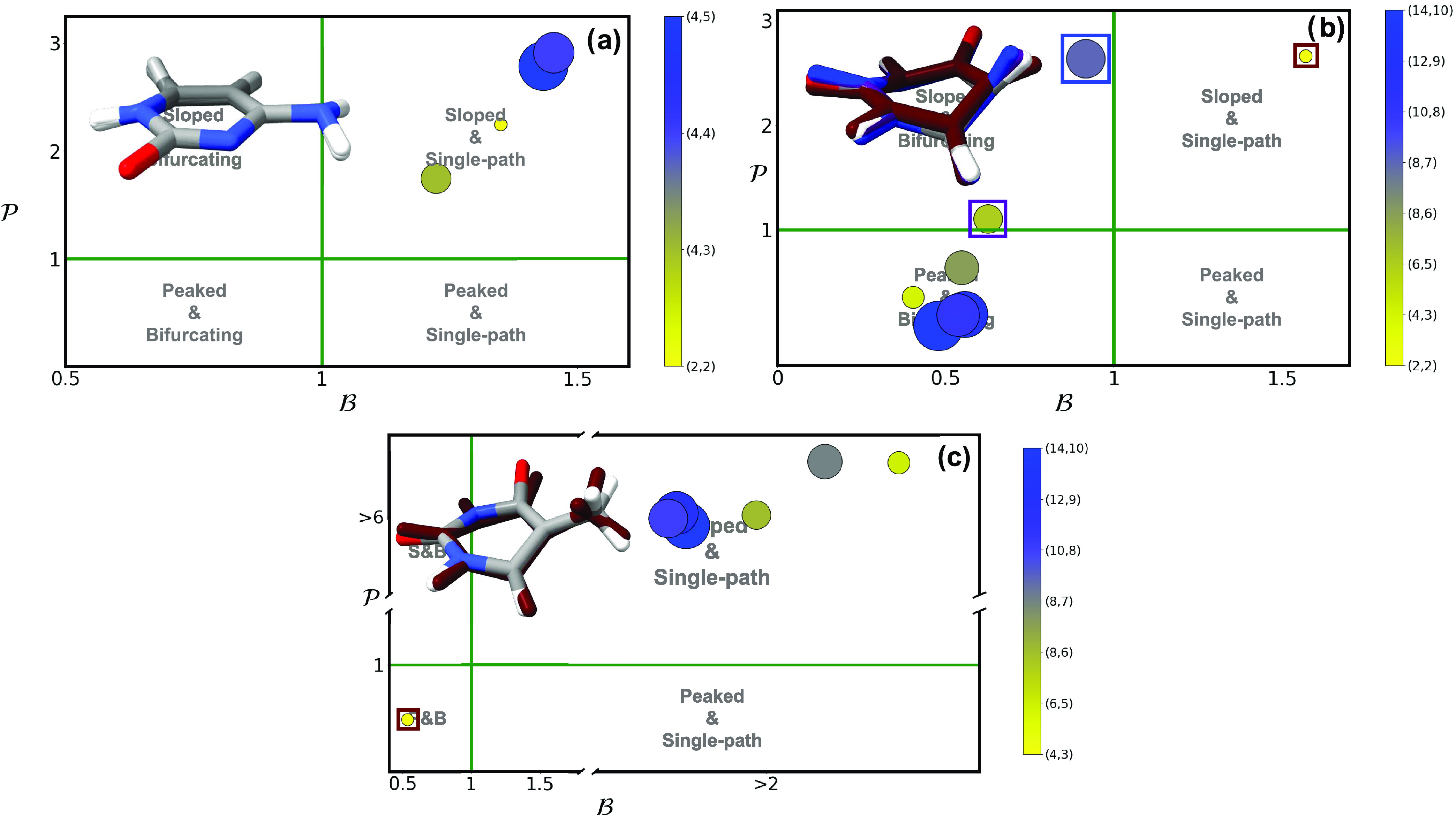
and  parameters of the (^1^n_O_π*/S_0_)_CI_ using multiple different active spaces (see Section 2) for (a) cytosine, (b) uracil, and (c) thymine. Active space size is denoted by both marker size and the contour gradient color provided in the right-hand side of each panel. A picture with the superimposed geometries of all optimized conical intersections are provided as insets, with the colored structures representing the outlier intersections marked with a square. An analogous picture with the results obtained using a triple-ζ basis set can be found in the SI.

**Figure 6:**
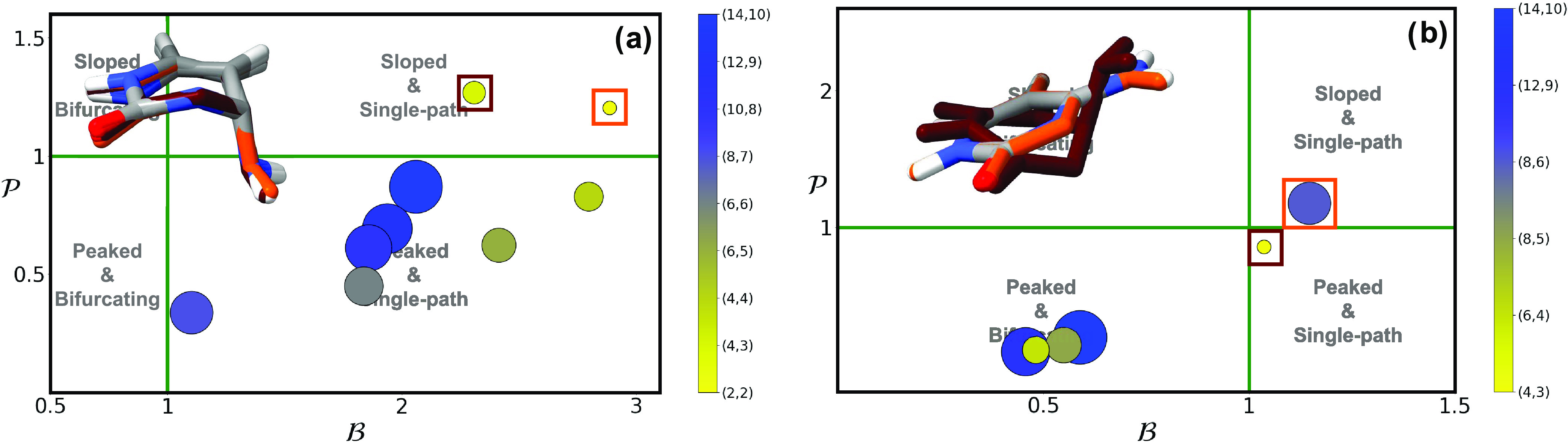
and  parameters of the (a) (^1^n_N_π*/S_0_)_CI_ and (b) (^1^n_N_π*/^1^ππ*)_CI_ of cytosine. Active space size is denoted by both marker size and the contour gradient color provided in the right-hand side of each panel. Pictures with the superimposed geometries of all optimized conical intersections are provided as insets, with the colored structures representing the outlier intersections marked with a square. An analogous picture with the results obtained using a triple-ζ basis set can be found in the SI.

The pyrimidine nucleobases uracil, thymine, and cytosine have a range of low-energy accessible conical intersections, which are depicted in Figures 3–6. These CIs connect the initially accessed ^1^ππ* with neighboring dark states of ^1^n_O_π* (as well as ^1^n_N_π* for cytosine) and the ground state. This leads to (^1^ππ*/S_0_)_CI_, (^1^ππ*/^1^n_O_π*)_CI_, and (^1^n_O_π*/S_0_)_CI_ (as well as (^1^n_N_π*/S_0_)_CI_ and (^1^ππ*/^1^n_N_π*)_CI_ for cytosine). All these different intersections have been invoked (to different degrees)^6,10,45,62^ to rationalize the rich photophysical landscape observed experimentally in DNA pyrimidine-based monomers, their characterization being paramount to fully understand UV-induced DNA photochemistry.

Besides these, it should be mentioned that a number of 3-state conical intersections (i.e., where three different singlet states are degenerate^63^) have also been reported in the literature for these systems, but they are beyond the scope of the present manuscript.^10,40,44,64^

(^1^ππ*/S_0_)_CI_ is perhaps the best known intersection in DNA/RNA pyrimidine nucleobases and entails a pronounced “ethylene-like”^65−67^ twisting leading to an out-of-plane motion in the C5 position^10,39,41−43,45,48,62,68−78^ shared across all pyrimidine-based nucleobases^10,79^ and that is associated with their fastest component of their decay.^68,80^ The main distortions shown by this structure are a pronounced C5–C6 elongation followed by an out-of-plane motion, which are qualitatively shown as insets in Figure 3.

Upon characterizing this intersection with a wide range of active spaces, and thus including varying amounts of electron correlation, we observe different trends for the different pyrimidine nucleobases: cytosine (Figure 3a) shows a consistent peaked and bifurcating character throughout the different active spaces (and basis sets) tested with the exception of (8,6) that appears classed as peaked and single-path with a value of  slightly above 1. Uracil (Figure 3b), on the other hand, favors a peaked and single-path topography with the exception of (6,5) and (4,3) spaces, even if these values approach . Thymine (Figure 3c) displays a much more complex behavior as it features intersections classed in almost every single quadrant (except sloped and bifurcating) depending on the active space used: the largest and presumably most accurate (14,10) space predicts this intersection to be peaked and single-path, while the second most correlated calculation (referred to the (12,9) active space, marked with a brown square in Figure 3c) is classed as peaked and bifurcating but very close to the limits imposed by the classification, as is also (8,7) but bordering in this case from the sloped and single-path quadrant. The remaining optimized structures with other active spaces cluster up at values referred to sloped and single-path topographies.

Significant changes in the (^1^ππ*/S_0_)_CI_ conical intersection topography emerge when varying the correlation included in the model, with no clear trends discernible, which highlights how electron correlation strongly impacts the properties of these important structures. Interestingly, changes are also prominent when considered across pyrimidine nucleobase species: assuming the most correlated (14,10) calculation, which includes all valence π, π*, and lone pair occupied n_O/N_ orbitals, uracil and thymine (Figure 3b,c) show a consistent description of this intersection as peaked and single-path, whereas cytosine (Figure 3a) appears to be peaked and bifurcating.

Despite the vast changes observed in topography, structurally all optimized (^1^ππ*/S_0_)_CI_ conical intersections are very similar. Root-mean-square deviation (RMSD) analyses provided in the SI (Figures S13–S15) show very small values, with deviations below 0.2 Å, which are not sufficient to appropriately discriminate even the outlier cases described above in terms of intersection topography. Interestingly, these small RMS deviations do correlate with noticeable changes in a dihedral angle showcasing out-of-plane motions for each of the pyrimidine nucleobases: H-N_1_-C_4_-O for uracil and thymine and H-N_1_-C_4_-N in cytosine.

We look next at (^1^n_O_π*/^1^ππ*)_CI_ that facilitates population transfer to the optically dark ^1^n_O_π* state and that is present in all pyrimidine nucleobases.^81−85^

In cytosine (Figure 4a), we predict a sloped and single-path character for all different active spaces, with (4,3) approaching borderline values of both  and  but still remaining within the same quadrant as the other calculations. Uracil, on the other hand, displays the largest deviations: the most correlated (14,10) calculation points at a peaked and bifurcating character, while (10,8) and (8,6) display a sloped and single-path topography with values of  and (6,5) and (8,7) classified as sloped and bifurcating with values of  close to 1 in the case of the latter. Thymine (Figure 4c), like cytosine (Figure 4a), shows a sloped and single-path topography with the exception of the (4,3) calculation that has nevertheless values approaching , particularly for its triple-ζ (see SI Figure S7) basis set result.

Looking at the outliers from Figure 4, we can see in this case some clear differences in their structure, particularly for thymine (cf. Figure 4c), where the (4,3) active space puckers much more heavily around the O=C_2_-N_3_-C_4_ = O frame and is clearly discernible from all other optimized structures. Cytosine (Figure 4a) and uracil (Figure 4b) also feature pronounced differences observable by the naked eye in the insets in Figure 4, even if their RMSD analyses are not fully conclusive (see SI Figures S16–S18): we observe how the structures displaying the largest RMSD values (with respect to the most precise (14,10) calculations) correlate with pronounced changes in the H-N_3_-C_6_-H dihedral angle for uracil and thymine and with the H-C_5_-C_2_-O angle for cytosine. These RMS deviations are of the order of ∼0.5 Å and are therefore larger than those observed in the (^1^ππ*/S_0_)_CI_ while being relatively small. They may also be understood as the structures pucker either upward or downward, the varying active spaces leading to either of these analogous (but slightly different) geometries in the gas phase. Optimizations of both upward and downward intersections for each case were attempted, but they always converged to either one of the out-of-plane motions for the different active space studies: we expect this to change when considering nucleobases embedded in complex realistic environments, such as a double-helix DNA structure, where upward and downward (or endo and exo)^86^ conformations result in markedly different energies due to steric hindrance and/or other intermolecular interactions.

The last intersection shared by all pyrimidines is (^1^n_O_π*/S_0_)_CI_, which is shown in Figure 5. Cytosine (Figure 5a) displays a well-defined sloped and single-path topography for all active spaces optimized. In this particular case, we encountered a problem when converging larger active spaces for cytosine: by including more π-type orbitals (both occupied and virtual) optimization leads to (^1^n_O_π*/^1^ππ*)_CI_ instead. It is worth noting, however, that an almost 3-state degeneracy is observed in all cases reported, which is in line with previous work that assigns this to be a 3-state conical intersection.^44^ Uracil (Figure 5b) has a peaked and bifurcating character in most of the conical intersections, with the exceptions of (6,5) and (8,7) classified as sloped and bifurcating and the smaller active space used, (2,2), in the sloped and single-path quadrant. Thymine (Figure 5c), like cytosine, shows a sloped and single-path character for all active spaces studied, with the largest deviation coming from the (4,3) calculation, which is placed in the peaked and bifurcating quadrant.

Similarities arise between (^1^n_O_π*/ππ*)_CI_ and (^1^n_O_π*/S_0_)_CI_: they are both consistent upon active space change for cytosine and thymine but appear to be very sensitive to changes in the active space of uracil. This suggests that there is a significant correlation between active space size and the ability to adequately describe the ^1^n_O_π* excited state in uracil that is not present in cytosine or thymine, despite the latter featuring almost the same molecular structure.

Structurally, (^1^n_O_π*/S_0_)_CI_ features similar differences to those observed above for (^1^n_O_π*/^1^ππ*)_CI_: the intersection triggers a ring puckering that can go either of two ways (upward or downward) depending on the specific active space employed. This leads to larger deviations that are readily observed by the out-of-plane motions at H-N_1_-C_4_-N in cytosine (Figure 5a), H-N_3_-C_6_-H in uracil (Figure 5b), and O-C_2_-C_5_-C in thymine (Figure 5c) and where the changes in these dihedrals correlate with the increases in RMSD observed with respect to the (14,10) reference structures (see Figures S19–S21).

The last two conical intersections, (^1^n_N_π*/S_0_)_CI_ and (^1^n_N_π*/^1^ππ*)_CI_, involve a dark ^1^n_N_π* state and are therefore present only in cytosine.

Figure 6a shows the different estimates obtained for (^1^n_N_π*/S_0_)_CI_, which is classed as peaked and single-path but whose reference (14,10) calculation lies almost at the frontier () of being of sloped character, particularly when using a triple-ζ basis set (see Figure S9). The rest of the calculations remain within the same quadrant with the exception of the smaller (4,3) and (2,2) active spaces, which appear as sloped and single-path by having overestimated values of . (^1^n_N_π*/^1^ππ*)_CI_, presented in Figure 6b, favors a peaked and bifurcating topography for the (14,10) reference calculation. It has two outliers, the (4,3) and (8,6), which lie at the boundaries of the classification within the peaked and sloped single-path quadrants, respectively.

In terms of the structure, (^1^n_N_π*/S_0_)_CI_ features a very pronounced NH_2_ out-of-plane motion (Figure 6a) that is well captured by all active spaces tested, leading to almost identical geometries. This is reminiscent of the result reported above in Figure 3 for the (^1^ππ*/S_0_)_CI_ ethylene-like intersection in all pyrimidine-based systems, where a pronounced out-of-plane motion leads to almost identical structures, which however present largely different CI topographies. The main differences in structure originate from the O-C_2_-C_5_-H dihedral angle, which shows minimal changes in all the cases with RMSD values around ∼0.2 Å (see Figure S22).

(^1^n_N_π*/^1^ππ*)_CI_, on the other hand, features a much more planar structure (Figure 6b) with the exception of the (4,3) outlier, which results in a heavily puckered structure. This puckering is related to the H-*N*_1_-*C*_4_-N dihedral angle mentioned above and depicted in Figure S23 in the SI, which shows a concomitant increase of this dihedral angle with the RMSD computed against the (14,10) reference structure, reaching a value of ∼0.7 Å for the most distorted (4,3) geometry. From this, we may conclude that (^1^nπ*/^1^ππ*)_CI_ intersections do appear to converge to different results in terms of both CI topographies and geometries when computed with minimal active spaces.

Overall, we observe rather pronounced changes in conical intersection topography when varying the active space in DNA/RNA pyrimidine nucleobase monomers: with the exception of those intersections featuring ^1^n_O_π* states in cytosine and thymine, the rest display a vast array of conical intersection topographies that strongly depend on the size of the active space. Structurally, less-pronounced changes are observed, which suggests these differences in topography to be rooted in the ability (or inability) of the different active spaces to appropriately represent the participating electronic states, with the ^1^n_O_π* state in uracil being the most challenging case.

### 3.2 Purines

We turn our attention next to the purine-based canonical nucleobases guanine and adenine. They feature two fused 6- and 5-member cyclic moieties that result in larger molecular frames that correlate more active molecular orbitals and thus leads to more low-lying relevant electronic excited states.

Due to their larger conjugated frame, purine-based nucleobases feature two low-lying ^1^ππ* states that are relevant to the photophysics, often named L_a_(^1^ππ*) and L_b_(^1^ππ*) adapted from Platt’s notation,^87^ as well as a number of lone pair n_N/O_ states. This leads to (L_a_(^1^ππ*)/S_0_)_CI_, (L_a_(^1^ππ*)/L_b_(^1^ππ*))_CI_, and (L_b_(^1^ππ*)/^1^n_N_π*)_CI_ that are common to both bases, as well as (^1^n_N_π*/S_0_)_CI_ and (L_a_(^1^ππ*)/^1^n_N_π*)_CI_ for adenine and (L_a_(^1^ππ*)/^1^n_O_π*)_CI_ and (^1^n_O_π*/S_0_)_CI_ for guanine.

We refrain from including the last four conical intersections mentioned above as they could not be successfully optimized. References in the literature for (^1^n_O_π*/S_0_)_CI_ in guanine^47^ and for (^1^n_N_π*/S_0_)_CI_ in adenine^88^ suggest they feature a pronounced ring-opening component that may not be properly described with the active spaces used here and are therefore considered beyond the scope of the present work. (L_a_(^1^ππ*)/^1^n_N_π*)_CI_ for adenine and (L_a_(^1^ππ*)/^1^n_O_π*)_CI_ for guanine, on the other hand, have been excluded due to most active spaces converging to their respective (L_a_(^1^ππ*)/L_b_(^1^ππ*))_CI_ intersections instead, making their systematic study unfeasible.

The first intersection to analyze is (L_a_(^1^ππ*)/S_0_)_CI_, which is equivalent to the (^1^ππ*/S_0_)_CI_ in pyrimidines, and that is believed to be the main responsible behind the ultrafast decay of purine-based DNA nucleobases.^45−47,88−97^ This crossing is also characterized by an out-of-plane motion that resembles, like in pyrimidine-based nucleobases, the pyramidalized intersection in ethylene.^65^

**Figure 7:**
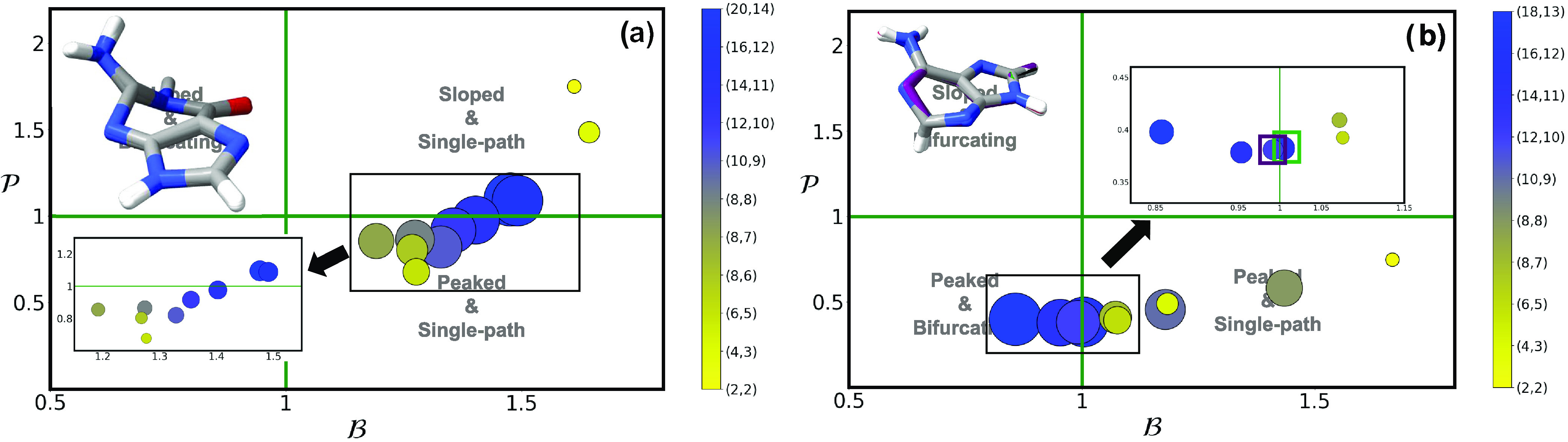
and  parameters of (L_a_(^1^ππ*)/S_0_)_CI_ for (a) guanine and (b) adenine using multiple different active spaces (see Section 2). Active space size is denoted by both marker size and the contour gradient color provided in the right-hand side of each panel. Pictures with the superimposed geometries of all optimized conical intersections are provided as insets, with the colored structures representing the outlier intersections marked with a square. An analogous picture with the results obtained using a triple-ζ basis set can be found in the SI.

Both guanine and adenine present (L_a_(^1^ππ*)/S_0_)_CI_, the different CI topographies along active space change being depicted in panels a and b of Figure 7, respectively.

Figure 7a shows the different intersection topographies obtained with varying active spaces for the (L_a_(^1^ππ*)/S_0_)_CI_ in guanine, most of them clustering up along the dividing line between peaked and sloped quadrants within the single-path character. A zoom-in on this region shows that the more correlated (20,14) and (16,12) active spaces are classed as sloped and single-path, with smaller active spaces being placed within the peaked quadrant, even if they still feature values very close to . We observe that (14,11) displays different topographies for double-ζ and triple-ζ (see Figure S10) basis sets: the values are however very close to the dividing value of , and any differences due to basis set size are therefore expected to be minor.

All (L_a_(^1^ππ*)/S_0_)_CI_ optimized structures in guanine are very similar, as shown by the superimposed geometries displayed as an inset in Figure 7a. Analysis of the RMSD comparing each structure to the most correlated (20,14) active space yields differences in the order of less than 0.1 Å for the largest (and least correlated) cases (see Figure S24), confirming that all optimized structures are essentially equivalent. The small RMS deviations do correlate to an extent with differences registered across geometries in the H-*C*_8_-*C*_2_-N dihedral angle, which decreases concomitantly with the active space size, leading to more planar structures when using a less-correlated (2,2) active space.

The different CI topographies for (L_a_(^1^ππ*)/S_0_)_CI_ across multiple active spaces in adenine are shown in Figure 7b. Similar to guanine, we observe a clustering of the most active space topographies along a dividing line, in this case between peaked single-path and peaked bifurcating quadrants. A zoom-in in this region shows the most accurate (18,13) active space being classed as bifurcating, while most of the rest are in the limit between the two quadrants: interestingly, larger differences are observed due to basis set size in this case, changing from the peaked and bifurcating quadrant with double-ζ to peaked and single-path with triple-ζ (see Figure S10).

Structurally, all geometries are almost identical and display a negligible ∼0.1 Å RMS difference with respect to the most correlated (18,13) geometry (see Figure S25). The very small differences across structures can be mostly associated with changes in the N_1_-*C*_6_–N-H dihedral angle, which approaches −20° for the more correlated (18,13) calculation and ∼5° for the least correlated (2,2). Interestingly, in this case, we observe how the N_1_-*C*_6_–N-H dihedral angle diverges for double-ζ and triple-ζ calculations when considering small active spaces, particularly (2,2), where there is a significant ∼30° difference between them.

**Figure 8:**
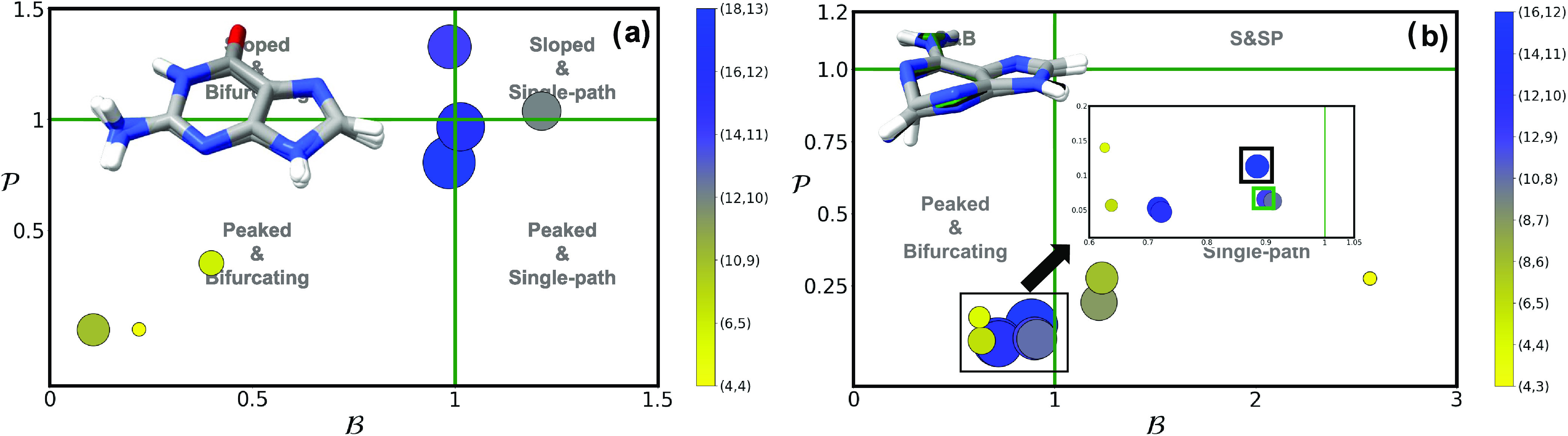
and  parameters of (L_a_(^1^ππ*)/L_b_(^1^ππ*))_CI_ using multiple different active spaces (see Section 2) for guanine (a) and adenine (b). Active space size is denoted by both marker size and the contour gradient color provided in the right-hand side of each panel. Pictures with the superimposed geometries of all optimized conical intersections are provided as insets.

Figure 8 reports results for the (L_a_(^1^ππ*)/L_b_(^1^ππ*))_CI_ conical intersection for both purine nucleobases. In guanine (Figure 8a), the largest active spaces are spread across all four quadrants. The most correlated (18,13) is classified as peaked and bifurcating, while (16,12) is peaked and single-path, the difference being due to a slightly higher value of . We find that (14,11) lies at the boundary between sloped and bifurcating and sloped and single-path, similar to (12,10) with a value of  close to 1. The remaining active spaces all lie within the peaked and bifurcating quadrants, in line with the (18,13) reference. In this particular case, more substantial differences are observed due to increasing basis set size: as shown in SI Figure S11, all active spaces are classified as peaked and bifurcating.

In addition, by looking at the superimposed structures provided as an inset, we observe that they all yield essentially the same geometry. This is further verified by the RMSD values with respect to the most correlated (18,13) calculation, which are less than 0.4 Å in all cases (see Figure S26 in the SI). The most significant change is that occurring in the N_1_-C_2_–N-H dihedral angle, which varies from ∼−80° for (18,13) to ∼−30° for the smallest (4,4) active space.

We observe different trends for adenine (Figure 8b). Most cases are classified as peaked and bifurcating, with (16,12) being placed at the boundary with the peaked and single-path quadrant. (8,6), (8,7), and (4,3) are, on the other hand, classified as peak and single-path. The largest structural differences are found for the H-C_2_-C_8_-H dihedral angle. RMSDs shown in SI Figure S27 are almost zero across active spaces classified as peaked and bifurcating and slightly higher for those with a different classification, (4,3) having the largest value that is in line with its larger  and thus different classification.

**Figure 9:**
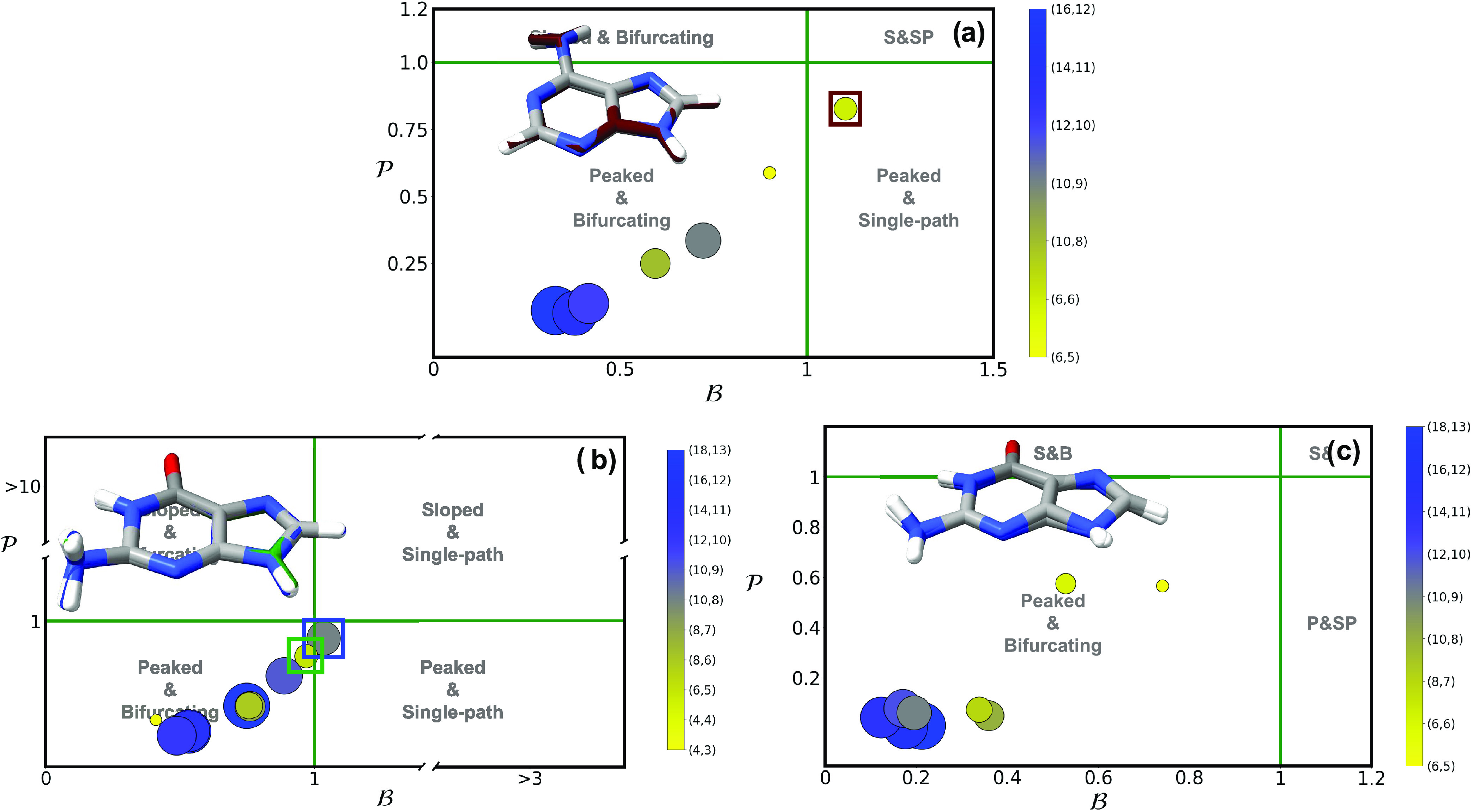
and  parameters of (L_b_(^1^ππ*)/^1^n_N_π*)_CI_ (a) for adenine and (L_a_(^1^ππ*)/^1^n_O_π*)_CI_ (b) and (L_b_(^1^ππ*)/^1^n_N_π*)_CI_ (c) for guanine using multiple different active spaces (see Section 2). Active space size is denoted by both marker size and the contour gradient color provided in the right-hand side of each panel. A picture with the superimposed geometries of all optimized conical intersections is provided as an inset, with the colored structures representing the outlier intersection marked with a purple square.

The last conical intersections studied are depicted in Figure 9. The first one is the (L_b_(^1^ππ*)/^1^n_N_π*)_CI_ of adenine, and it can be observed in subpanel a of Figure 9. This conical intersection appears to be relatively insensitive to (strong) correlation, with all cases classified as peaked and bifurcating except for (6,6), which has a slightly higher value of . This small deviation changes its classification to single-path, but its value of  remains below one, indicating that it is still classified as peaked. RMSD structural analyses and C5-C6–N-H dihedral changes (SI Figure S28) show very small differences, the largest (0.1 Å) being for (6,6) and (6,5) active spaces, which are those appearing at quadrant boundaries in Figure 9a marked by blue and green squares, respectively.

The (L_a_(^1^ππ*)/^1^n_O_π*)_CI_ and (L_b_(^1^ππ*)/^1^n_N_π*)_CI_ of guanine represent the last two conical intersections of interest. In the case of (L_a_(^1^ππ*)/^1^n_O_π*)_CI_ (Figure 9b), almost all cases were classified as peaked and bifurcating, including the most correlated (18,13). The differences between double and triple-ζ (Figure S12) basis sets were the largest observed across all systems and conical intersections. For example, (6,5) appears as peaked and bifurcating with double-ζ and as peaked and single-path with triple-ζ. Likewise, (10,8) is classed as peaked and single-path with double-ζ and sloped and single-path with triple-ζ. These cases fall at the frontier between three quadrants, and thus small changes in  or  can have a significant impact on topography. The RMSDs for this conical intersection are less than 0.2 Å (Figure S29 in the SI), which suggests very small structural changes despite the vast changes in topography noted above.

In the (L_b_(^1^ππ*)/^1^n_N_π*)_CI_ conical intersection of guanine (Figure 9c), all active spaces lead to a peaked and bifurcating topography. At a difference from the previous intersection, small changes arise between double- and triple-ζ (Figure S12) results, which do not affect their classification. Both adenine (Figure 9a) and guanine (Figure 9b) have similar (L_b_(^1^ππ*)/^1^n_N_π*)_CI_ results: with the exception of (6,6) in adenine, all other active spaces appear within the peaked and bifurcating quadrant. This is also observed in the RMSD figures of the SI (Figures S28 and S30), where no correlation could be found between any significant structural changes and the topographies observed.

Summarizing, purine nucleobases also exhibit significant changes in conical intersection topography upon active space change, but to a minor extent to what was observed above for pyrimidines. Conical intersections connecting excited states (regardless of their nature) with the ground state are more influenced by the amount of correlation included in the calculation, with many active spaces at boundary quadrants. The small structural changes observed, similar to those also seen in pyrimidines, again suggest that changes in topography correlate better with the (better or worse) description of the electronic states involved.

## 4 Discussion

A number of conclusions can be drawn upon comparison of the different systems studied.

In structural terms, RMSD analyses show that changes across geometries are very small, ∼0.2 Å for most cases, with 0.6–0.7 Å for the largest deviations registered for (^1^n_O_π*/^1^ππ*)_CI_ in thymine (see Figure S18 in SI) and (^1^n_O_π*/S_0_)_CI_ and (^1^n_N_π*/^1^ππ*)_CI_ in cytosine (see Figures S19 and S23 in the SI). The resulting structures (obtained with different active spaces) are therefore analogous, and this associates the vast changes in conical intersection topography to the varying electron correlation introduced in the different models describing the partaking electronic states by means of changing (increasing) the correlating orbitals.

Another aspect worth highlighting and perhaps expected is the small contribution of basis set size to the calculation outcomes: both double-ζ and triple-ζ (results available in the SI) contractions yield very similar results across all nucleobases and conical intersection types, with some larger deviations being observed for purine-based adenine and guanine. In some particular cases, the topography of conical intersections can be affected by borderline values, leading to different results depending on the basis set used, as was found for example in (L_a_(^1^ππ*)/L_b_(^1^ππ*))_CI_ and (L_a_(^1^ππ*)/^1^n_O_π*)_CI_ for guanine (Figures 8a and 9b respectively), as well as (L_a_(^1^ππ*)/S_0_)_CI_ for both guanine and adenine in Figure 7.

As CASSCF does not add contributions toward electron correlation beyond those orbitals directly included in the active space (i.e., due to strong/static correlation), and due to their localized nature, expanding the basis set has a relatively small effect that is likely more discernible in purine-based species due to their larger molecular frames (and larger amount of correlating orbitals included therein). We expect a (potentially) more prominent role of basis set size when including also dynamic electron correlation,^98^ which we plan to look at in future work.

A brief note should be included regarding active space selection: here we used a systematic approach based on natural orbital occupation numbers (a more in-depth discussion on this based on the natural orbital occupation numbers obtained for each optimized intersection is available in the SI), but this does not necessarily mean that a balanced amount of correlation is included in the model in all cases, particularly when referring to the balanced description of the different electronic states partaking in a given conical intersection. Some of the pronounced deviations observed may therefore arise due to inadequate active space selection, which could be potentially improved by either using automated schemes for active space selection such as those available in the literature^99−103^ or defining variants of these schemes aimed at selecting orbitals at energy degeneracy points, but which are beyond the scope of the present work. This systematic procedure of active space enlargement does aim to progressively increase the amount of static correlation included in the model. It is worth noting, however, that by including more and more molecular orbitals, a degree of dynamic electron correlation is also being retained, particularly for the largest active spaces studied, hence making the changes observed not solely due to static but also partly (even if to a very small degree) due to dynamic electron correlation.

**Figure 10:**
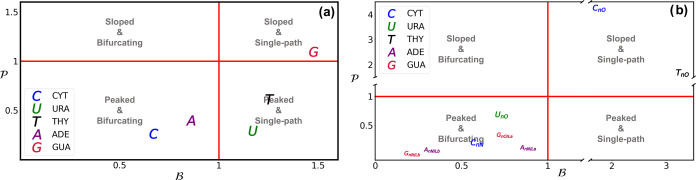
and  parameters of photochemically relevant conical intersections (^1^ππ*/S_0_)_CI_ and (L_a_(^1^ππ*)/S_0_)_CI_ (a) and the different (^1^n_O/N_/^1^ππ*)_CI_ (b) for all DNA/RNA nucleobases, obtained using the largest active space feasible for each system, except for guanine where (18,13) was used in subpanel (b).

Perhaps a more interesting and pertinent comparison within a chemical perspective is that across molecular systems: DNA/RNA nucleobases are expected to display similar excited-state decays following the recorded experimental evidence in the literature.^2,4,6^Figure 10a shows  and  values for (^1^ππ*/S_0_)_CI_ and (L_a_(^1^ππ*)/S_0_)_CI_, which are expected to be the main deactivation funnels responsible for the fastest decay component in DNA/RNA nucleobases.^80^ As can be seen, no clear trends emerge when comparing the different nucleobases while employing their most correlated (and accurate) active spaces tested in this work: uracil and thymine feature a peaked and single-path character and are consistent with one another, as it would be expected from simple methylation; cytosine, on the other hand, showcases a peaked and bifurcating topography; adenine appears to resemble cytosine in displaying a peaked and bifurcating character even when not having the carbonyl group, whereas guanine, despite its structural resemblance to cytosine, shows a sloped and single-path topography.

This suggests that the specific conical intersection topography, or rather the topographies obtained with the most correlated CASSCF simulations used, does not seem to converge to a shared intersection type accounting for a unified or common ultrafast decay mechanism in DNA/RNA nucleobase monomers.^10,79^

Conical intersection topography has been suggested as a potential key shared aspect regulating reactivity: work by Robb and co-workers^104−106^ points at sloped single-path intersections as potential promoters of photostability, as the ground-state gradient in these types of intersections was shown to point toward the regeneration of the reactants. Other authors have also highlighted an active role of conical intersection topography in excited-state decay^13,107^ of a wide range of molecular species,^17,108−111^ being believed to be a crucial component controlling their reactivity. An important aspect arising from this study is defining the smallest amount of static correlation required to qualitatively reproduce the different conical intersection topographies in DNA/RNA nucleobases, the specific active spaces reported in Table 1. The missing dynamic electron correlation can be added to the model in more cost-effective manners (i.e., through perturbation theory as in CASPT2), the size of the active space being the main bottleneck in conical intersection characterization, as well as in relatable simulations like nonadiabatic molecular dynamics. Moreover, working with smaller active spaces allows tackling larger systems, i.e., DNA/RNA dimers and multimers, while still accounting for the essential (competing) monomer-like features observed experimentally for these species.^6^ Our discussion here focuses solely on intersection topographies as we have neglected their accessibility,^18^ i.e., the presence of potential energy barriers, given that CASSCF energies are known to be not quantitative.

**Table 1:** Minimum Active Space Found to Correctly Describe (i.e., in Agreement to the Largest Space Reference) All Different Conical Intersections for Each Nucleobase

	active space
cytosine	(12,9)
uracil	(14,10)
thymine	(14,10)
guanine	(16,12)
adenine	(12,10)

Our CASSCF results (summarized in Table 1) suggest that cytosine requires at least (12,9) to account for all different conical intersections in agreement with their (14,10) reference. For uracil and thymine, we find that the full (14,10) is required as no other space matches the reference for all different conical intersections; we however find that (12,9) reasonably reproduces all but one of the intersections and may be a cost-effective solution. For guanine, we find at least (16,12) to be required to reproduce (L_a_(^1^ππ*)/S_0_)_CI_, which forces the need to use this large active space: this may be due to most active spaces clustering along the  boundary (see Figure 7a). For adenine, we find that a smaller (12,10) suffices to represent all different conical intersections with respect to their (16,12) reference.

We check next whether there are other connections to be made in terms of conical intersection topography beyond those outlined above for (^1^ππ*/S_0_)_CI_, focusing on (^1^n_O/N_/^1^ππ*)_CI_. This intersection controls the population of dark ^1^n_O/N_π* states in DNA/RNA nucleobases and is therefore crucial to understand their overall photochemistry.

In this case, we observe a shared conical intersection topography across most DNA/RNA nucleobase monomers: Figure 10b shows  and  parameters for all n_O/N_π* and ππ* intersections, where we find that almost all intersections display a peaked and bifurcating character that may contribute to the efficient ^1^ππ* → ^1^n_O/N_π* population transfer observed in these species.^112−114^ This includes (^1^n_N_π*/^1^ππ*)_CI_ in cytosine, (^1^n_O_π*/^1^ππ*)_CI_ in uracil, (L_b_(^1^ππ*)/^1^n_N_π*)_CI_ in adenine, and both (L_a_(^1^ππ*)/^1^n_O_π*)_CI_ and (L_b_(^1^ππ*)/^1^n_N_π*)_CI_ in guanine. The only intersection that depicts different topographies when considering the most correlated calculation is (^1^n_O_π*/^1^ππ*)_CI_ for both cytosine and thymine (values of  and  very close to 1), which features a sloped and single-path character.

Our calculations therefore show a more convergent picture across nucleobases for (^1^n_O/N_/^1^ππ*)_CI_ conical intersection topographies than what was found above for (^1^ππ*/S_0_)_CI_. Interestingly, the sole exceptions are cytosine and thymine, which have however been proposed to feature 3-state conical intersections between S_0_, ^1^ππ* and ^1^n_O_π* states^10,64,115^ and that feature distinct (i.e., longer-lived) excited-state dynamics^62,77,80,113−119^ compared to those registered for uracil.^80,120^

On the other hand, it is worth noting that the similarities across the different DNA/RNA monomers for the (^1^n_O/N_/^1^ππ*)_CI_ intersections, as well as the dissimilarities found for the (^1^ππ*/S_0_)_CI_ topographies, may be due to differential correlation effects.^68,69^ These are expected to be less significant when ^1^n_O_π* (strongly covalent) states are involved^121^ and could therefore point at the lack of dynamic electron correlation as the potential missing ingredient to find a shared intersection topography across the different DNA/RNA monomers for their main (^1^ππ*/S_0_)_CI_ ultrafast decay channel in our model.

Interestingly, preliminary calculations with dynamically correlated XMS-CASPT2 surfaces^122−124^ using their largest active space and a double-ζ basis set show converging results across DNA/RNA pyrimidine nucleobases for the “ethylene-like” (^1^ππ*/S_0_)_CI_ topographies leading to a peaked and bifurcating shape. Purine nucleobases (adenine and guanine), on the other hand, converge to a sloped and single-path topography instead for their equivalent (L_a_(^1^ππ*)/S_0_)_CI_. This suggests that pyrimidine and purine DNA/RNA nucleobases have different conical intersection topographies underpinning their ultrafast decay channel, despite featuring similar lifetimes.^125^ A more thorough report will be presented soon covering this and other important aspects related to the effects of dynamic electron correlation at the intersection seam in DNA/RNA species.^98,126^

## 5 Conclusions

In this work, we have, for the first time to our knowledge, analyzed how conical intersection topographies change upon modifying the amount of strong (or static) electron correlation included in the model using CASSCF calculations with varying active space sizes in DNA/RNA canonical nucleobases.

Overall, we observe very large differences in the resulting conical intersection topography due to active space change, which are hard to ascribe to obvious trends in the way strong correlation is included in the model. These large discrepancies, which do not systematically converge upon active space increase, may partly be due to the inadequacy of the conical intersection classification scheme, which appears to be not robust enough to account for the subtle changes introduced due to differences in static correlation. Schemes employing second-^28^ and higher-order descriptions of the intersection seam, resorting to diabatic instead of adiabatic schemes,^127^ or exploring farther regions of the potential energy surfaces may be required to provide an unambiguous classification of the CI topographies in DNA/RNA bases due to changes in active space size.

Interestingly, changing active spaces within the same DNA/RNA nucleobase and intersection type largely results in almost identical optimized geometries with very different topographies. This is the case of the well-known ethylene-like ring-puckering intersection in pyrimidine-based bases, as well as its analogous (L_a_(^1^ππ*)/S_0_)_CI_ in purine-based bases, which produce a wide range of conical intersection topographies while leading to analogous structures. The most correlated level of theory points to different intersection topographies for the distinct DNA/RNA nucleobase monomers, even though they are expected to underpin analogous ultrafast decay channels.

Conical intersections featuring ^1^n_O_π* states, on the other hand, appear to feature more marked structural differences due to out-of-plane motions occurring in two distinct yet analogous directions. More obvious correlations are observed in this case, where despite featuring larger structural differences, we observe a more consistent conical intersection classification across active spaces and where CI topography outliers correlate with the most different structures optimized. Interestingly, intersections between ^1^ππ* and ^1^nπ* states display the same topography across the different nucleobases, with the exception of cytosine and thymine, which are reported to feature 3-state crossings connecting (^1^n_O_/ππ*)_CI_ with S_0_.

While basis set size is found to be negligible in most cases, including all in pyrimidine derivatives, it can have a sizable impact on the topography of conical intersections in purine nucleobases: we find that basis set size can impact intersections at quadrant boundaries, with (L_a_(^1^ππ*)/L_b_(^1^ππ*))_CI_ and (L_a_(^1^ππ*)/^1^n_O_π*)_CI_ in guanine as examples. Additionally, the classification of (L_a_(^1^ππ*)/S_0_)_CI_ for both guanine and adenine is affected by basis set size: this points to larger basis set dependencies with increasing molecular size.

Our findings highlight the vast changes introduced in conical intersection topography upon varying active space size. Given that conical intersections are known to facilitate and even control photochemical reactivity,^13,17,18,108^ our results show that their specific shape may depend very strongly on the amount of static electron correlation included in the model, much more so than previously thought. Ongoing work is analyzing how the inclusion of dynamic electron correlation further affects intersection topographies, aiming to uncover potential biases in their description, which may have undesired effects in the simulation of nonadiabatic events.

## Supporting Information Available

All Cartesian xyz coordinate files for all optimized (minimum energy) conical intersections in this work are readily available through an open-access repository.^128^ Figures and tables with molecular orbitals included in the different active spaces, as well as their labeling, and root-mean-square deviation figures together with a plot displaying the most relevant dihedral angle for each of the different conical intersections (with each active space) and DNA/RNA nucleobases (PDF)
