# Supplementary material for: Characterizing Conical Intersections in DNA/RNA Nucleobases with Multiconfigurational Wave Functions of Varying Active Space Size
Source: J Chem Theory Comput. 2023 Oct 26;19(22):8258–72. doi: 10.1021/acs.jctc.3c00577 (PMC10851440; doi:10.1021/acs.jctc.3c00577)
Supplement: Supplementary file 1 — ct3c00577_si_001.pdf [file ct3c00577_si_001.pdf]

Supporting Information. Characterising conical intersections in DNA/RNA nucleobases with multiconfigurational wave functions of varying active space size

Juliana Cuéllar-Zuquin,[†] Ana Julieta Pepino,[‡] Ignacio Fdez. Galván,[*,¶] Ivan Rivalta,[‡,§] Francesco Aquilante,[‖] Marco Garavelli,[‡] Roland Lindh,[*,¶] and Javier Segarra-Martí[*,†]

†*Instituto de Ciencia Molecular, Universitat de Valencia, P.O. Box 22085, ES-46071 Valencia, Spain*

‡*Dipartimento di Chimica Industriale "Toso Montanari", Università di Bologna, Viale del Risorgimento 4, I-40136 Bologna, Italy*

¶*Department of Chemistry – BMC, Uppsala University, P.O. Box 576, SE-75123 Uppsala, Sweden*

§*ENSL, CNRS, Laboratoire de Chimie UMR 5182, 46 Allée d'Italie, 69364 Lyon France*

‖*Theory and Simulation of Materials (THEOS), and National Centre for Computational Design and Discovery of Novel Materials (MARVEL), École Polytechnique Fédérale de Lausanne, CH-1015 Lausanne, Switzerland*

E-mail: ignacio.fernandez@kemi.uu.se; roland.lindh@kemi.uu.se; javier.segarra@uv.es

Contents

Cartesian Coordinates **3**

Molecular orbitals and active spaces **3**

Root Mean Squared Deviation analyses **36**

Cartesian coordinates

Cartesian coordinates for all optimised minimum energy conical intersections can be accessed through the following DOI/Zenodo repository: 10.5281/zenodo.8348402. In the repository, you will find a zip folder for each conical intersection. Each zip folder contains two subfolders labeled as either DZ or TZ, depending on the basis set used in the optimization, with the cartesian coordinates for each geometry, with the following naming convention:

Nucleobase + **C**onical**I**ntersection + **A**ctive**S**pace + **TZ** (if so)

Molecular orbitals and active spaces

Molecular orbitals (MOs) considered for each nucleobase are shown in figures S1-S5. Based on this information, the different active spaces used in the optimization of each one of the conical intersections and information about Natural Orbital Occupation Numbers (NOONs) can be found in the tables S1-S36 where the notation of the orbitals corresponds to the notation used in the figures. Those tables which give information about NOONs have, for each active space, two different data sets for each of the states involved in the conical intersection.

Cytosine

MOs included in the cytosine calculations with an active space of (14,10) can be seen in Figure S1. For the other cases and for each of the conical intersections, we have different tables (S1,S3,S5,S7,S9) with information on which MOs were included in the calculation as well as other tables with information about how the occupation of those orbitals changes in the different optimizations.

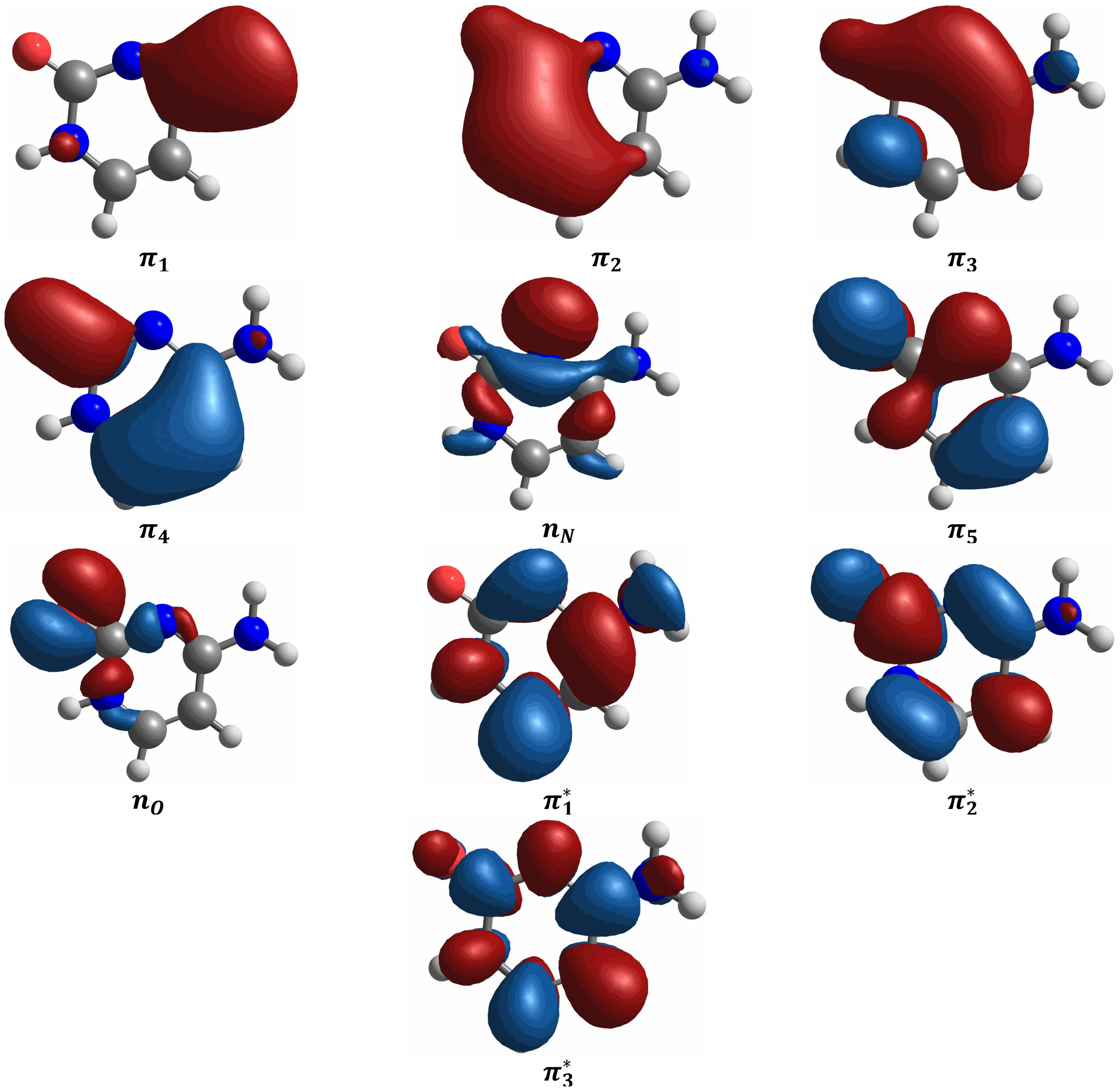

Figure S1: Valence π and $n_{O/N}$ occupied and π unoccupied molecular orbitals of Cytosine, together with their labelling.

Table S1: Molecular orbitals included in the active spaces used for the optimization of the conical intersection $(^1\pi\pi^*/S_0)_{CI}$ for cytosine.

	π_1	π_2	π_3	π_4	n_N	π_5	n_O	π_1^*	π_2^*	π_3^*
(14,10)	✓	✓	✓	✓	✓	✓	✓	✓	✓	✓
(12,9)	X	✓	✓	✓	✓	✓	✓	✓	✓	✓
(10,8)	X	✓	✓	✓	X	✓	✓	✓	✓	✓
(8,7)	X	X	✓	✓	✓	✓	X	✓	✓	✓
(8,6)	X	X	✓	✓	✓	✓	X	✓	✓	X
(8,5)	X	X	✓	X	✓	✓	✓	✓	X	X
(6,4)	X	X	✓	X	✓	✓	X	✓	X	X
(4,3)	X	X	X	X	✓	✓	X	✓	X	X
(2,2)	X	X	X	X	X	✓	X	✓	X	X

Table S2: Occupation numbers for each one of the molecular orbitals involved in the different optimizations of the conical intersection $(^1\pi\pi^*/S_0)_{CI}$ of cytosine. In green are marked those orbitals that participate in the conical intersection under study.

		π_1	π_2	π_3	π_4	nN	π_5	nO	π^*	π^*	π^*
**(2,2)**	State1	X	X	X	X	X	1.950	X	0.050	X	X
	State2	X	X	X	X	X	1.714	X	0.286	X	X
	NIO	X	X	X	X	X	1.500	X	0.500	X	X
**(4,3)**	State1	X	X	X	X	2.000	1.210	X	0.791	X	X
	State2	X	X	X	X	1.999	2.000	X	0.001	X	X
	NIO	X	X	X	X	1.954	1.539	X	0.507	X	X
**(6,4)**	State1	X	X	2.000	X	2.000	1.712	X	0.288	X	X
	State2	X	X	2.000	X	2.000	1.927	X	0.073	X	X
	NIO	X	X	1.942	X	1.977	1.551	X	0.530	X	X
**(8,5)**	State1	X	X	2.000	X	2.000	1.991	2.000	0.010	X	X
	State2	X	X	2.000	X	2.000	1.370	2.000	0.630	X	X
	NIO	X	X	1.976	X	1.899	1.618	1.984	0.524	X	X
**(8,6)**	State1	X	X	1.996	1.988	1.962	1.752	X	0.259	0.044	X
	State2	X	X	1.990	1.999	1.963	1.873	X	0.134	0.042	X
	NIO	X	X	1.964	1.981	1.957	1.490	X	0.521	0.088	X
**(8,7)**	State1	X	X	1.994	1.931	1.955	1.277	X	0.731	0.064	0.050
	State2	X	X	1.935	1.996	1.993	1.993	X	0.008	0.038	0.063
	NIO	X	X	1.952	1.953	1.945	1.492	X	0.500	0.105	0.053
**(10,8)**	State1	X	1.999	1.951	1.997	X	0.908	1.920	1.099	0.075	0.052
	State2	X	1.993	1.931	1.978	X	1.982	1.960	0.074	0.048	0.035
	NIO	X	1.993	1.944	1.976	X	1.472	1.912	0.533	0.117	0.053
**(12,9)**	State1	X	1.999	1.997	1.973	1.995	1.960	1.922	0.078	0.039	0.038
	State2	X	1.999	1.997	1.951	1.994	0.959	1.932	1.049	0.065	0.053
	NIO	X	1.996	1.935	1.975	1.963	1.575	1.841	0.555	0.092	0.066
**(14,10)**	State1	1.999	1.998	1.994	1.924	1.996	1.958	1.974	0.038	0.077	0.043
	State2	1.999	1.997	1.997	1.993	1.951	0.982	1.928	1.028	0.070	0.055
	NIO	1.998	1.993	1.975	1.936	1.963	1.569	1.843	0.555	0.105	0.063

Table S3: Molecular orbitals included in the active spaces used for the optimization of the conical intersection $(^1n_O\pi^*/^1\pi\pi^*)_{CI}$ for cytosine.

	π_1	π_2	π_3	π_4	n_N	π_5	n_O	π_1^*	π_2^*	π_3^*
(14,10)	✓	✓	✓	✓	✓	✓	✓	✓	✓	✓
(12,9)	✓	✓	✓	✓	X	✓	✓	✓	✓	✓
(10,8)	X	✓	✓	✓	X	✓	✓	✓	✓	✓
(8,7)	X	X	✓	✓	X	✓	✓	✓	✓	✓
(6,6)	X	X	✓	X	X	✓	✓	✓	✓	✓
(6,5)	X	X	✓	X	X	✓	✓	✓	✓	X
(4,4)	X	X	X	X	X	✓	✓	✓	✓	X
(4,3)	X	X	X	X	X	✓	✓	✓	X	X

Table S4: Occupation numbers for each one of the molecular orbitals involved in the different optimizations of the conical intersection $(^1n_O\pi^*/^1\pi\pi^*)_{CI}$ of cytosine. In green are marked those orbitals that participate in the conical intersection under study.

		π_1	π_2	π_3	π_4	nN	π_5	nO	π^*	π^*	π^*
**(4,3)**	State1	X	X	X	X	X	2.000	1.091	0.909	X	X
	State2	X	X	X	X	X	1.798	2.000	0.202	X	X
	NIO	X	X	X	X	X	1.514	1.690	0.796	X	X
**(4,4)**	State1	X	X	X	X	X	1.966	1.419	0.580	0.034	X
	State2	X	X	X	X	X	1.249	1.997	0.751	0.003	X
	NIO	X	X	X	X	X	1.590	1.455	0.910	0.045	X
**(6,5)**	State1	X	X	1.902	X	X	1.266	2.000	0.735	0.098	X
	State2	X	X	1.928	X	X	2.000	1.073	0.926	0.074	X
	NIO	X	X	1.901	X	X	1.504	1.516	0.975	0.104	X
**(6,6)**	State1	X	X	1.909	X	X	1.546	1.999	0.452	0.091	0.003
	State2	X	X	1.984	X	X	1.945	1.284	0.717	0.062	0.009
	NIO	X	X	1.915	X	X	1.636	1.496	0.762	0.022	0.169
**(8,7)**	State1	X	X	1.885	1.934	X	1.358	1.999	0.643	0.117	0.063
	State2	X	X	1.916	1.944	X	1.981	1.319	0.687	0.096	0.058
	NIO	X	X	1.891	1.932	X	1.565	1.522	0.899	0.073	0.117
**(10,8)**	State1	X	1.923	1.989	1.948	X	1.878	1.599	0.447	0.158	0.060
	State2	X	1.896	1.995	1.938	X	1.180	1.990	0.827	0.113	0.060
	NIO	X	1.986	1.902	1.926	X	1.642	1.513	0.826	0.075	0.130
**(12,9)**	State1	1.998	1.921	1.989	1.946	X	1.885	1.626	0.416	0.160	0.060
	State2	1.999	1.991	1.995	1.941	X	1.104	1.903	0.903	0.105	0.058
	NIO	1.997	1.986	1.905	1.927	X	1.650	1.510	0.820	0.075	0.130
**(14,10)**	State1	1.995	1.999	1.890	1.998	1.938	1.312	1.994	0.693	0.119	0.063
	State2	1.988	1.998	1.931	1.998	1.955	1.891	1.536	0.501	0.141	0.061
	NIO	1.998	1.997	1.905	1.645	1.927	1.645	1.511	0.826	0.075	0.129

Table S5: Molecular orbitals included in the active spaces used for the optimization of the conical intersection $(^1n_O\pi^*/S_0)_{CI}$ for cytosine.

	π_1	π_2	π_3	π_4	n_N	π_5	n_O	π_1^*	π_2^*	π_3^*
(4,5)	X	X	X	X	X	✓	✓	✓	✓	✓
(4,4)	X	X	X	X	X	✓	✓	✓	✓	X
(4,3)	X	X	X	X	X	✓	✓	✓	X	X
(2,2)	X	X	X	X	X	X	✓	✓	X	X

Table S6: Occupation numbers for each one of the molecular orbtials involved in the different optimizations of the conical intersection $(^1n_O\pi^*/S_0)_{CI}$ of cytosine.

		**π_1**	**π_2**	**π_3**	**π_4**	**nN**	**π_5**	**nO**	**π***	**π***	**π***
**(2,2)**	State1	X	X	X	X	X	X	2.000	0.000	X	X
	State2	X	X	X	X	X	X	1.134	0.867	X	X
	NIO	X	X	X	X	X	X	1.500	0.500	X	X
**(4,3)**	State1	X	X	X	X	X	1.998	1.929	0.074	X	X
	State2	X	X	X	X	X	1.998	1.555	0.447	X	X
	NIO	X	X	X	X	X	1.919	1.500	0.581	X	X
**(4,4)**	State1	X	X	X	X	X	1.979	1.241	0.759	0.021	X
	State2	X	X	X	X	X	1.823	1.981	0.019	0.177	X
	NIO	X	X	X	X	X	1.790	1.500	0.577	0.134	X
**(4,5)**	State1	X	X	X	X	X	1.913	1.552	0.448	0.087	0.000
	State2	X	X	X	X	X	1.900	1.766	0.234	0.101	0.000
	NIO	X	X	X	X	X	1.796	1.499	0.564	0.140	0.001

Table S7: Molecular orbitals included in the active spaces used for the optimization of the conical intersection $(^1n_N\pi^*/S_0)_{CI}$ for cytosine.

	π_1	π_2	π_3	π_4	n_N	π_5	n_O	π_1^*	π_2^*	π_3^*
(14,10)	✓	✓	✓	✓	✓	✓	✓	✓	✓	✓
(12,9)	X	✓	✓	✓	✓	✓	✓	✓	✓	✓
(10,8)	X	X	✓	✓	✓	✓	✓	✓	✓	✓
(8,7)	X	X	X	✓	✓	✓	✓	✓	✓	✓
(6,6)	X	X	X	✓	✓	✓	X	✓	✓	✓
(6,5)	X	X	✓	X	✓	✓	X	✓	✓	X
(4,4)	X	X	X	X	✓	✓	X	✓	✓	X
(4,3)	X	X	X	X	✓	✓	X	✓	X	X
(2,2)	X	X	X	X	✓	X	X	✓	X	X

Table S8: Occupation numbers for each one of the molecular orbtials involved in the different optimizations of the conical intersection $({}^1n_N\pi^*/S_0)_{CI}$ of cytosine.

		π_1	π_2	π_3	π_4	nN	π_5	nO	π^*	π^*	π^*
**(2,2)**	State1	X	X	X	X	1.989	X	X	0.011	X	X
	State2	X	X	X	X	1.395	X	X	0.605	X	X
	NIO	X	X	X	X	1.323	X	X	0.677	X	X
**(4,3)**	State1	X	X	X	X	1.999	1.942	X	0.059	X	X
	State2	X	X	X	X	1.641	2.000	X	0.359	X	X
	NIO	X	X	X	X	1.667	1.789	X	0.544	X	X
**(4,4)**	State1	X	X	X	X	1.761	1.919	X	0.243	0.078	X
	State2	X	X	X	X	1.890	1.939	X	0.107	0.064	X
	NIO	X	X	X	X	1.684	1.728	X	0.500	0.088	X
**(6,5)**	State1	X	X	1.999	X	1.422	1.913	X	0.581	0.086	X
	State2	X	X	1.910	X	1.739	1.995	X	0.267	0.089	X
	NIO	X	X	1.819	X	1.587	1.784	X	0.661	0.149	X
**(6,6)**	State1	X	X	X	1.953	1.843	1.920	X	0.155	0.077	0.051
	State2	X	X	X	1.947	1.797	1.928	X	0.197	0.077	0.055
	NIO	X	X	X	1.941	1.580	1.824	X	0.503	0.090	0.062
**(8,7)**	State1	X	X	X	1.935	1.264	1.912	1.994	0.740	0.091	0.064
	State2	X	X	X	1.985	1.949	1.915	1.982	0.027	0.087	0.055
	NIO	X	X	X	1.964	1.489	1.892	1.945	0.534	0.097	0.078
**(10,8)**	State1	X	X	1.832	1.987	1.984	1.951	1.914	0.187	0.088	0.057
	State2	X	X	1.998	1.994	1.201	1.938	1.914	0.805	0.090	0.061
	NIO	X	X	1.975	1.884	1.490	1.870	1.912	0.681	0.108	0.079
**(12,9)**	State1	X	1.995	1.793	1.980	1.952	1.990	1.916	0.220	0.092	0.062
	State2	X	1.999	1.941	1.996	1.360	1.995	1.919	0.645	0.085	0.058
	NIO	X	1.988	1.980	1.890	1.503	1.880	1.906	0.668	0.108	0.078
**(14,10)**	State1	1.999	1.996	1.998	1.989	1.473	1.941	1.916	0.534	0.092	0.063
	State2	1.996	1.992	1.807	1.950	1.985	1.997	1.922	0.208	0.086	0.057
	NIO	1.986	1.972	1.952	1.895	1.531	1.872	1.949	0.668	0.103	0.072

Table S9: Molecular orbitals included in the active spaces used for the optimization of the conical intersection $({}^1n_N\pi^*/{}^1\pi\pi^*)_{CI}$ for cytosine.

	π_1	π_2	π_3	π_4	n_N	π_5	n_O	π_1^*	π_2^*	π_3^*
(14,10)	✓	✓	✓	✓	✓	✓	✓	✓	✓	✓
(12,9)	X	✓	✓	✓	✓	✓	✓	✓	✓	✓
(8,6)	X	X	✓	X	✓	✓	✓	✓	✓	X
(8,5)	X	X	✓	X	✓	✓	X	✓	X	X
(6,4)	X	X	X	X	✓	✓	✓	✓	X	X
(4,3)	X	X	X	X	✓	✓	X	✓	X	X

Table S10: Occupation numbers for each one of the molecular orbtials involved in the different optimizations of the conical intersection $({}^1n_N\pi^*/{}^1\pi\pi^*)_{CI}$ of cytosine.

		π_1	π_2	π_3	π_4	nN	π_5	nO	π^*	π^*	π^*
**(4,3)**	State1	X	X	X	X	1.995	1.206	X	0.799	X	X
	State2	X	X	X	X	1.284	2.000	X	0.717	X	X
	NIO	X	X	X	X	1.516	1.522	X	0.963	X	X
**(6,4)**	State1	X	X	X	X	1.002	2.000	2.000	0.998	X	X
	State2	X	X	X	X	2.000	1.616	2.000	0.384	X	X
	NIO	X	X	X	X	1.658	1.604	1.842	0.896	X	X
**(8,5)**	State1	X	X	1.999	X	1.000	2.000	1.999	1.002	X	X
	State2	X	X	2.000	X	2.000	1.604	1.984	0.412	X	X
	NIO	X	X	1.863	X	1.644	1.697	1.856	0.941	X	X
**(8,6)**	State1	X	X	1.999	X	1.001	2.000	1.925	0.999	0.077	X
	State2	X	X	2.000	X	2.000	1.526	1.915	0.478	0.082	X
	NIO	X	X	1.925	X	1.575	1.654	1.800	0.938	0.108	X
**(12,9)**	State1	X	1.980	1.925	2.000	1.999	1.442	1.942	0.562	0.094	0.057
	State2	X	1.996	1.994	1.999	1.001	1.924	1.952	1.000	0.084	0.050
	NIO	X	1.984	1.904	1.978	1.521	1.541	1.936	0.949	0.101	0.085
**(14,10)**	State1	1.999	1.996	1.994	1.999	1.000	1.927	1.951	1.000	0.083	0.050
	State2	2.000	1.980	1.929	1.996	1.999	1.432	1.938	0.574	0.094	0.058
	NIO	2.000	1.983	1.944	1.993	1.500	1.506	1.897	0.984	0.110	0.083

The first conical intersection of cytosine is $({}^1\pi\pi^*/S_0)_{CI}$. It be can seen that with larger active spaces, it is observed how one of the electrons in Homo (occ $\sim$2 in the ground state) goes to the Lumo where the NOONs change to $\sim$1 for both molecular orbitals (excited state). As the active space is reduced, the occupation do not change as drastically, giving rise to states in which electronic transfer is not as evident. The other four conical intersections studied for cytosine do not appear to follow the same trend as the previous one. In these cases, the occupation of the orbitals is not very affected by the reduction of the active space (slight changes) however, these differences in the NOONs do not correlate with the results observed in the $\mathcal{P}$ vs $\mathcal{B}$ plots in any case.

Uracil

Similar to the case of cytosine, the orbitals included in the optimizations using an active space of (14,10) are shown in the figure S2. For the rest of the cases, the orbitals can be found in the tables S11-S16.

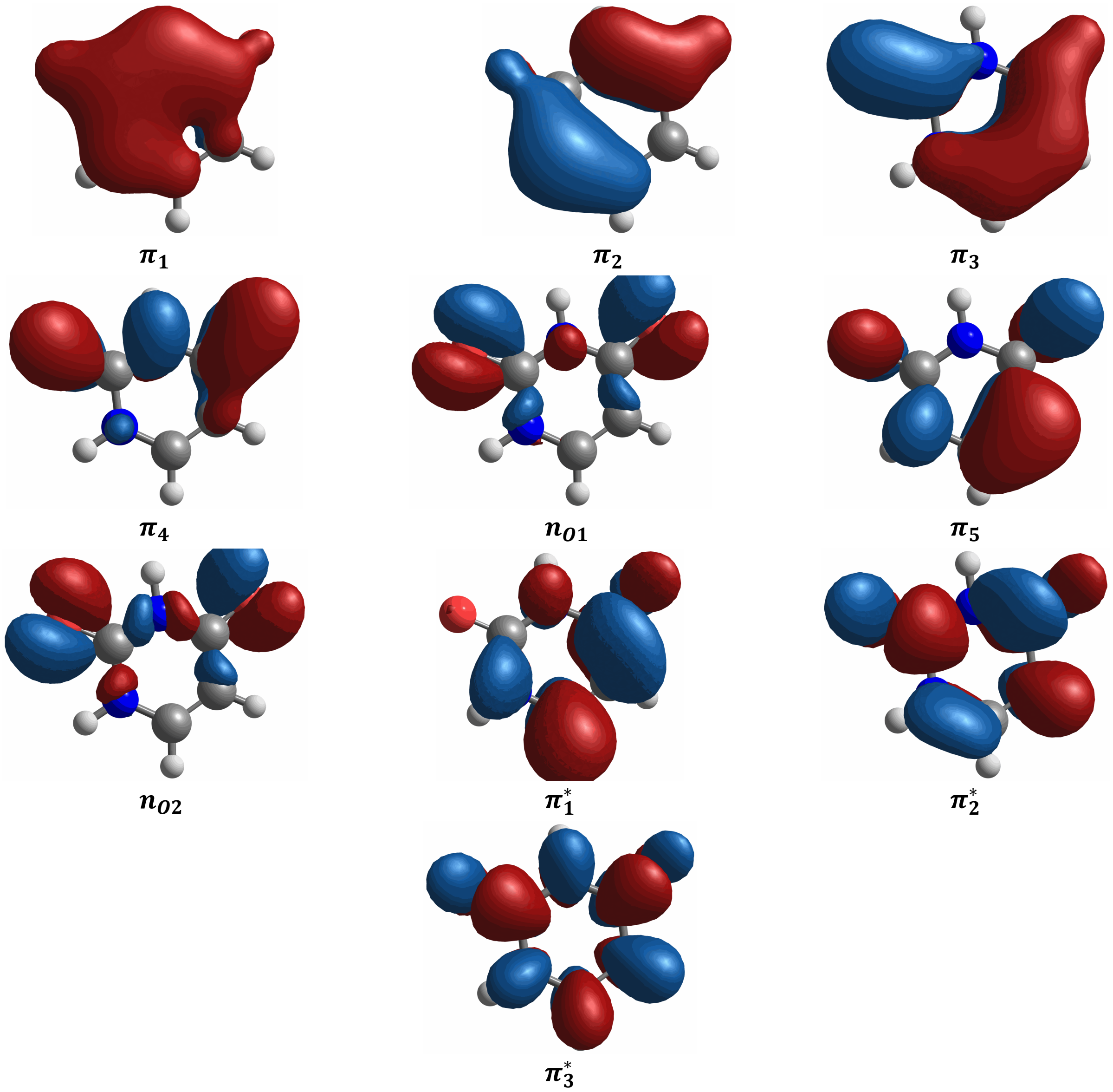

Figure S2: Valence π and n_O occupied and π unoccupied molecular orbitals of Uracil, together with their labelling.

Table S11: Molecular orbitals included in the active spaces used for the optimization of the conical intersection $(^1\pi\pi^*/S_0)_{CI}$ for uracil.

	π_1	π_2	π_3	π_4	n_{O1}	π_5	n_{O2}	π_1^*	π_2^*	π_3^*
(14,10)	✓	✓	✓	✓	✓	✓	✓	✓	✓	✓
(12,8)	✓	X	✓	✓	✓	✓	✓	✓	✓	X
(10,8)	X	X	✓	✓	X	✓	✓	✓	✓	✓
(10,7)	X	X	✓	✓	✓	✓	✓	✓	✓	X
(8,6)	✓	X	✓	✓	X	✓	X	✓	✓	X
(6,5)	X	X	✓	✓	X	✓	X	✓	✓	X
(4,4)	X	X	✓	X	X	✓	X	✓	✓	X
(4,3)	X	X	✓	X	X	✓	X	✓	X	X
(2,2)	X	X	X	X	X	✓	X	✓	X	X

Table S12: Occupation numbers for each one of the molecular orbitals involved in the different optimizations of the conical intersection $(^1\pi\pi^*/S_0)_{CI}$ of uracil. In green are marked those orbitals that participate in the conical intersection under study.

		π_1	π_2	π_3	π_4	nO	π_5	nO	π*	π*	π*
**(2,2)**	**State1**	X	X	X	X	X	2.000	X	0.000	X	X
	**State2**	X	X	X	X	X	1.140	X	0.861	X	X
	**NIO**	X	X	X	X	X	1.500	X	0.500	X	X
**(4,3)**	**State1**	X	X	2.000	X	X	1.116	X	0.884	X	X
	**State2**	X	X	2.000	X	X	1.986	X	0.015	X	X
	**NIO**	X	X	1.756	X	X	1.667	X	0.577	X	X
**(4,4)**	**State1**	X	X	1.993	X	X	1.245	X	0.758	0.004	X
	**State2**	X	X	1.999	X	X	1.978	X	0.023	0.001	X
	**NIO**	X	X	1.957	X	X	1.522	X	0.506	0.014	X
**(6,5)**	**State1**	X	X	1.996	1.946	X	1.975	X	0.030	0.053	X
	**State2**	X	X	1.995	1.960	X	1.380	X	0.622	0.043	X
	**NIO**	X	X	1.958	1.889	X	1.567	X	0.535	0.051	X
**(8,6)**	**State1**	1.972	X	1.998	1.941	X	2.000	X	0.030	0.059	X
	**State2**	1.996	X	1.999	1.955	X	1.082	X	0.920	0.048	X
	**NIO**	1.975	X	1.983	1.909	X	1.546	X	0.528	0.059	X
**(10,7)**	**State1**	X	X	1.996	1.992	2.000	1.582	1.964	0.424	0.042	X
	**State2**	X	X	1.994	1.982	1.999	1.962	1.955	0.065	0.043	X
	**NIO**	X	X	1.986	1.979	1.982	1.504	1.969	0.516	0.062	X
**(10,8)**	**State1**	X	X	1.988	1.953	1.933	1.999	1.994	0.016	0.046	0.072
	**State2**	X	X	1.999	1.957	1.932	1.129	1.999	0.873	0.041	0.070
	**NIO**	X	X	1.985	1.955	1.902	1.502	1.985	0.436	0.042	0.195
**(12,8)**	**State1**	2.000	X	1.944	1.993	1.998	1.483	2.000	0.526	0.057	X
	**State2**	2.000	X	1.937	1.987	1.997	1.972	1.999	0.070	0.038	X
	**NIO**	1.999	X	1.940	1.979	1.976	1.504	1.994	0.495	0.115	X
**(14,10)**	**State1**	1.994	1.974	1.944	2.000	1.935	1.999	1.995	0.031	0.054	0.075
	**State2**	1.995	1.996	1.951	2.000	1.936	1.202	1.999	0.804	0.048	0.069
	**NIO**	1.985	1.973	1.947	1.999	1.939	1.993	1.493	0.485	0.064	0.123

Table S13: Molecular orbitals included in the active spaces used for the optimization of the conical intersection $({}^1n_O\pi^*/{}^1\pi\pi^*)_{CI}$ for uracil.

	π_1	π_2	π_3	π_4	n_{O1}	π_5	n_{O2}	π_1^*	π_2^*	π_3^*
(14,10)	✓	✓	✓	✓	✓	✓	✓	✓	✓	✓
(12,9)	X	✓	✓	✓	✓	✓	✓	✓	✓	✓
(10,8)	X	X	✓	✓	✓	✓	✓	✓	✓	✓
(8,7)	X	X	✓	✓	X	✓	✓	✓	✓	✓
(8,6)	X	X	✓	✓	X	✓	✓	✓	✓	X
(6,5)	X	X	✓	X	X	✓	✓	✓	✓	X
(6,4)	X	X	✓	X	X	✓	✓	✓	X	X
(4,3)	X	X	X	X	X	✓	✓	✓	X	X

Table S14: Occupation numbers for each one of the molecular orbtials involved in the different optimizations of the conical intersection $({}^1n_O\pi^*/{}^1\pi\pi^*)_{CI}$ of uracil. In green are marked those orbitals that participate in the conical intersection under study.

		π_1	π_2	π_3	π_4	nO	π_5	nO	π^*	π^*	π^*
**(4,3)**	State1	X	X	X	X	X	2.000	1.023	0.977	X	X
	State2	X	X	X	X	X	1.865	2.000	0.135	X	X
	NIO	X	X	X	X	X	1.501	1.768	0.731	X	X
**(6,4)**	State1	X	X	2.000	X	X	1.772	1.995	0.233	X	X
	State2	X	X	2.000	X	X	2.000	0.952	1.049	X	X
	NIO	X	X	1.973	X	X	1.509	1.728	0.790	X	X
**(6,5)**	State1	X	X	1.998	X	X	1.925	0.905	1.096	0.077	X
	State2	X	X	1.903	X	X	1.413	1.999	0.588	0.097	X
	NIO	X	X	1.865	X	X	1.507	1.606	0.910	0.112	X
**(8,6)**	State1	X	X	1.983	1.948	X	1.846	1.999	0.148	0.077	X
	State2	X	X	1.999	1.997	X	1.897	0.959	1.041	0.107	X
	NIO	X	X	1.978	1.952	X	1.502	1.536	0.866	0.166	X
**(8,7)**	State1	X	X	1.999	1.950	X	1.931	1.041	0.959	0.071	0.049
	State2	X	X	1.911	1.950	X	1.452	1.998	0.549	0.090	0.049
	NIO	X	X	1.831	1.928	X	1.518	1.659	0.891	0.092	0.083
**(10,8)**	State1	X	X	1.958	1.999	1.940	1.898	1.998	0.092	0.075	0.041
	State2	X	X	1.953	1.998	1.997	1.901	0.991	1.011	0.103	0.046
	NIO	X	X	1.947	1.954	1.996	1.501	1.607	0.782	0.161	0.052
**(12,9)**	State1	X	1.995	1.954	1.998	1.991	1.905	0.830	1.175	0.105	0.048
	State2	X	1.977	1.935	1.999	1.954	1.797	1.988	0.211	0.087	0.052
	NIO	X	1.996	1.940	1.971	1.586	1.500	1.586	0.841	0.155	0.056
**(14,10)**	State1	1.999	1.993	1.994	1.977	1.946	1.895	1.328	0.698	0.117	0.053
	State2	1.998	1.992	1.965	1.978	1.950	1.737	1.930	0.287	0.104	0.059
	NIO	1.990	1.984	1.972	1.956	1.924	1.512	1.536	0.910	0.147	0.070

Table S15: Molecular orbitals included in the active spaces used for the optimization of the conical intersection $(^1n_O\pi^*/S_0)_{CI}$ for uracil.

	π_1	π_2	π_3	π_4	n_{O1}	π_5	n_{O2}	π_1^*	π_2^*	π_3^*
(14,10)	✓	✓	✓	✓	✓	✓	✓	✓	✓	X
(12,9)	✓	✓	✓	✓	X	✓	✓	✓	✓	✓
(10,8)	X	✓	✓	✓	X	✓	✓	✓	✓	✓
(8,7)	X	X	✓	✓	X	✓	✓	✓	✓	✓
(8,6)	X	X	✓	✓	X	✓	✓	✓	✓	X
(6,5)	X	X	X	✓	X	✓	✓	✓	✓	X
(4,3)	X	X	X	X	X	✓	✓	✓	X	X
(2,2)	X	X	X	X	X	X	✓	✓	X	X

Table S16: Occupation numbers for each one of the molecular orbtials involved in the different optimizations of the conical intersection $(^1n_O\pi^*/S_0)_{CI}$ of uracil. In green are marked those orbitals that participate in the conical intersection under study.

		**π_1**	**π_2**	**π_3**	**π_4**	**nO**	**π_5**	**nO**	**π^***	**π^***	**π^***
**(2,2)**	State1	X	X	X	X	X	X	1.867	0.133	X	X
	State2	X	X	X	X	X	X	1.861	0.139	X	X
	NIO	X	X	X	X	X	X	1.500	0.500	X	X
**(4,3)**	State1	X	X	X	X	X	2.000	1.041	0.960	X	X
	State2	X	X	X	X	X	1.865	2.000	0.135	X	X
	NIO	X	X	X	X	X	1.768	1.501	0.731	X	X
**(6,5)**	State1	X	X	X	1.997	X	1.922	1.032	0.967	0.081	X
	State2	X	X	X	1.952	X	1.877	1.999	0.106	0.067	X
	NIO	X	X	X	1.957	X	1.717	1.500	0.682	0.144	X
**(8,6)**	State1	X	X	1.999	1.953	X	2.000	1.094	0.908	0.046	X
	State2	X	X	1.873	1.947	X	1.997	2.000	0.139	0.044	X
	NIO	X	X	1.958	1.865	X	1.793	1.611	0.724	0.049	X
**(8,7)**	State1	X	X	1.998	1.955	X	1.892	1.022	0.978	0.044	0.111
	State2	X	X	1.945	1.957	X	1.907	1.999	0.080	0.037	0.074
	NIO	X	X	1.951	1.951	X	1.663	1.500	0.707	0.046	0.182
**(10,8)**	State1	X	1.991	1.953	1.778	X	1.941	1.982	0.228	0.080	0.048
	State2	X	1.996	1.993	1.925	X	1.956	1.152	0.851	0.083	0.044
	NIO	X	1.974	1.955	1.950	X	1.681	1.500	0.757	0.137	0.047
**(12,9)**	State1	1.995	1.998	1.993	1.929	X	1.954	1.056	0.951	0.079	0.046
	State2	1.995	1.981	1.954	1.768	X	1.937	1.998	0.239	0.077	0.051
	NIO	1.994	1.973	1.957	1.945	X	1.672	1.503	0.771	0.136	0.051
**(14,10)**	State1	1.981	1.996	1.770	1.937	1.995	1.770	1.999	0.240	0.078	0.051
	State2	1.997	1.995	1.955	1.929	1.999	1.955	1.008	0.997	0.079	0.046
	NIO	1.994	1.973	1.956	1.945	1.996	1.670	1.503	0.774	0.139	0.051

As expected, NOONs in Tables S12, S14 and S16 do not explain the differences in the classification of the conical intersections of uracil since the observed changes do not correlate with the $\mathcal{P}$ and $\mathcal{B}$ results.

Thymine

In this case, the orbitals included in the optimizations using an active space of (14,10) are shown in the figure S3. For the rest of the cases, the orbitals can be found in the tables S17-S22.

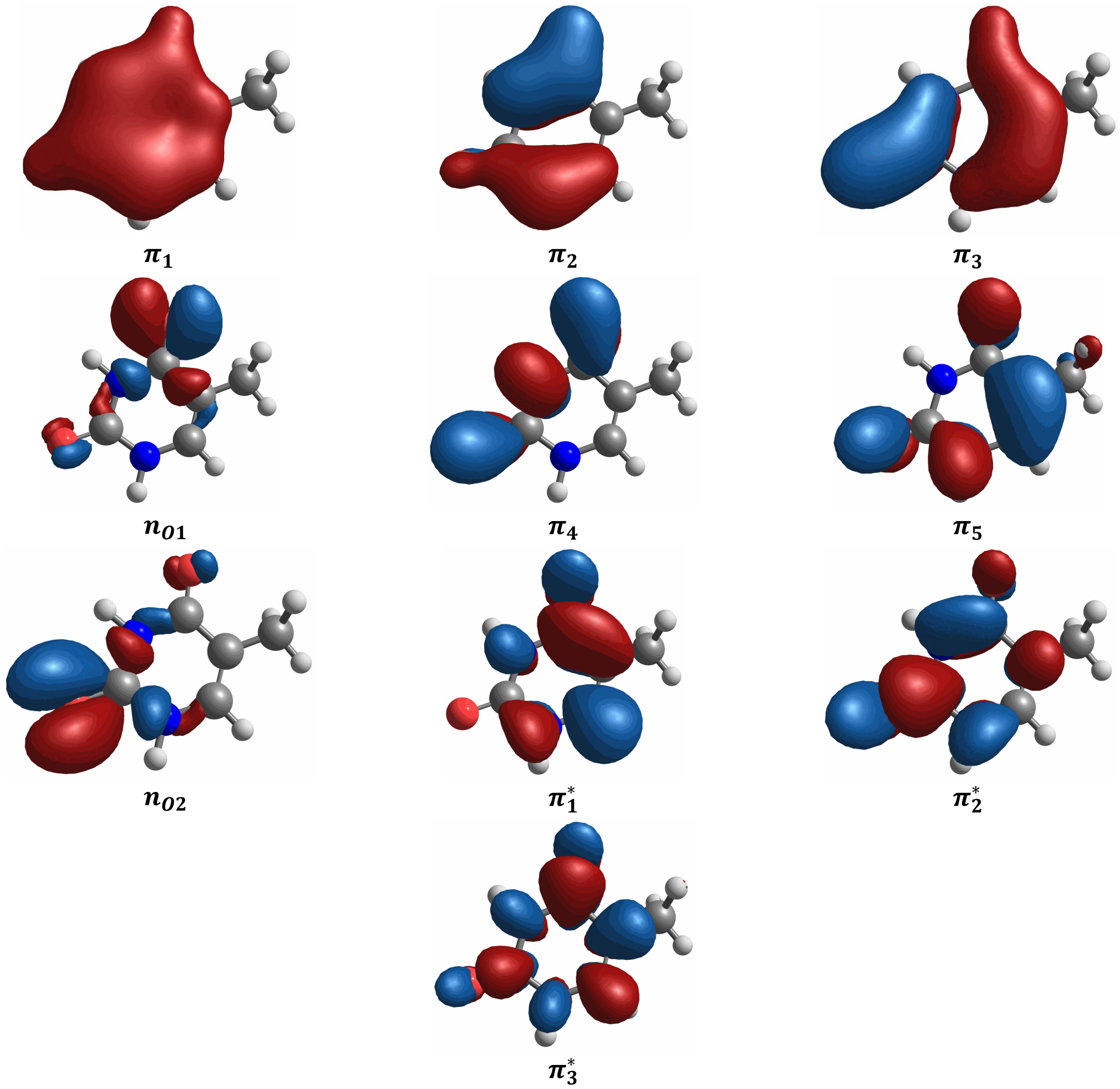

Figure S3: Valence π and n_O occupied and π unoccupied molecular orbitals of Thymine, together with their labelling.

Table S17: Molecular orbitals included in the active spaces used for the optimization of the conical intersection $(^1\pi\pi^*/S_0)_{CI}$ for thymine.

	π_1	π_2	π_3	n_{O1}	π_4	π_5	n_{O2}	π_1^*	π_2^*	π_3^*
(14,10)	✓	✓	✓	✓	✓	✓	✓	✓	✓	✓
(12,9)	X	✓	✓	✓	✓	✓	✓	✓	✓	✓
(10,8)	X	X	✓	X	✓	✓	✓	✓	✓	✓
(8,7)	X	X	✓	X	✓	✓	✓	✓	✓	✓
(4,4)	X	X	X	X	X	✓	✓	✓	✓	X
(4,3)	X	X	X	X	X	✓	✓	✓	X	X
(2,2)	X	X	X	X	X	✓	X	✓	X	X

Table S18: Occupation numbers for each one of the molecular orbitals involved in the different optimizations of the conical intersection $(^1\pi\pi^*/S_0)_{CI}$ of thymine. In green are marked those orbitals that participate in the conical intersection under study.

		π_1	π_2	π_3	nO	π_4	π_5	nO	π^*	π^*	π^*
**(2,2)**	State1	X	X	X	X	X	1.982	X	0.018	X	X
	State2	X	X	X	X	X	1.566	X	0.434	X	X
	NIO	X	X	X	X	X	1.500	X	0.500	X	X
**(4,3)**	State1	X	X	X	X	X	1.818	2.000	0.182	X	X
	State2	X	X	X	X	X	2.000	1.882	0.118	X	X
	NIO	X	X	X	X	X	1.680	1.799	0.522	X	X
**(4,4)**	State1	X	X	X	X	X	1.530	2.000	0.470	0.000	X
	State2	X	X	X	X	X	1.970	1.999	0.031	0.000	X
	NIO	X	X	X	X	X	1.500	1.968	0.532	0.000	X
**(8,7)**	State1	X	X	1.999	X	1.957	1.185	1.942	0.817	0.060	0.041
	State2	X	X	1.998	X	1.953	1.999	1.944	0.002	0.060	0.044
	NIO	X	X	1.990	X	1.955	1.481	1.923	0.353	0.257	0.042
**(10,8)**	State1	X	1.999	1.999	X	1.930	1.194	1.956	0.808	0.072	0.041
	State2	X	1.994	1.999	X	1.934	1.985	1.953	0.018	0.072	0.047
	NIO	X	1.982	1.954	X	1.897	1.563	1.909	0.481	0.171	0.042
**(12,9)**	State1	X	1.981	1.949	1.996	1.934	1.999	1.997	0.025	0.072	0.048
	State2	X	1.999	1.956	1.997	1.933	1.053	1.998	0.950	0.070	0.043
	NIO	X	1.973	1.989	1.990	1.929	1.506	1.953	0.450	0.166	0.044
**(14,10)**	State1	1.998	1.997	1.955	2.000	1.935	0.979	1.999	1.025	0.069	0.045
	State2	1.995	1.977	1.946	1.996	1.933	1.999	1.992	0.032	0.076	0.053
	NIO	1.995	1.974	1.976	1.984	1.738	1.702	1.947	0.548	0.088	0.049

Table S19: Molecular orbitals included in the active spaces used for the optimization of the conical intersection $({}^1n_O\pi^*/{}^1\pi\pi^*)_{CI}$ for thymine.

	π_1	π_2	π_3	π_4	n_{O1}	π_5	n_{O2}	π_1^*	π_2^*	π_3^*
(14,10)	✓	✓	✓	✓	✓	✓	✓	✓	✓	✓
(14,8)	✓	✓	✓	✓	✓	✓	✓	✓	X	X
(12,9)	X	✓	✓	✓	✓	✓	✓	✓	✓	✓
(12,7)	X	✓	✓	✓	✓	✓	✓	✓	X	X
(8,5)	X	X	✓	X	✓	✓	✓	✓	X	X
(6,4)	X	X	✓	X	X	✓	✓	✓	X	X
(4,4)	X	X	X	X	X	✓	✓	✓	✓	X
(4,3)	X	X	X	X	X	✓	✓	✓	X	X

Table S20: Occupation numbers for each one of the molecular orbitals involved in the different optimizations of the conical intersection $({}^1n_O\pi^*/{}^1\pi\pi^*)_{CI}$ of thymine. In green are marked those orbitals that participate in the conical intersection under study.

		π_1	π_2	π_3	π_4	nO	π_5	nO	π^*	π^*	π^*
**(4,3)**	State1	X	X	X	X	X	1.063	1.982	0.955	X	X
	State2	X	X	X	X	X	1.990	1.032	0.978	X	X
	NIO	X	X	X	X	X	1.504	1.494	1.003	X	X
**(4,4)**	State1	X	X	X	X	X	1.051	1.760	0.962	0.227	X
	State2	X	X	X	X	X	1.578	1.191	0.874	0.357	X
	NIO	X	X	X	X	X	1.155	1.483	0.860	0.502	X
**(6,4)**	State1	X	X	1.990	X	X	1.978	0.975	1.058	X	X
	State2	X	X	1.975	X	X	1.148	1.993	0.885	X	X
	NIO	X	X	1.929	X	X	1.498	1.501	1.072	X	X
**(8,5)**	State1	X	X	1.980	X	2.000	0.932	1.992	1.096	X	X
	State2	X	X	1.987	X	1.977	0.971	2.000	1.066	X	X
	NIO	X	X	1.839	X	1.970	1.502	1.653	1.035	X	X
**(12,7)**	State1	X	2.000	2.000	2.000	1.964	1.988	0.878	1.170	X	X
	State2	X	2.000	2.000	2.000	1.979	0.999	1.964	1.059	X	X
	NIO	X	1.998	1.986	1.995	1.922	1.527	1.510	1.063	X	X
**(12,9)**	State1	X	1.987	1.992	1.975	1.936	1.949	0.978	1.062	0.045	0.076
	State2	X	1.987	1.998	1.930	1.988	1.031	1.919	0.999	0.068	0.082
	NIO	X	1.960	1.989	1.961	1.948	1.475	1.471	1.009	0.067	0.121
**(14,8)**	State1	2.000	2.000	2.000	2.000	1.984	2.000	0.821	1.196	X	X
	State2	1.994	1.989	2.000	2.000	2.000	1.003	2.000	1.014	X	X
	NIO	1.998	1.991	1.972	1.998	1.969	1.516	1.499	1.057	X	X
**(14,10)**	State1	1.971	1.958	1.993	1.982	1.996	1.946	1.082	0.954	0.049	0.070
	State2	1.997	1.934	1.998	1.984	1.987	1.048	1.913	0.986	0.064	0.089
	NIO	1.992	1.970	1.964	1.967	1.957	1.487	1.470	0.998	0.063	0.133

Table S21: Molecular orbitals included in the active spaces used for the optimization of the conical intersection $(^1n_O\pi^*/S_0)_{CI}$ for thymine.

	π_1	π_2	π_3	π_4	n_{O1}	π_5	n_{O2}	π_1^*	π_2^*	π_3^*
(14,10)	✓	✓	✓	✓	✓	✓	✓	✓	✓	✓
(12,9)	X	✓	✓	✓	✓	✓	✓	✓	✓	✓
(10,8)	X	X	✓	✓	X	✓	✓	✓	✓	✓
(8,7)	X	X	✓	X	✓	✓	✓	✓	✓	X
(8,6)	X	X	✓	X	✓	✓	✓	✓	✓	X
(6,5)	X	X	✓	X	X	✓	✓	✓	✓	X
(4,3)	X	X	X	X	X	✓	✓	✓	X	X

Table S22: Occupation numbers for each one of the molecular orbitals involved in the different optimizations of the conical intersection $(^1n_O\pi^*/^1\pi\pi^*)_{CI}$ of thymine. In green are marked those orbitals that participate in the conical intersection under study.

		π_1	π_2	π_3	π_4	nO	π_5	nO	π^*	π^*	π^*
**(4,3)**	State1	X	X	X	X	X	1.859	2.000	0.142	X	X
	State2	X	X	X	X	X	2.000	1.288	0.712	X	X
	NIO	X	X	X	X	X	1.778	1.502	0.720	X	X
**(6,5)**	State1	X	X	1.906	X	X	1.411	1.995	0.593	0.095	X
	State2	X	X	1.926	X	X	1.998	0.938	1.064	0.074	X
	NIO	X	X	1.850	X	X	1.608	1.520	0.909	0.113	X
**(8,6)**	State1	X	X	1.906	X	2.000	1.410	1.998	0.592	0.095	X
	State2	X	X	1.995	X	1.998	1.927	0.909	1.096	0.076	X
	NIO	X	X	1.853	X	1.995	1.521	1.611	0.909	0.111	X
**(8,7)**	State1	X	X	1.906	X	1.956	1.410	1.994	0.595	0.095	0.044
	State2	X	X	1.998	X	1.955	1.925	1.083	0.920	0.076	0.044
	NIO	X	X	1.849	X	1.956	1.607	1.525	0.907	0.044	0.114
**(10,8)**	State1	X	X	1.956	1.999	1.411	1.906	1.997	0.591	0.095	0.044
	State2	X	X	1.955	1.997	1.925	1.992	1.120	0.889	0.078	0.044
	NIO	X	X	1.956	1.994	1.856	1.612	1.523	0.903	0.045	0.111
**(12,9)**	State1	X	1.999	1.948	1.996	1.999	1.932	1.020	0.983	0.070	0.054
	State2	X	1.999	1.948	1.996	1.913	1.459	1.998	0.544	0.089	0.054
	NIO	X	1.989	1.864	1.986	1.944	1.633	1.526	0.884	0.079	0.094
**(14,10)**	State1	2.000	1.998	1.955	1.996	1.999	1.417	1.910	0.587	0.093	0.046
	State2	2.000	1.998	1.955	1.992	1.996	1.927	1.133	0.877	0.077	0.046
	NIO	1.997	1.995	1.844	1.984	1.955	1.624	1.55	0.902	0.046	0.103

The findings for the conical intersections of thymine mirror the trends observed for uracil and cytosine, further illustrating that the subtle shifts in NOONs do not provide a satisfactory explanation for the diversities in conical intersection classifications, as the changes in orbital occupancies do not correlate with $\mathcal{P}$ and $\mathcal{B}$ results.

Guanine

As it is well known, the active spaces in the case of purine nucleobases are larger than those of pyrimidine nucleobases. In the specific case of guanine we have used only for the optimization of $(L_a(^1\pi\pi^*)/S_0)_{CI}$ conical intersection, due to the computational cost of the calculations, an active space of 20 electrons distributed in 14 orbitals. These orbitals are shown in Figure S4 while for the rest of the cases in which other active spaces have been used and for each conical intersection we have different tables specifying which MOs are included and how NOONs change.

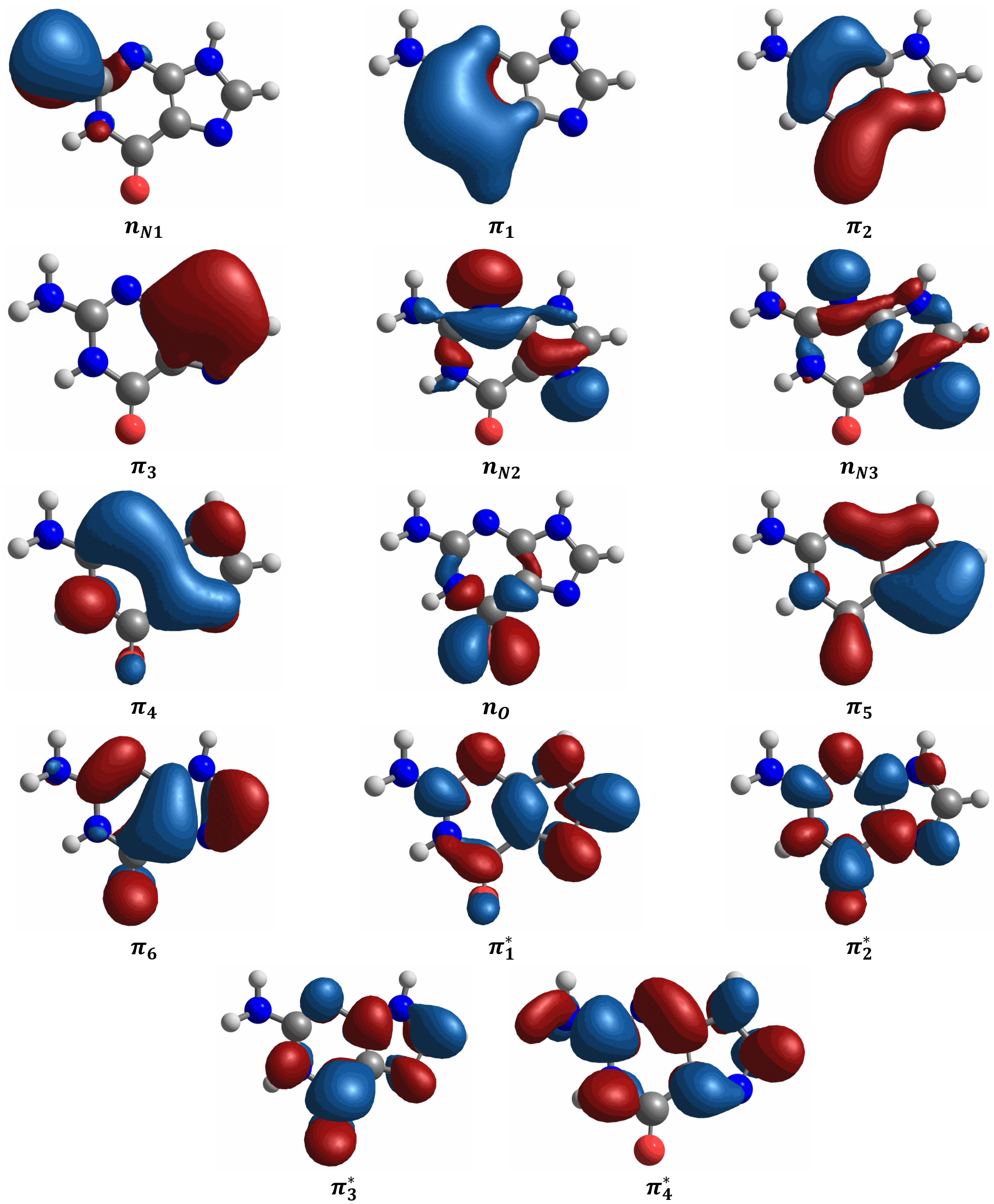

Figure S4: Valence π and $n_{O/N}$ occupied and π unoccupied molecular orbitals of Guanine, together with their labelling. Orbital n_{N1} is removed from almost all active spaces as its occupation number (and therefore its contribution) is negligible.

Table S23: Molecular orbitals included in the active spaces used for the optimization of the conical intersection $(L_a(^1\pi\pi^*)/S_0)_{CI}$ for guanine.

	n_{N1}	π_1	π_2	π_3	n_{N2}	n_{N3}	π_4	n_O	π_5	π_6	π_1^*	π_2^*	π_3^*	π_4^*
(20,14)	✓	✓	✓	✓	✓	✓	✓	✓	✓	✓	✓	✓	✓	✓
(16,12)	X	✓	✓	✓	✓	X	✓	✓	✓	✓	✓	✓	✓	✓
(14,11)	X	✓	✓	✓	X	X	✓	✓	✓	✓	✓	✓	✓	✓
(12,10)	X	✓	✓	X	X	X	✓	✓	✓	✓	✓	✓	✓	✓
(10,9)	X	X	✓	X	X	X	✓	✓	✓	✓	✓	✓	✓	✓
(8,8)	X	X	✓	X	X	X	✓	X	✓	✓	✓	✓	✓	✓
(8,6)	X	X	✓	X	X	X	✓	X	✓	✓	✓	✓	X	X
(6,5)	X	X	X	X	X	✓	✓	X	X	✓	✓	✓	X	X
(4,3)	X	X	X	X	X	✓	X	X	X	✓	✓	X	X	X
(2,2)	X	X	X	X	X	X	X	X	X	✓	✓	X	X	X

Table S24: Occupation numbers for each one of the molecular orbitals involved in the different optimizations of the conical intersection $(L_a(^1\pi\pi^*)/S_0)_{CI}$ of guanine. In green are marked those orbitals that participate in the conical intersection under study.

		nN	π_1	π_2	π_3	nN	nN	π_4	nO	π_5	π_6	π^*	π^*	π^*	π^*
**(2,2)**	State1	X	X	X	X	X	X	X	X	X	1.472	0.528	X	X	X
	State2	X	X	X	X	X	X	X	X	X	1.990	0.010	X	X	X
	NIO	X	X	X	X	X	X	X	X	X	1.500	0.500	X	X	X
**(4,3)**	State1	X	X	X	X	X	1.999	X	X	X	1.602	0.399	X	X	X
	State2	X	X	X	X	X	2.000	X	X	X	1.976	0.024	X	X	X
	NIO	X	X	X	X	X	1.858	X	X	X	1.635	0.507	X	X	X
**(6,5)**	State1	X	X	X	X	X	1.991	1.998	X	X	1.915	0.009	0.087	X	X
	State2	X	X	X	X	X	1.919	1.998	X	X	1.393	0.603	0.087	X	X
	NIO	X	X	X	X	X	1.911	1.997	X	X	1.483	0.502	0.108	X	X
**(8,6)**	State1	X	X	1.927	X	X	X	1.998	X	1.996	1.203	0.797	0.080	X	X
	State2	X	X	2.000	X	X	X	1.999	X	1.997	1.908	0.002	0.094	X	X
	NIO	X	X	1.996	X	X	X	1.947	X	1.955	1.494	0.512	0.097	X	X
**(8,8)**	State1	X	X	1.958	X	X	X	1.932	X	1.944	1.868	0.104	0.082	0.063	0.049
	State2	X	X	1.937	X	X	X	1.918	X	1.943	1.692	0.299	0.089	0.067	0.056
	NIO	X	X	1.932	X	X	X	1.923	X	1.928	1.459	0.535	0.056	0.080	0.087
**(10,9)**	State1	X	X	1.939	X	X	X	1.910	1.972	1.991	1.950	0.036	0.084	0.073	0.045
	State2	X	X	1.996	X	X	X	1.921	1.941	1.917	1.318	0.684	0.089	0.079	0.054
	NIO	X	X	1.942	X	X	X	1.923	1.952	1.945	1.462	0.555	0.052	0.080	0.088
**(12,10)**	State1	X	1.999	1.925	X	X	X	1.932	1.948	1.995	1.728	0.261	0.083	0.074	0.054
	State2	X	1.998	1.941	X	X	X	1.921	1.976	1.951	1.781	0.218	0.089	0.068	0.058
	NIO	X	1.991	1.940	X	X	X	1.925	1.957	1.931	1.453	0.564	0.061	0.091	0.086
**(14,11)**	State1	X	1.914	1.999	1.993	X	X	1.925	1.998	1.940	1.007	0.998	0.077	0.091	0.059
	State2	X	1.967	1.998	1.990	X	X	1.936	1.947	1.982	1.906	0.053	0.087	0.084	0.051
	NIO	X	1.940	1.989	1.989	X	X	1.931	1.957	1.932	1.453	0.569	0.061	0.092	0.087
**(16,12)**	State1	X	1.992	1.943	1.999	1.990	X	1.936	1.969	1.985	1.902	0.084	0.093	0.059	0.050
	State2	X	1.999	1.919	1.997	1.993	X	1.923	1.997	1.940	1.297	0.709	0.090	0.077	0.060
	NIO	X	1.985	1.937	1.978	1.988	X	1.929	1.962	1.953	1.454	0.574	0.062	0.083	0.095
**(20,14)**	State1	1.998	1.928	1.992	1.989	1.999	1.996	1.954	1.998	1.940	1.773	0.220	0.087	0.074	0.053
	State2	1.998	1.940	1.984	1.993	1.999	1.994	1.950	1.998	1.923	1.744	0.257	0.091	0.072	0.059
	NIO	1.995	1.987	1.937	1.977	1.973	1.994	1.922	1.985	1.947	1.459	0.580	0.059	0.086	0.098

Table S25: Molecular orbitals included in the active spaces used for the optimization of the conical intersection $(L_a(^1\pi\pi^*)/L_b(^1\pi\pi^*))_{CI}$ for guanine.

	n_{N1}	π_1	π_2	π_3	n_{N2}	n_{N3}	π_4	n_O	π_5	π_6	π_1^*	π_2^*	π_3^*	π_4^*
(18,13)	X	✓	✓	✓	✓	✓	✓	✓	✓	✓	✓	✓	✓	✓
(16,12)	X	✓	✓	✓	✓	✓	✓	X	✓	✓	✓	✓	✓	✓
(14,11)	X	✓	✓	✓	X	✓	✓	X	✓	✓	✓	✓	✓	✓
(12,10)	X	✓	✓	X	X	✓	✓	X	✓	✓	✓	✓	✓	✓
(10,9)	X	X	✓	X	X	✓	✓	X	✓	✓	✓	✓	✓	✓
(6,5)	X	X	X	X	X	✓	X	X	✓	✓	✓	✓	X	X
(4,4)	X	X	X	X	X	X	X	X	✓	✓	✓	✓	X	X

Table S26: Occupation numbers for each one of the molecular orbitals involved in the different optimizations of the conical intersection $(L_a(^1\pi\pi^*)/L_b(^1\pi\pi^*))_{CI}$ of guanine. In green are marked those orbitals that participate in the conical intersection under study.

		nN	π_1	π_2	π_3	nN	nN	π_4	nO	π_5	π_6	π^*	π^*	π^*	π^*
**(4,4)**	State1	X	X	X	X	X	X	X	X	1.607	1.276	0.665	0.452	X	X
	State2	X	X	X	X	X	X	X	X	1.943	0.643	1.306	0.108	X	X
	NIO	X	X	X	X	X	X	X	X	1.682	1.172	0.793	0.353	X	X
**(6,5)**	State1	X	X	X	X	X	1.981	X	X	1.080	1.817	0.856	0.266	X	X
	State2	X	X	X	X	X	1.985	X	X	1.203	1.854	0.187	0.771	X	X
	NIO	X	X	X	X	X	1.955	X	X	1.788	1.043	0.734	0.479	X	X
**(10,9)**	State1	X	X	1.969	X	X	1.917	1.938	X	1.628	1.058	0.773	0.570	0.097	0.050
	State2	X	X	1.122	X	X	1.943	1.949	X	1.972	1.791	0.935	0.174	0.075	0.041
	NIO	X	X	1.940	X	X	1.927	1.642	X	1.645	1.351	0.866	0.047	0.091	0.491
**(12,10)**	State1	X	1.991	1.941	X	X	1.928	1.997	X	1.818	1.483	0.473	0.228	0.090	0.051
	State2	X	1.995	1.924	X	X	1.907	1.999	X	1.580	1.148	0.844	0.434	0.109	0.061
	NIO	X	1.995	1.923	X	X	1.894	1.989	X	1.696	1.025	0.830	0.385	0.201	0.064
**(14,11)**	State1	X	1.981	1.924	1.962	X	1.892	1.987	X	1.951	1.367	0.609	0.181	0.098	0.050
	State2	X	1.993	1.936	1.977	X	1.931	1.996	X	1.640	1.075	0.865	0.449	0.084	0.054
	NIO	X	1.984	1.966	1.987	X	1.930	1.902	X	1.774	1.029	0.811	0.468	0.094	0.056
**(16,12)**	State1	X	1.930	1.995	1.990	1.999	1.927	1.893	X	1.628	1.120	0.698	0.622	0.130	0.067
	State2	X	1.943	1.986	1.982	1.999	1.920	1.817	X	1.868	1.234	0.291	0.775	0.127	0.057
	NIO	X	1.899	1.983	1.989	1.998	1.925	1.795	X	1.727	1.220	0.182	0.064	0.461	0.756
**(18,13)**	State1	X	1.993	1.930	1.995	1.998	1.994	1.915	1.999	1.585	1.154	0.836	0.438	0.103	0.060
	State2	X	1.981	1.924	1.989	1.998	1.832	1.945	1.999	1.871	1.368	0.307	0.614	0.118	0.055
	NIO	X	1.946	1.925	1.993	1.998	1.985	1.859	1.998	1.500	1.526	0.965	0.181	0.071	0.053

Table S27: Molecular orbitals included in the active spaces used for the optimization of the conical intersection $(L_a(^1\pi\pi^*)/^1n_O\pi^*)_{CI}$ for guanine.

	n_{N1}	π_1	π_2	π_3	n_{N2}	n_{N3}	π_4	n_O	π_5	π_6	π_1^*	π_2^*	π_3^*	π_4^*
(18,13)	X	✓	✓	✓	✓	✓	✓	✓	✓	✓	✓	✓	✓	✓
(16,12)	X	✓	✓	✓	X	✓	✓	✓	✓	✓	✓	✓	✓	✓
(14,11)	X	✓	✓	✓	X	X	✓	✓	✓	✓	✓	✓	✓	✓
(12,10)	X	✓	✓	X	X	X	✓	✓	✓	✓	✓	✓	✓	✓
(10,9)	X	X	✓	X	X	X	✓	✓	✓	✓	✓	✓	✓	✓
(10,8)	X	✓	✓	X	X	X	X	✓	✓	✓	✓	✓	✓	X
(8,7)	X	X	✓	X	X	X	X	✓	✓	✓	✓	✓	✓	X
(8,6)	X	X	✓	X	X	X	X	✓	✓	✓	✓	✓	X	X
(6,5)	X	X	X	X	X	X	X	✓	X	✓	✓	✓	X	X
(4,4)	X	X	X	X	X	X	X	✓	X	✓	✓	✓	X	X
(4,3)	X	X	X	X	X	X	X	✓	X	✓	✓	X	X	X

Table S28: Occupation numbers for each one of the molecular orbitals involved in the different optimizations of the conical intersection $(L_a(^1\pi\pi^*)/^1n_O\pi^*)_{CI}$ of guanine. In green are marked those orbitals that participate in the conical intersection under study.

		nN	π_1	π_2	π_3	nN	nN	π_4	nO	π_5	π_6	π^*	π^*	π^*	π^*
**(4,3)**	State1	X	X	X	X	X	X	X	2.000	X	1.557	0.444	X	X	X
	State2	X	X	X	X	X	X	X	1.073	X	2.000	0.927	X	X	X
	NIO	X	X	X	X	X	X	X	1.503	X	1.583	0.914	X	X	X
**(4,4)**	State1	X	X	X	X	X	X	X	1.118	X	1.991	0.882	0.009	X	X
	State2	X	X	X	X	X	X	X	2.000	X	1.062	0.938	0.001	X	X
	NIO	X	X	X	X	X	X	X	1.504	X	1.473	0.993	0.031	X	X
**(6,5)**	State1	X	X	X	X	X	X	X	1.328	1.964	1.999	0.673	0.036	X	X
	State2	X	X	X	X	X	X	X	1.999	1.963	0.955	1.044	0.040	X	X
	NIO	X	X	X	X	X	X	X	1.498	1.907	1.501	1.016	0.078	X	X
**(8,6)**	State1	X	X	1.981	X	X	X	X	1.302	1.964	1.999	0.704	0.050	X	X
	State2	X	X	1.994	X	X	X	X	1.998	1.957	0.988	1.014	0.050	X	X
	NIO	X	X	1.983	X	X	X	X	1.495	1.911	1.501	1.019	0.092	X	X
**(8,7)**	State1	X	X	1.968	X	X	X	X	1.997	1.914	0.972	1.026	0.085	0.037	X
	State2	X	X	1.967	X	X	X	X	1.355	1.914	2.000	0.645	0.034	0.085	X
	NIO	X	X	1.947	X	X	X	X	1.511	1.873	1.501	1.012	0.095	0.062	X
**(10,8)**	State1	X	1.998	1.961	X	X	X	X	1.907	1.997	1.027	0.973	0.092	0.046	X
	State2	X	1.909	1.966	X	X	X	X	1.389	1.982	1.999	0.614	0.093	0.048	X
	NIO	X	1.981	1.942	X	X	X	X	1.558	1.823	1.500	0.997	0.135	0.062	X
**(10,9)**	State1	X	X	1.905	X	X	X	1.948	1.382	1.941	2.000	0.616	0.096	0.065	0.047
	State2	X	X	1.952	X	X	X	1.998	1.900	1.937	1.009	0.985	0.103	0.067	0.048
	NIO	X	X	1.916	X	X	X	1.941	1.528	1.860	1.500	0.958	0.055	0.067	0.175
**(12,10)**	State1	X	1.994	1.955	X	X	X	1.997	1.900	1.937	1.040	0.956	0.106	0.069	0.047
	State2	X	1.984	1.907	X	X	X	1.954	1.363	1.946	1.999	0.638	0.099	0.064	0.046
	NIO	X	1.985	1.923	X	X	X	1.945	1.530	1.856	1.500	0.965	0.053	0.069	0.175
**(14,11)**	State1	X	1.998	1.994	1.956	X	X	1.994	1.900	1.938	1.025	0.971	0.106	0.070	0.047
	State2	X	1.954	1.984	1.906	X	X	1.992	1.357	1.948	1.999	0.646	0.102	0.066	0.047
	NIO	X	1.947	1.925	1.993	X	X	1.993	1.525	1.8570	1.500	0.965	0.052	0.071	0.181
**(16,12)**	State1	X	1.956	1.939	1.998	X	1.999	1.994	1.900	1.994	1.020	0.976	0.106	0.070	0.047
	State2	X	1.954	1.906	1.948	X	1.999	1.993	1.359	1.984	1.999	0.643	0.102	0.067	0.047
	NIO	X	1.993	1.925	1.947	X	1.998	1.985	1.500	1.856	1.526	0.965	0.052	0.071	0.181
**(18,13)**	State1	X	1.998	1.947	1.954	1.999	1.908	1.992	1.373	1.984	2.000	0.630	0.101	0.067	0.048
	State2	X	1.998	1.938	1.998	1.999	1.957	1.994	1.901	1.994	1.006	0.991	0.106	0.071	0.047
	NIO	X	1.928	1.951	1.998	1.998	1.990	1.984	1.519	1.839	1.500	0.973	0.052	0.074	0.194

Table S29: Molecular orbitals included in the active spaces used for the optimization of the conical intersection $(L_b(^1\pi\pi^*)/^1n_N\pi^*)_{CI}$ for guanine.

	n_{N1}	π_1	π_2	π_3	n_{N2}	n_{N3}	π_4	n_O	π_5	π_6	π_1^*	π_2^*	π_3^*	π_4^*
(18,13)	X	✓	✓	✓	✓	✓	✓	✓	✓	✓	✓	✓	✓	✓
(16,12)	X	✓	✓	✓	✓	✓	✓	X	✓	✓	✓	✓	✓	✓
(14,11)	X	✓	✓	X	✓	✓	✓	X	✓	✓	✓	✓	✓	✓
(12,10)	X	X	✓	X	✓	✓	✓	X	✓	✓	✓	✓	✓	✓
(10,9)	X	X	✓	X	X	✓	✓	X	✓	✓	✓	✓	✓	✓
(10,8)	X	X	✓	X	X	✓	✓	X	✓	✓	✓	✓	✓	X
(8,7)	X	X	X	X	X	✓	✓	X	✓	✓	✓	✓	✓	X
(6,6)	X	X	X	X	X	✓	X	X	✓	✓	✓	✓	✓	X
(6,5)	X	X	X	X	X	✓	X	X	✓	✓	✓	✓	X	X

Table S30: Occupation numbers for each one of the molecular orbitals involved in the different optimizations of the conical intersection $(L_b(^1\pi\pi^*)/^1n_N\pi^*)_{CI}$ of guanine. In green are marked those orbitals that participate in the conical intersection under study.

		nN	π_1	π_2	π_3	nN	nN	π_4	nO	π_5	π_6	π^*	π^*	π^*	π^*
**(6,5)**	State1	X	X	X	X	X	1.537	X	X	1.131	1.985	0.613	0.734	X	X
	State2	X	X	X	X	X	1.993	X	X	0.874	1.847	1.132	0.154	X	X
	NIO	X	X	X	X	X	1.323	X	X	1.742	1.587	0.892	0.456	X	X
**(6,6)**	State1	X	X	X	X	X	1.557	X	X	1.908	1.279	0.853	0.394	0.010	X
	State2	X	X	X	X	X	1.493	X	X	1.944	1.237	0.875	0.442	0.009	X
	NIO	X	X	X	X	X	1.598	X	X	1.753	1.308	0.885	0.445	0.011	X
**(8,7)**	State1	X	X	X	X	X	1.666	1.915	X	1.997	1.031	0.822	0.479	0.090	X
	State2	X	X	X	X	X	1.996	1.946	X	1.030	1.870	0.966	0.133	0.059	X
	NIO	X	X	X	X	X	1.769	1.925	X	1.503	1.369	0.839	0.515	0.080	X
**(10,8)**	State1	X	X	1.996	X	X	1.950	1.927	X	1.237	1.593	0.928	0.300	0.069	X
	State2	X	X	1.995	X	X	1.562	1.919	X	1.940	1.175	0.781	0.547	0.082	X
	NIO	X	X	1.993	X	X	1.772	1.926	X	1.508	1.381	0.817	0.524	0.079	X
**(10,9)**	State1	X	X	1.995	X	X	1.952	1.947	X	1.032	1.873	0.965	0.127	0.070	0.040
	State2	X	X	1.940	X	X	1.688	1.919	X	1.996	1.027	0.812	0.475	0.093	0.050
	NIO	X	X	1.944	X	X	1.770	1.930	X	1.504	1.375	0.810	0.047	0.085	0.535
**(12,10)**	State1	X	X	1.981	X	1.986	1.946	1.951	X	1.063	1.866	0.949	0.145	0.073	0.041
	State2	X	X	1.999	X	1.939	1.716	1.920	X	1.997	1.045	0.436	0.805	0.093	0.051
	NIO	X	X	1.945	X	1.979	1.789	1.930	X	1.527	1.355	0.807	0.047	0.085	0.536
**(14,11)**	State1	X	1.992	1.982	X	1.980	1.949	1.948	X	1.059	1.844	0.969	0.158	0.077	0.042
	State2	X	1.986	1.999	X	1.931	1.808	1.920	X	1.977	1.153	0.327	0.756	0.090	0.053
	NIO	X	1.986	1.939	X	1.979	1.841	1.935	X	1.535	1.356	0.763	0.049	0.085	0.532
**(16,12)**	State1	X	1.949	1.993	1.979	1.984	1.951	1.995	X	1.057	1.877	0.960	0.135	0.077	0.041
	State2	X	1.935	1.980	1.999	1.915	1.834	1.994	X	1.997	1.237	0.247	0.720	0.091	0.051
	NIO	X	1.985	1.936	1.992	1.970	1.853	1.941	X	1.538	1.379	0.746	0.049	0.086	0.519
**(18,13)**	State1	X	1.997	1.948	1.993	1.971	1.950	1.979	1.995	1.064	1.867	0.963	0.152	0.079	0.043
	State2	X	1.999	1.914	1.980	1.999	1.845	1.936	1.997	1.993	1.273	0.233	0.691	0.090	0.051
	NIO	X	1.985	1.936	1.992	1.970	1.860	1.938	1.998	1.546	1.374	0.765	0.049	0.087	0.498

Despite the somewhat more complex character of some of the conical intersections of purines compared to those of pyrimidines (involving more orbitals), the results obtained are similar when dealing with NOONs and the trends observed, in this case, for guanine are analogous to the previous ones. It is not possible to extract decisive data to help explain the

different classifications within the same conical intersection, nor do they give information on how similar are those in the same quadrants.

Adenine

For adenine, the larger active space used was 18 electrons distributed in 13 orbitals as can be seen in Figure S5. Following the same system as before, we have a table specifying which MOs are included in the different calculations and other tables with information about NOONs.

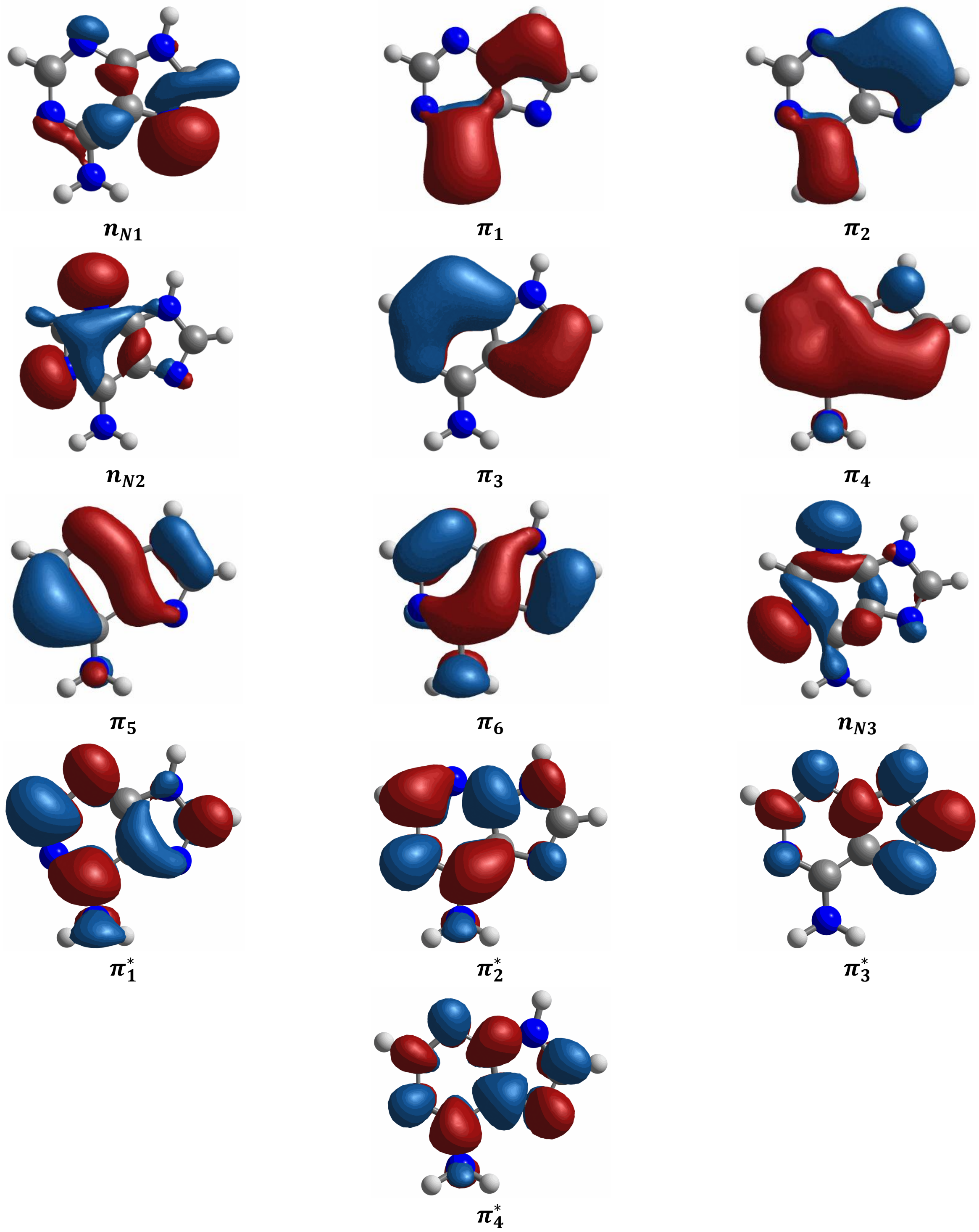

Figure S5: Valence π and $n_{O/N}$ occupied and π unoccupied molecular orbitals of Adenine, together with their labelling. Orbital n_{N1} is removed from almost all active spaces as its occupation number (and therefore its contribution) is negligible.

Table S31: Molecular orbitals included in the active spaces used for the optimization of the conical intersection $(L_a(^1\pi\pi^*)/S_0)_{CI}$ for adenine.

	n_{N1}	π_1	π_2	n_{N2}	π_3	π_4	π_5	π_6	n_{N3}	π_1^*	π_2^*	π_3^*	π_4^*
(18,13)	✓	✓	✓	✓	✓	✓	✓	✓	✓	✓	✓	✓	✓
(16,12)	X	✓	✓	✓	✓	✓	✓	✓	✓	✓	✓	✓	✓
(14,11)	X	X	✓	✓	✓	✓	✓	✓	✓	✓	✓	✓	✓
(12,10)	X	X	X	✓	✓	✓	✓	✓	✓	✓	✓	✓	✓
(10,9)	X	X	X	X	✓	✓	✓	✓	✓	✓	✓	✓	✓
(8,8)	X	X	X	X	X	✓	✓	✓	✓	✓	✓	✓	✓
(8,7)	X	X	X	X	X	✓	✓	✓	✓	✓	✓	✓	X
(6,5)	X	X	X	X	X	X	✓	✓	✓	✓	✓	X	X
(4,3)	X	X	X	X	X	X	X	✓	✓	✓	X	X	X
(2,2)	X	X	X	X	X	X	X	✓	X	✓	X	X	X

Table S32: Occupation numbers for each one of the molecular orbitals involved in the different optimizations of the conical intersection $(L_a(^1\pi\pi^*)/S_0)_{CI}$ of adenine. In green are marked those orbitals that participate in the conical intersection under study.

		nN	π_1	π_2	nN	π_3	π_4	π_5	π_6	nN	π^*	π^*	π^*	π^*
**(2,2)**	State1	X	X	X	X	X	X	X	1.384	X	0.616	X	X	X
	State2	X	X	X	X	X	X	X	1.995	X	0.005	X	X	X
	NIO	X	X	X	X	X	X	X	1.500	X	0.500	X	X	X
**(4,3)**	State1	X	X	X	X	X	X	X	1.369	2.000	0.631	X	X	X
	State2	X	X	X	X	X	X	X	1.993	2.000	0.008	X	X	X
	NIO	X	X	X	X	X	X	X	1.605	1.882	0.513	X	X	X
**(6,5)**	State1	X	X	X	X	X	X	1.938	1.356	1.995	0.644	0.068	X	X
	State2	X	X	X	X	X	X	1.996	1.915	2.000	0.005	0.085	X	X
	NIO	X	X	X	X	X	X	1.990	1.543	1.879	0.499	0.089	X	X
**(8,7)**	State1	X	X	X	X	X	1.999	1.955	1.858	1.955	0.126	0.075	0.049	X
	State2	X	X	X	X	X	1.998	1.948	1.702	1.948	0.285	0.075	0.064	X
	NIO	X	X	X	X	X	1.967	1.952	1.480	1.935	0.519	0.080	0.067	X
**(8,8)**	State1	X	X	X	X	X	1.928	1.957	1.726	1.913	0.261	0.093	0.072	0.050
	State2	X	X	X	X	X	1.955	1.930	1.814	1.938	0.173	0.084	0.062	0.045
	NIO	X	X	X	X	X	1.934	1.929	1.467	1.924	0.522	0.091	0.057	0.075
**(10,9)**	State1	X	X	X	X	1.993	1.903	1.951	1.694	1.924	0.303	0.101	0.077	0.054
	State2	X	X	X	X	1.944	1.918	1.976	1.757	1.932	0.249	0.089	0.080	0.055
	NIO	X	X	X	X	1.945	1.928	1.923	1.471	1.911	0.582	0.090	0.059	0.091
**(12,10)**	State1	X	X	X	1.935	1.963	1.980	1.940	1.902	1.971	0.097	0.098	0.070	0.045
	State2	X	X	X	1.995	1.936	1.920	1.997	1.190	1.898	0.819	0.103	0.078	0.064
	NIO	X	X	X	1.968	1.947	1.944	1.940	1.456	1.924	0.576	0.078	0.063	0.104
**(14,11)**	State1	X	X	1.964	1.927	1.936	1.984	1.992	1.884	1.977	0.117	0.101	0.073	0.047
	State2	X	X	1.991	1.995	1.940	1.923	1.994	1.394	1.904	0.615	0.102	0.078	0.062
	NIO	X	X	1.911	1.969	1.948	1.945	1.943	1.460	1.921	0.577	0.078	0.063	0.107
**(16,12)**	State1	X	1.998	1.996	1.998	1.940	1.923	1.992	1.166	1.901	0.844	0.103	0.078	0.063
	State2	X	1.997	1.963	1.937	1.943	1.980	1.992	1.905	1.972	0.095	0.098	0.072	0.047
	NIO	X	1.994	1.991	1.950	1.969	1.948	1941.000	1.459	1.924	0.578	0.077	0.064	0.106
**(18,13)**	State1	1.998	1.998	1.998	1.996	1.920	1.940	1.991	1.024	1.901	0.987	0.103	0.082	0.063
	State2	1.999	1.997	1.948	1.969	1.963	1.979	1.992	1.903	1.941	0.091	0.099	0.074	0.047
	NIO	1.994	1.986	1.989	1.976	1.952	1.953	1.934	1.458	1.922	0.581	0.086	0.063	0.106

Table S33: Molecular orbitals included in the active spaces used for the optimization of the conical intersection $(L_a(^1\pi\pi^*)/L_b(^1\pi\pi^*))_{CI}$ for adenine.

	n_{N1}	π_1	π_2	n_{N2}	π_3	π_4	π_5	π_6	n_{N3}	π_1^*	π_2^*	π_3^*	π_4^*
(16,12)	X	✓	✓	✓	✓	✓	✓	✓	✓	✓	✓	✓	✓
(14,11)	X	X	✓	✓	✓	✓	✓	✓	✓	✓	✓	✓	✓
(12,10)	X	X	X	✓	✓	✓	✓	✓	✓	✓	✓	✓	✓
(12,9)	X	X	X	✓	✓	✓	✓	✓	✓	✓	✓	✓	X
(10,8)	X	X	X	✓	X	✓	✓	✓	✓	✓	✓	✓	X
(8,7)	X	X	X	X	X	✓	✓	✓	✓	✓	✓	✓	X
(8,6)	X	X	X	X	X	✓	✓	✓	✓	✓	✓	X	X
(6,5)	X	X	X	X	X	X	✓	✓	✓	✓	✓	X	X
(4,4)	X	X	X	X	X	X	✓	✓	X	✓	✓	X	X
(4,3)	X	X	X	X	X	X	✓	✓	X	✓	X	X	X

Table S34: Occupation numbers for each one of the molecular orbitals involved in the different optimizations of the conical intersection $(L_a(^1\pi\pi^*)/L_b(^1\pi\pi^*))_{CI}$ of adenine. In green are marked those orbitals that participate in the conical intersection under study.

		nN	π_1	π_2	nN	π_3	π_4	π_5	π_6	nN	π*	π*	π*	π*
**(4,3)**	State1	X	X	X	X	X	X	1.609	2.000	X	0.391	X	X	X
	State2	X	X	X	X	X	X	2.000	1.515	X	0.486	X	X	X
	NIO	X	X	X	X	X	X	1.510	1.670	X	0.821	X	X	X
**(4,4)**	State1	X	X	X	X	X	X	1.252	1.782	X	0.771	0.195	X	X
	State2	X	X	X	X	X	X	1.921	1.451	X	0.552	0.076	X	X
	NIO	X	X	X	X	X	X	1.509	1.423	X	0.898	0.170	X	X
**(6,5)**	State1	X	X	X	X	X	X	1.220	1.750	1.992	0.846	0.192	X	X
	State2	X	X	X	X	X	X	1.337	1.943	1.976	0.683	0.061	X	X
	NIO	X	X	X	X	X	X	1.486	1.527	1.873	0.952	0.162	X	X
**(8,6)**	State1	X	X	X	X	X	1.992	1.900	1.149	1.980	0.867	0.112	X	X
	State2	X	X	X	X	X	1.998	1.333	1.962	1.816	0.707	0.184	X	X
	NIO	X	X	X	X	X	1.971	1.648	1.447	1.770	0.954	0.209	X	X
**(8,7)**	State1	X	X	X	X	X	1.911	1.650	1.274	1.989	0.773	0.322	0.082	X
	State2	X	X	X	X	X	1.971	1.349	1.930	1.886	0.684	0.117	0.062	X
	NIO	X	X	X	X	X	1.899	1.717	1.377	1.783	0.892	0.256	0.077	X
**(10,8)**	State1	X	X	X	1.922	X	1.990	1.867	1.452	1.974	0.580	0.141	0.074	X
	State2	X	X	X	1.995	X	1.976	1.278	1.868	1.924	0.770	0.125	0.064	X
	NIO	X	X	X	1.955	X	1.937	1.517	1.514	1.936	0.914	0.153	0.075	X
**(12,9)**	State1	X	X	X	1.924	1.997	1.988	1.869	1.461	1.974	0.571	0.141	0.075	X
	State2	X	X	X	1.977	1.997	1.923	1.267	1.864	1.995	0.782	0.129	0.067	X
	NIO	X	X	X	1.957	1.994	1.937	1.517	1.516	1.937	0.911	0.154	0.076	X
**(12,10)**	State1	X	X	X	1.925	1.935	1.970	1.886	1.501	1.973	0.525	0.139	0.082	0.067
	State2	X	X	X	1.992	1.940	1.912	1.169	1.816	1.975	0.900	0.153	0.080	0.063
	NIO	X	X	X	1.938	1.953	1.933	1.519	1.493	1.932	0.915	0.067	0.089	0.162
**(14,11)**	State1	X	X	1.922	1.992	1.934	1.986	1.858	1.454	1.973	0.576	0.152	0.085	0.067
	State2	X	X	1.976	1.990	1.940	1.910	1.242	1.844	1.995	0.815	0.139	0.084	0.064
	NIO	X	X	1.989	1.959	1.939	1.935	1.520	1.493	1.933	0.912	0.068	0.089	0.162
**(16,12)**	State1	X	1.995	1.923	1.992	1.935	1.988	1.851	1.437	1.976	0.597	0.155	0.084	0.068
	State2	X	1.997	1.995	1.990	1.942	1.914	1.265	1.857	1.977	0.786	0.131	0.082	0.065
	NIO	X	1.993	1.989	1.957	1.939	1.939	1.519	1.498	1.936	0.914	0.069	0.088	0.159

Table S35: Molecular orbitals included in the active spaces used for the optimization of the conical intersection $(L_b(^1\pi\pi^*)/^1n_N\pi^*)_{CI}$ for adenine.

	n_{N1}	π_1	π_2	n_{N2}	π_3	π_4	π_5	π_6	n_{N3}	π_1^*	π_2^*	π_3^*	π_4^*
(16,12)	X	✓	✓	✓	✓	✓	✓	✓	✓	✓	✓	✓	✓
(14,11)	X	X	✓	✓	✓	✓	✓	✓	✓	✓	✓	✓	✓
(12,10)	X	X	X	✓	✓	✓	✓	✓	✓	✓	✓	✓	✓
(10,9)	X	X	X	✓	✓	X	✓	✓	✓	✓	✓	✓	✓
(10,8)	X	X	X	✓	✓	X	✓	✓	✓	✓	✓	✓	X
(6,6)	X	X	X	X	X	X	✓	✓	✓	✓	✓	✓	X
(6,5)	X	X	X	X	X	X	✓	✓	✓	✓	✓	X	X

Table S36: Occupation numbers for each one of the molecular orbitals involved in the different optimizations of the conical intersection $(L_b(^1\pi\pi^*)/^1n_N\pi^*)_{CI}$ of adenine. Those orbitals that participate in the conical intersection have their cell in green.

		nN	π_1	π_2	nN	π_3	π_4	π_5	π_6	nN	π^*	π^*	π^*	π^*
**(6,5)**	State1	X	X	X	X	X	X	1.978	1.925	0.942	1.068	0.086	X	X
	State2	X	X	X	X	X	X	1.261	1.470	1.995	0.764	0.509	X	X
	NIO	X	X	X	X	X	X	1.475	1.621	1.683	0.883	0.338	X	X
**(6,6)**	State1	X	X	X	X	X	X	1.956	1.849	1.078	0.951	0.134	0.031	X
	State2	X	X	X	X	X	X	1.709	1.941	1.187	0.887	0.240	0.036	X
	NIO	X	X	X	X	X	X	1.483	1.701	1.641	0.833	0.306	0.035	X
**(10,8)**	State1	X	X	X	1.907	1.965	X	1.927	1.895	1.105	0.981	0.159	0.062	X
	State2	X	X	X	1.997	1.875	X	1.357	1.524	1.992	0.674	0.473	0.109	X
	NIO	X	X	X	1.877	1.918	X	1.551	1.652	1.747	0.826	0.336	0.095	X
**(10,9)**	State1	X	X	X	1.981	1.884	X	1.319	1.560	1.995	0.714	0.433	0.099	0.016
	State2	X	X	X	1.900	1.966	X	1.932	1.919	1.095	0.969	0.140	0.062	0.016
	NIO	X	X	X	1.844	1.922	X	1.524	1.640	1.812	0.836	0.319	0.016	0.087
**(12,10)**	State1	X	X	X	1.930	1.890	1.981	1.297	1.614	1.956	0.773	0.378	0.111	0.070
	State2	X	X	X	1.939	1.928	1.954	1.904	1.776	1.199	0.901	0.238	0.095	0.065
	NIO	X	X	X	1.920	1.893	1.929	1.549	1.658	1.721	0.829	0.328	0.069	0.104
**(14,11)**	State1	X	X	1.989	1.930	1.903	1.972	1.944	1.683	1.255	0.828	0.320	0.106	0.071
	State2	X	X	1.989	1.941	1.905	1.963	1.930	1.711	1.244	0.852	0.294	0.102	0.069
	NIO	X	X	1.882	1.923	1.988	1.930	1.550	1.640	1.756	0.832	0.323	0.071	0.105
**(16,12)**	State1	X	1.996	1.988	1.999	1.929	1.879	1.361	1.516	1.998	0.673	0.473	0.075	0.114
	State2	X	1.999	1.989	1.913	1.949	1.967	1.923	1.899	1.100	0.980	0.147	0.059	0.077
	NIO	X	1.996	1.914	1.924	1.988	1.933	1.548	1.643	1.724	0.832	0.324	0.072	0.102

Finally, for the adenine $(L_a(^1\pi\pi^*)/S_0)_{CI}$ conical intersection we found similar results to those for the cytosine $(^1\pi\pi^*/S_0)_{CI}$ (Table S2).Larger active spaces, have one of the electrons in Homo (occ ~2) that goes to the Lumo in which the occupancies change to ~1 for both molecular orbitals, with a difference situation as the active space is reduced. However, these changes do not explain the differences observed in the topography of the different optimized intersections. The same conclusions are drawn for the last two conical intersections studied, as the small changes observed in the occupations are not related to the quadrant changes of

the structures.

$\mathcal{P}$ vs $\mathcal{B}$ results using a triple-ζ basis set

To complete the results of this work, all the optimizations performed with a double-ζ basis set, were performed using a triple-ζ basis set to see how this factor affects the topography of the conical intersections. The following figures are organized in the same manner as the Dζ ones in the main text.

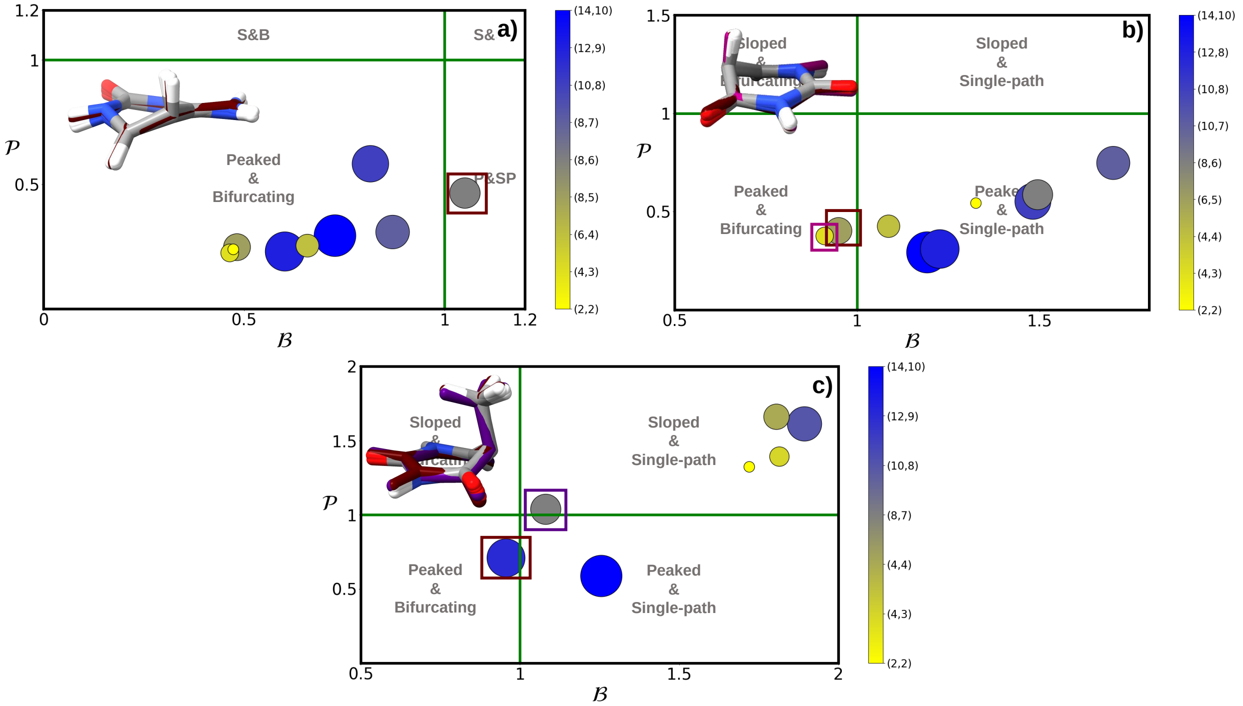

Figure S6: $\mathcal{P}$ and $\mathcal{B}$ parameters of $(^1\pi\pi^*/S_0)_{CI}$ using multiple different active spaces and triple-ζ basis set (see Computational Details) for a) cytosine, b) uracil and c) thymine. Active space size is denoted by both marker size and the contour gradient colour provided in the right hand side of each panel. A picture with the superimposed geometries of all optimised conical intersections are provided as in-sets, with the coloured structures representing the outlier intersections marked with a square.

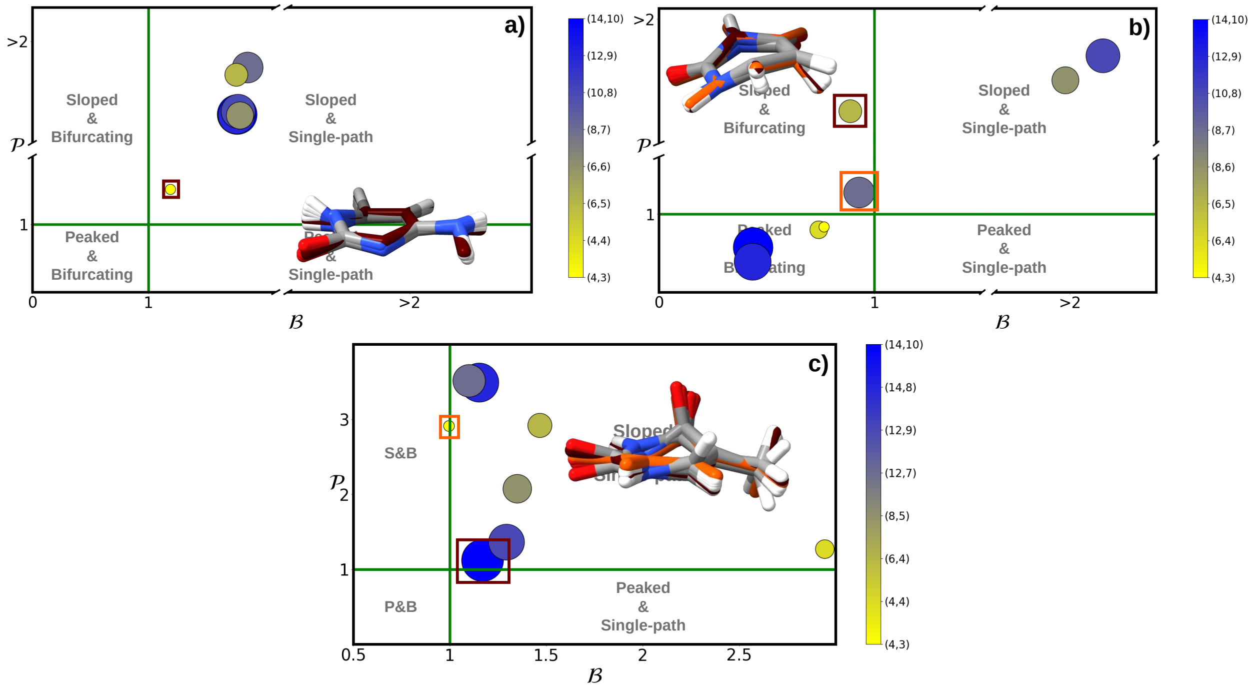

Figure S7: $\mathcal{P}$ and $\mathcal{B}$ parameters of $(^1n_O\pi^*/^1\pi\pi^*)_{CI}$ using multiple different active spaces and triple-ζ basis set (see Computational Details) for a) cytosine, b) uracil and c) thymine. Active space size is denoted by both marker size and the contour gradient colour provided in the right hand side of each panel. A picture with the superimposed geometries of all optimised conical intersections are provided as in-sets, with the coloured structures representing the outlier intersections marked with a square.

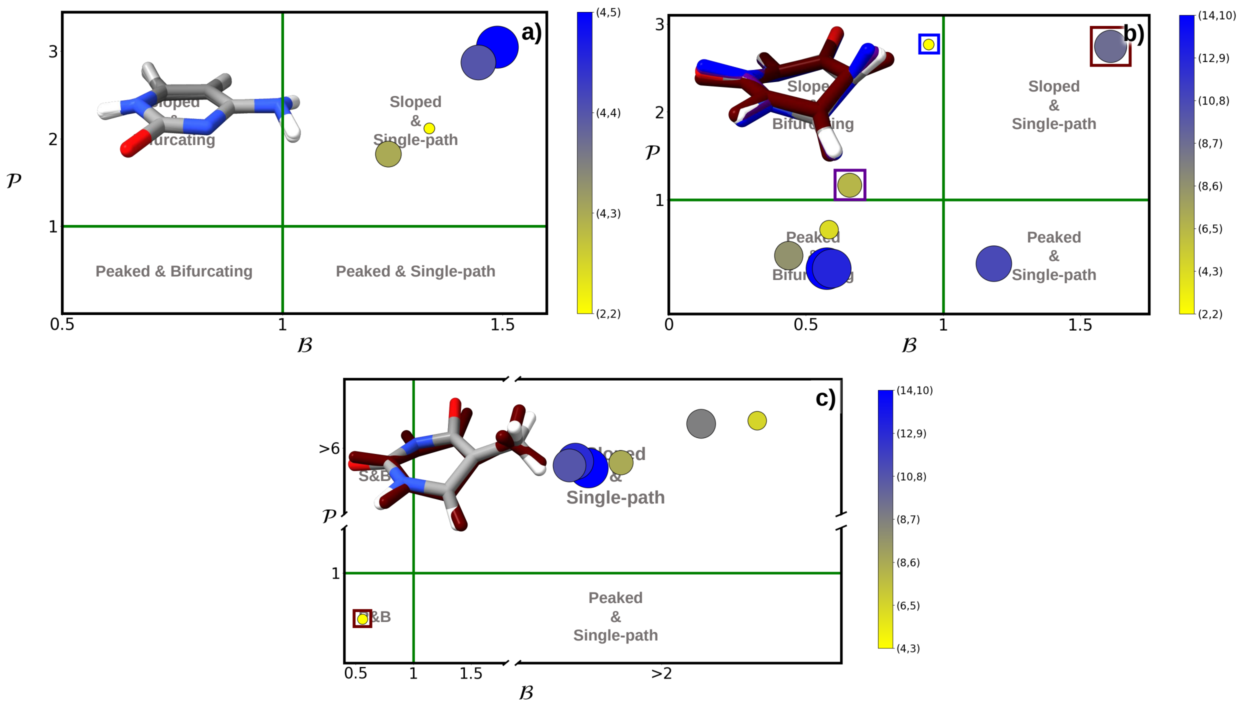

Figure S8: $\mathcal{P}$ and $\mathcal{B}$ parameters of the $(^1n_O\pi^*/S_0)_{CI}$ using multiple different active spaces and triple-ζ basis set (see Computational Details) for a) cytosine, b) uracil and c) thymine. Active space size is denoted by both marker size and the contour gradient colour provided in the right hand side of each panel. A picture with the superimposed geometries of all optimised conical intersections are provided as in-sets, with the coloured structures representing the outlier intersections marked with a square.

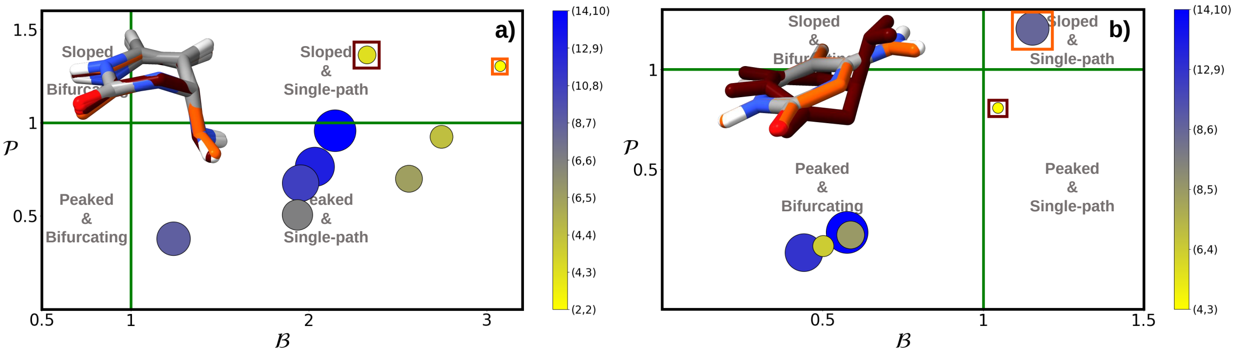

Figure S9: $\mathcal{P}$ and $\mathcal{B}$ parameters of the a) $(^1n_N\pi^*/S_0)_{CI}$ and b) $(^1n_N\pi^*/^1\pi\pi^*)_{CI}$ of cytosine with a triple-ζ basis set. Active space size is denoted by both marker size and the contour gradient colour provided in the right hand side of each panel. A picture with the superimposed geometries of all optimised conical intersections are provided as in-sets, with the coloured structures representing the outlier intersections marked with a square.

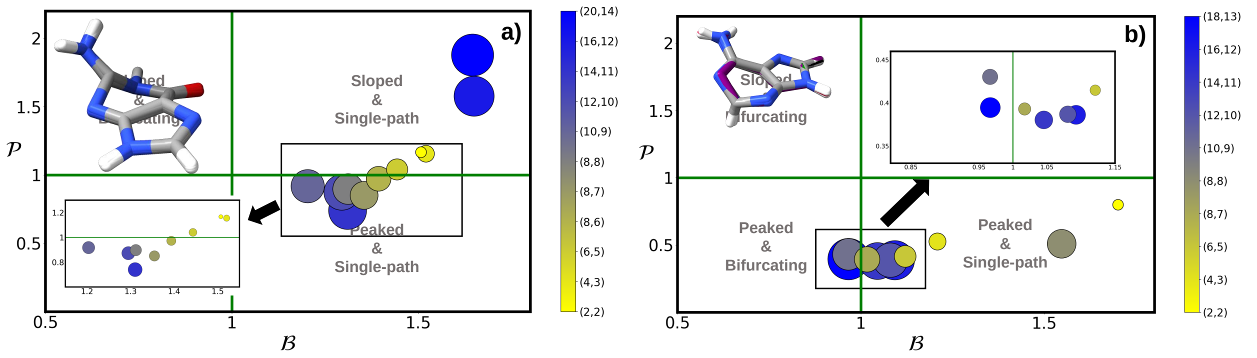

Figure S10: $\mathcal{P}$ and $\mathcal{B}$ parameters of $(L_a(^1\pi\pi^*)/S_0)_{CI}$ for a) guanine and b) adenine using multiple different active spaces and triple-ζ basis set (see Computational Details). Active space size is denoted by both marker size and the contour gradient colour provided in the right hand side of each panel. A picture with the superimposed geometries of all optimised conical intersections are provided as in-sets, with the coloured structures representing the outlier intersections marked with a square.

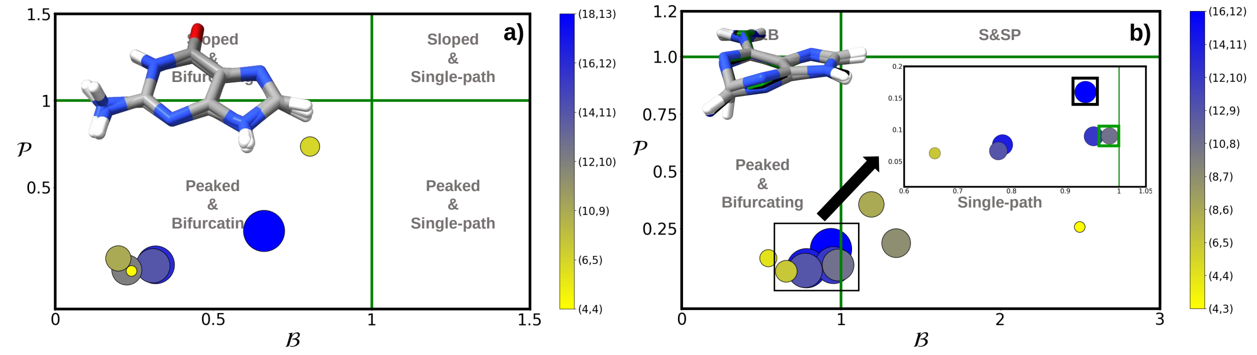

Figure S11: $\mathcal{P}$ and $\mathcal{B}$ parameters of $(L_a(^1\pi\pi^*)/L_b(^1\pi\pi^*))_{CI}$ using multiple different active spaces and triple-ζ basis set (see Computational Details) for guanine (a) and adenine (b). Active space size is denoted by both marker size and the contour gradient colour provided in the right hand side of each panel. A picture with the superimposed geometries of all optimised conical intersections are provided as in-sets.

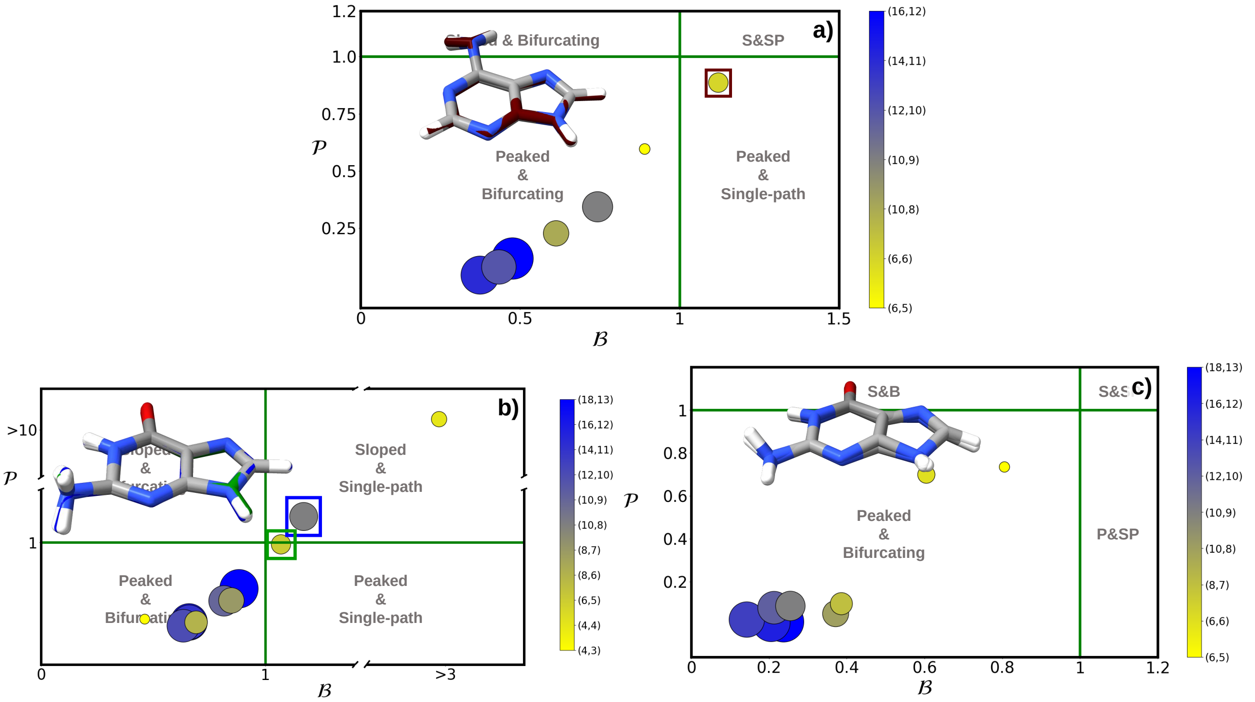

Figure S12: $\mathcal{P}$ and $\mathcal{B}$ parameters of $(L_b(^1\pi\pi^*)/^1n_N\pi^*)_{CI}$ (a) for adenine and $(L_a(^1\pi\pi^*)/^1n_O\pi^*)_{CI}$ (b) and $(L_b(^1\pi\pi^*)/^1n_N\pi^*)_{CI}$ (c) for guanine using multiple different active spaces and triple-ζ basis set (see Computational Details). Active space size is denoted by both marker size and the contour gradient colour provided in the right hand side of each panel. A picture with the superimposed geometries of all optimised conical intersections are provided as in-sets, with the coloured structures representing the outlier intersection marked with a square.

Root Mean Squared Deviation analyses

The final section of the supplementary information features a collection of figures displaying the root mean square deviations (RMSD) between the optimized geometries of each active space. For all conical intersections, the reference point is the optimized structure with the largest active space. The figures also provide information on the dihedral angle that exhibits the most significant change among the various structures (inset), which best correlates with the RMSD values.

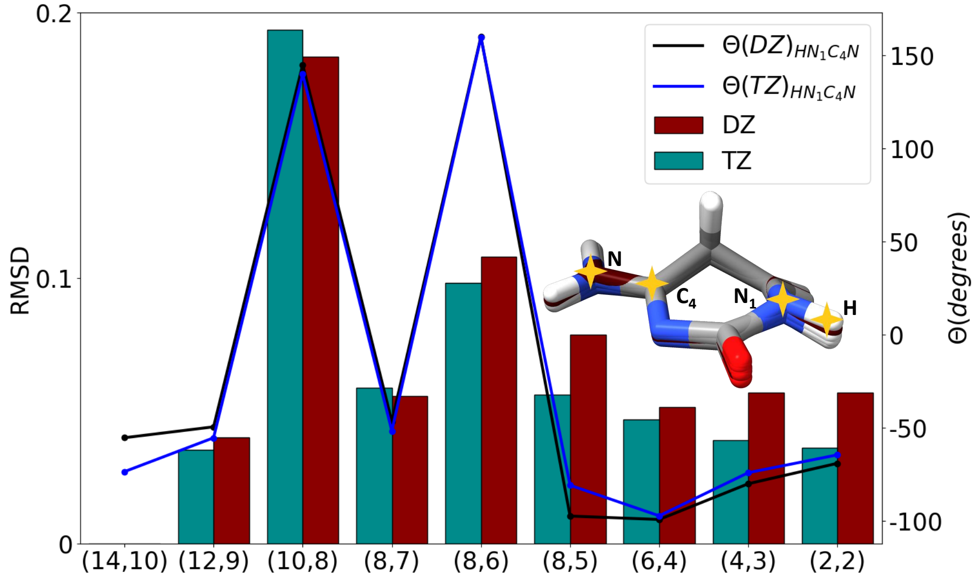

Figure S13: Root Mean Squared Deviation (RMSD) and dihedral angle variation between H-N_1-C_4-N atoms for the different active spaces used in the optimization of the conical intersection $(^1\pi\pi^*/S_0)_{CI}$ of cytosine. Yellow symbols highlight atoms in the dihedral angle in the superimposed geometries (Inset).

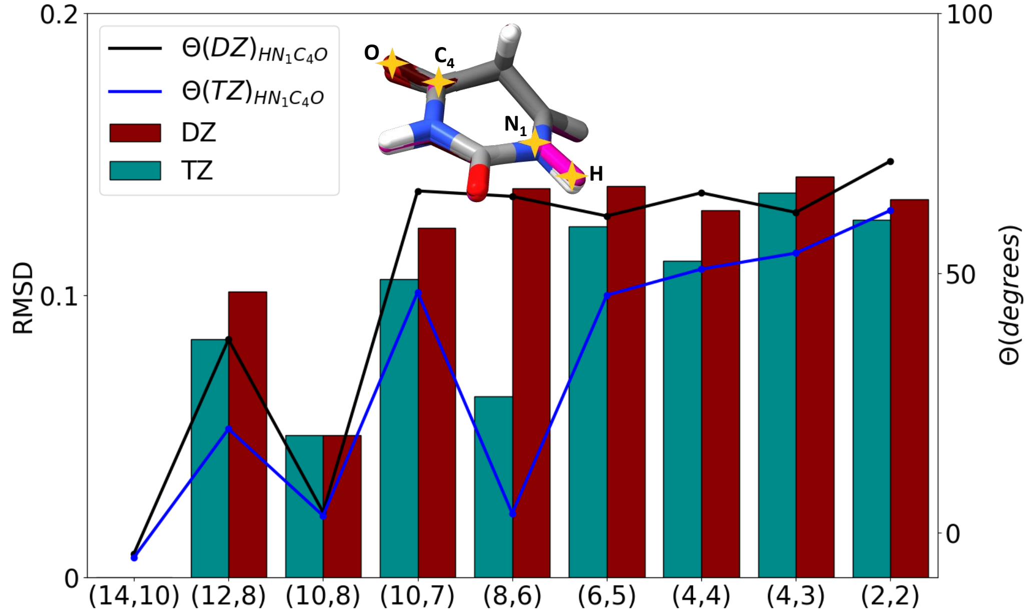

Figure S14: Root Mean Squared Deviation (RMSD) and dihedral angle variation between H-N_1-C_4-O atoms for the different active spaces used in the optimization of the conical intersection $(^1\pi\pi^*/S_0)_{CI}$ of uracil. Yellow symbols highlight atoms in the dihedral angle in the superimposed geometries (Inset).

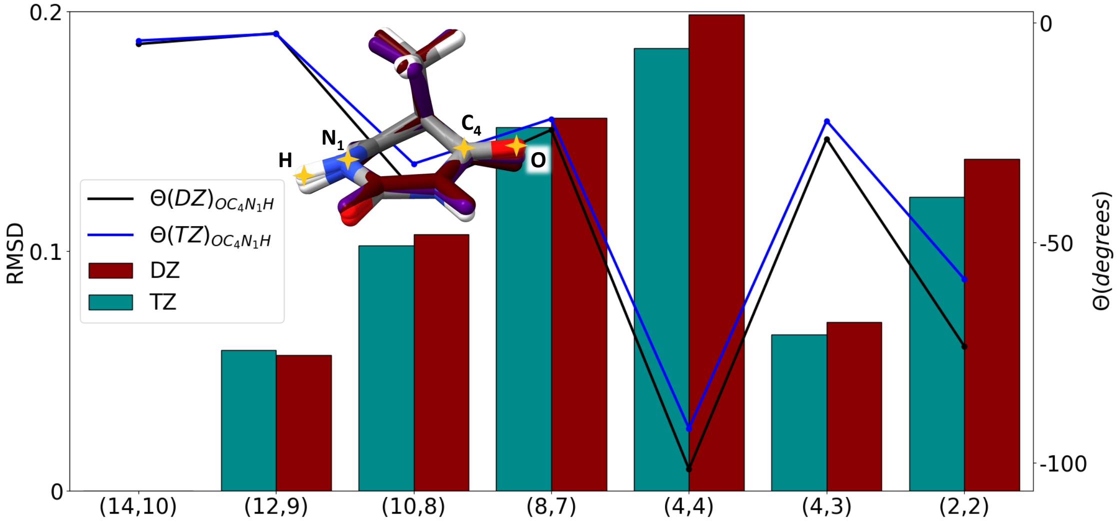

Figure S15: Root Mean Squared Deviation (RMSD) and dihedral angle variation between O-C_4-N_1-H atoms for the different active spaces used in the optimization of the conical intersection $(^1\pi\pi^*/S_0)_{CI}$ of thymine. Yellow symbols highlight atoms in the dihedral angle in the superimposed geometries (Inset).

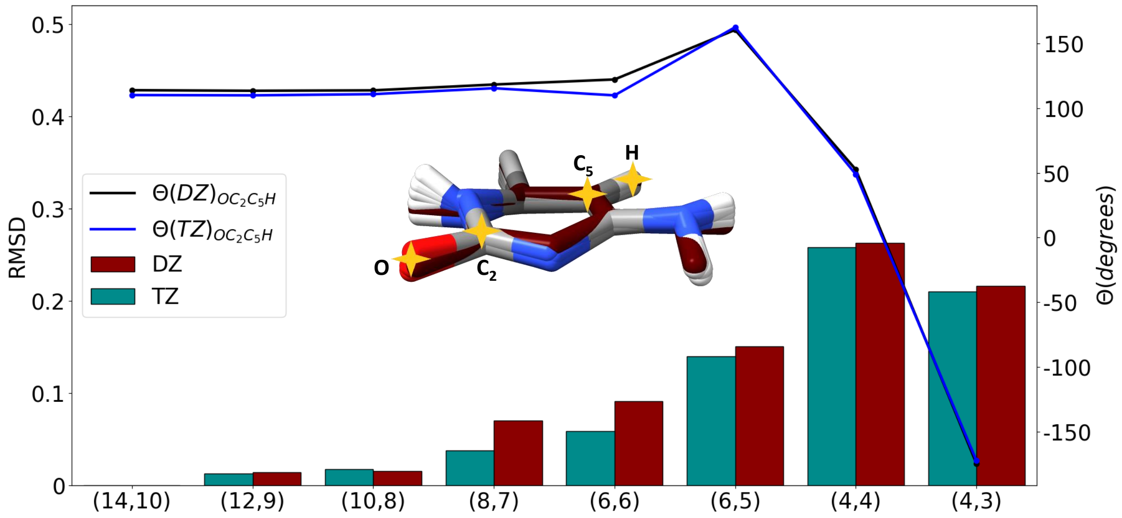

Figure S16: Root Mean Squared Deviation (RMSD) and dihedral angle variation between O-C_2-C_5-H atoms for the different active spaces used in the optimization of the conical intersection $({}^1n_O\pi^*/{}^1\pi\pi^*)_{CI}$ of cytosine. Yellow symbols highlight atoms in the dihedral angle in the superimposed geometries (Inset).

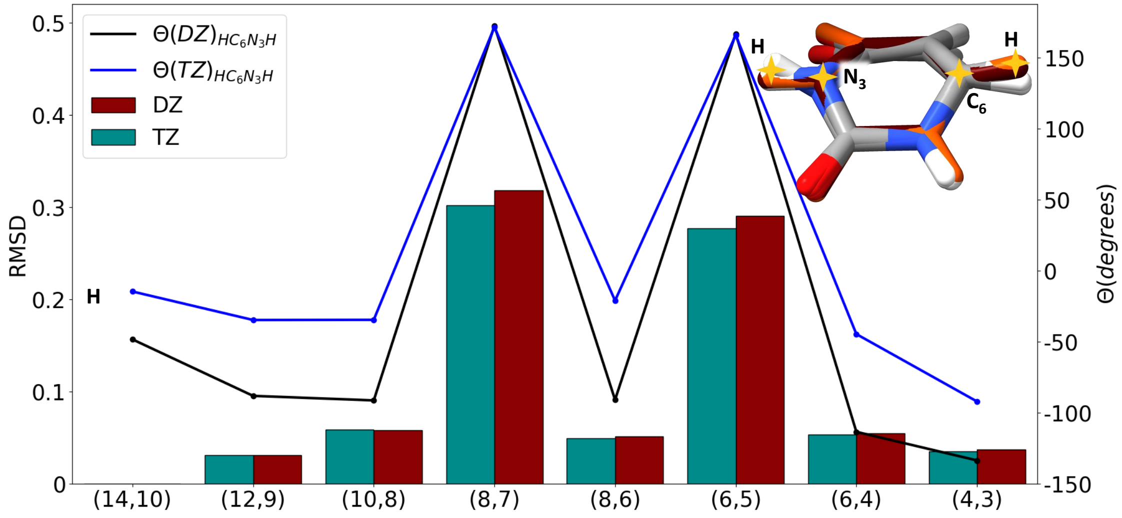

Figure S17: Root Mean Squared Deviation (RMSD) and dihedral angle variation between H-C_6-N_3-H atoms for the different active spaces used in the optimization of the conical intersection $({}^1n_O\pi^*/{}^1\pi\pi^*)_{CI}$ of uracil. Yellow symbols highlight atoms in the dihedral angle in the superimposed geometries (Inset).

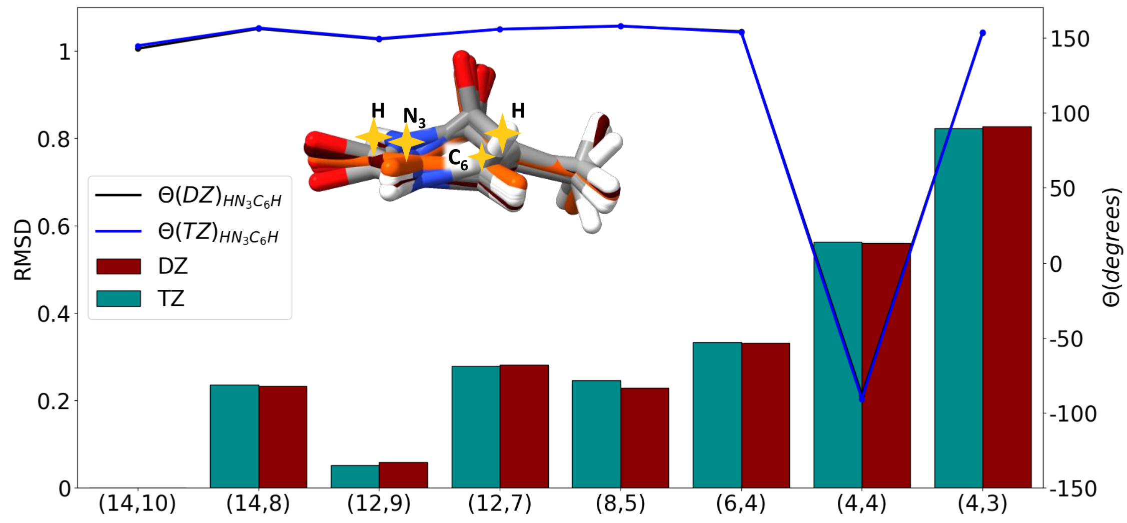

Figure S18: Root Mean Squared Deviation (RMSD) and dihedral angle variation between H-N_3-C_6-H atoms for the different active spaces used in the optimization of the conical intersection $(^1n_O\pi^*/^1\pi\pi^*)_{CI}$ of thymine. Yellow symbols highlight atoms in the dihedral angle in the superimposed geometries (Inset).

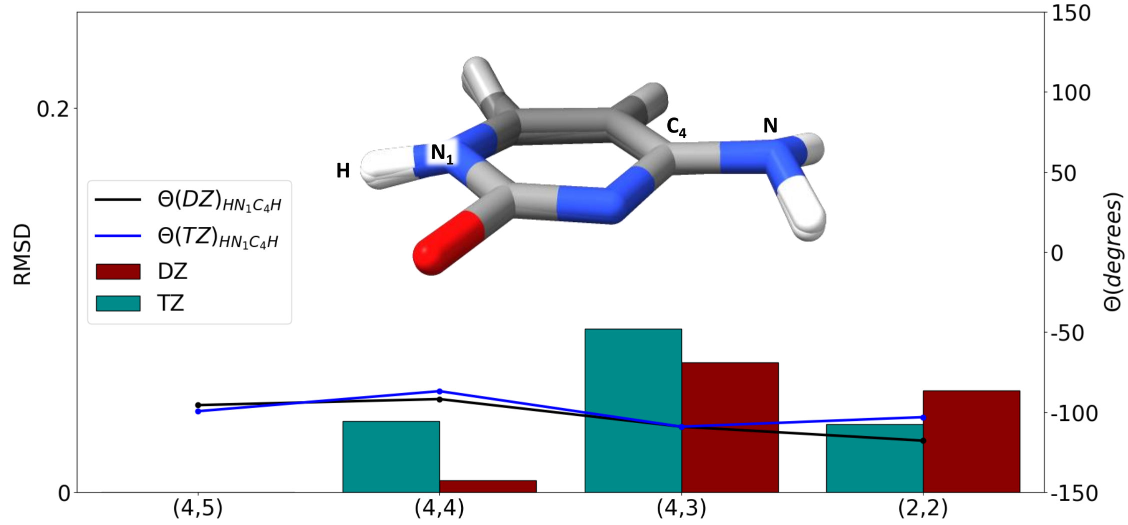

Figure S19: Root Mean Squared Deviation (RMSD) and dihedral angle variation between H-N_1-C_4-N atoms for the different active spaces used in the optimization of the conical intersection $(^1n_O\pi^*/S_0)_{CI}$ of cytosine. Yellow symbols highlight atoms in the dihedral angle in the superimposed geometries (Inset).

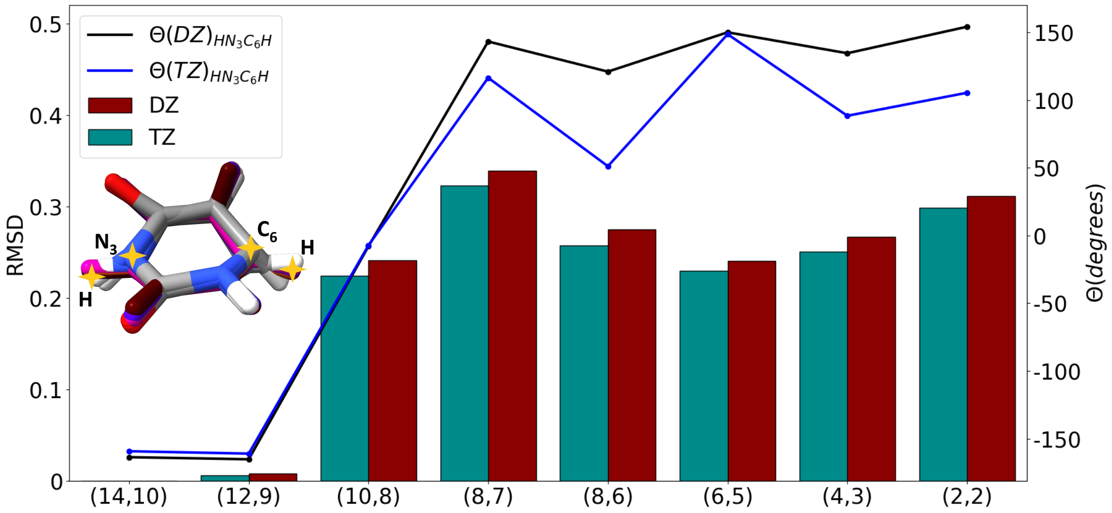

Figure S20: Root Mean Squared Deviation (RMSD) and dihedral angle variation between H-N_3-C_6-H atoms for the different active spaces used in the optimization of the conical intersection $(^1n_O\pi^*/S_0)_{CI}$ of uracil. Yellow symbols highlight atoms in the dihedral angle in the superimposed geometries (Inset).

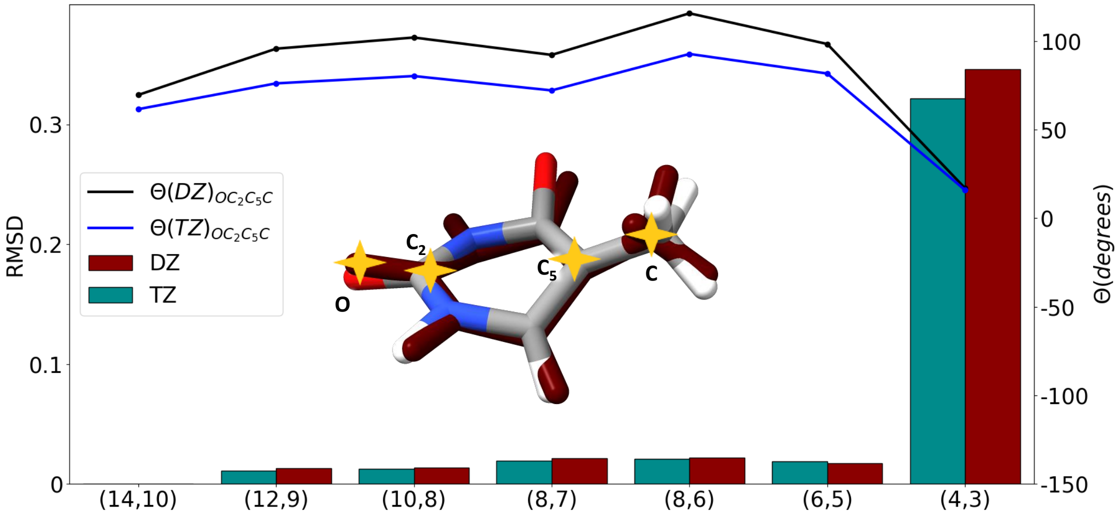

Figure S21: Root Mean Squared Deviation (RMSD) and dihedral angle variation between O-C_2-C_5-C atoms for the different active spaces used in the optimization of the conical intersection $(^1n_O\pi^*/S_0)_{CI}$ of thymine. Yellow symbols highlight atoms in the dihedral angle in the superimposed geometries (Inset).

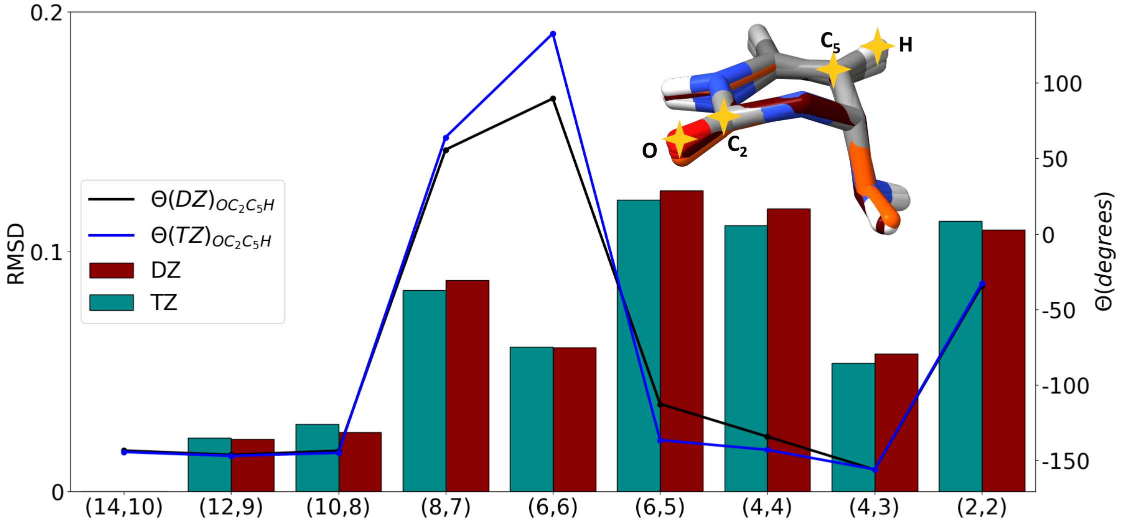

Figure S22: Root Mean Squared Deviation (RMSD) and dihedral angle variation between O-C_2-C_5-H atoms for the different active spaces used in the optimization of the conical intersection $(^1n_N\pi^*/S_0)_{CI}$ of cytosine. Yellow symbols highlight atoms in the dihedral angle in the superimposed geometries (Inset).

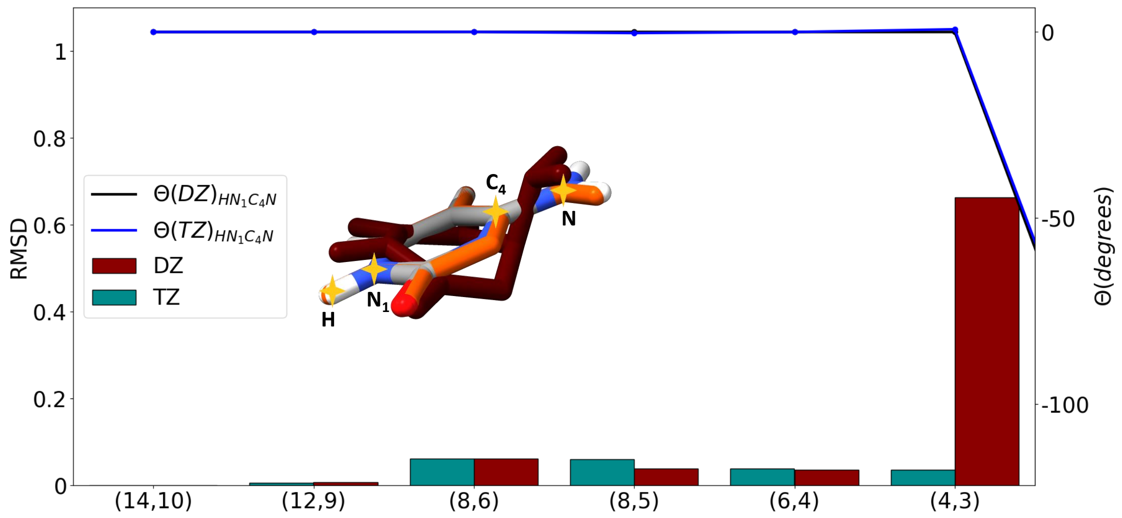

Figure S23: Root Mean Squared Deviation (RMSD) and dihedral angle variation between H-N_1-C_4-N atoms for the different active spaces used in the optimization of the conical intersection $(^1n_N\pi^*/^1\pi\pi^*)_{CI}$ of cytosine. Yellow symbols highlight atoms in the dihedral angle in the superimposed geometries (Inset).

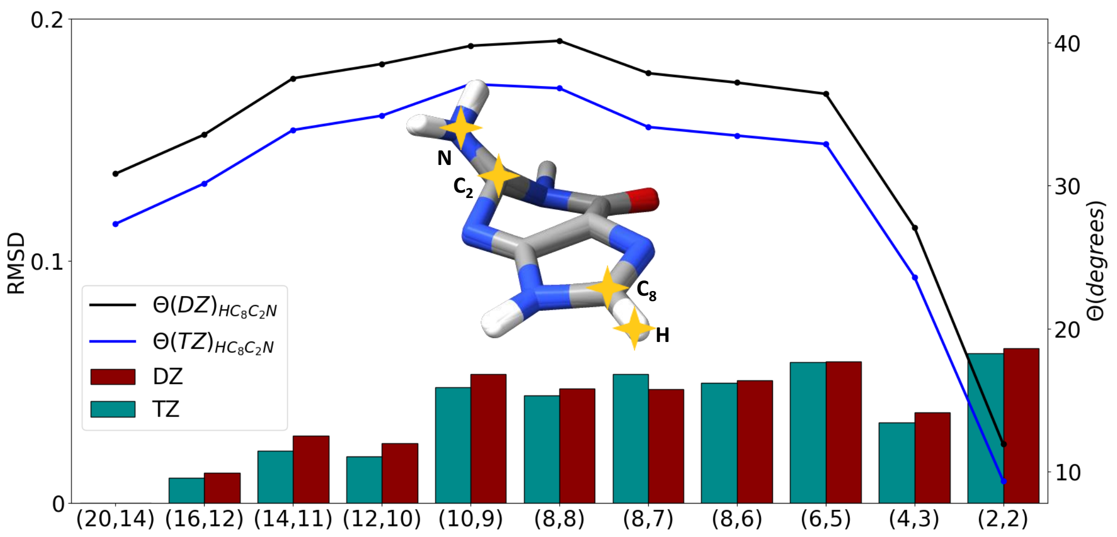

Figure S24: Root Mean Squared Deviation (RMSD) and dihedral angle variation between H-C_8-C_2-N atoms for the different active spaces used in the optimization of the conical intersection $(L_a(^1\pi\pi^*)/S_0)_{CI}$ of guanine. Yellow symbols highlight atoms in the dihedral angle in the superimposed geometries (Inset).

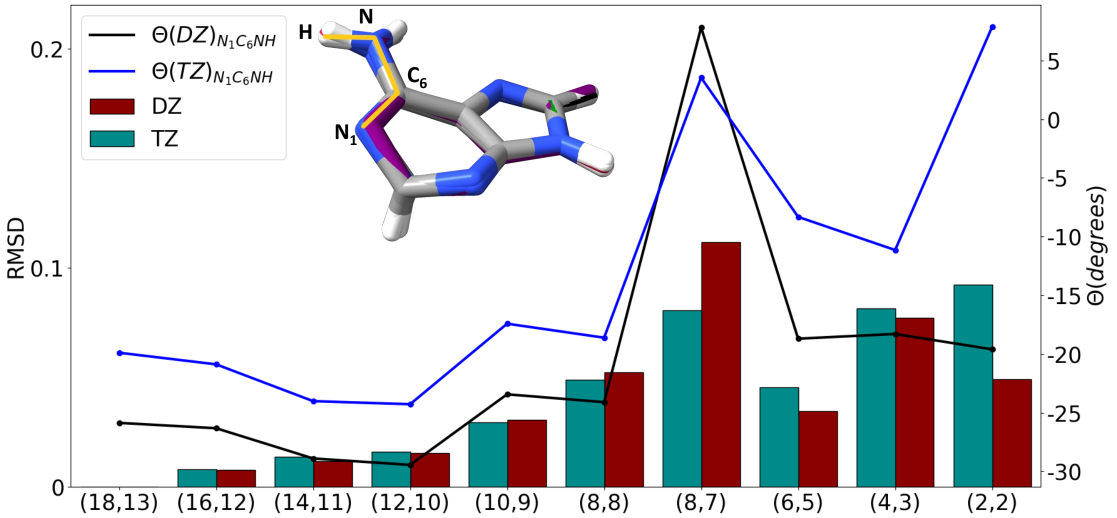

Figure S25: Root Mean Squared Deviation (RMSD) and dihedral angle variation between N_1-C_6-N-H atoms for the different active spaces used in the optimization of the conical intersection $(L_a(^1\pi\pi^*)/S_0)_{CI}$ of adenine. Yellow line highlights atoms in the dihedral angle in the superimposed geometries (Inset).

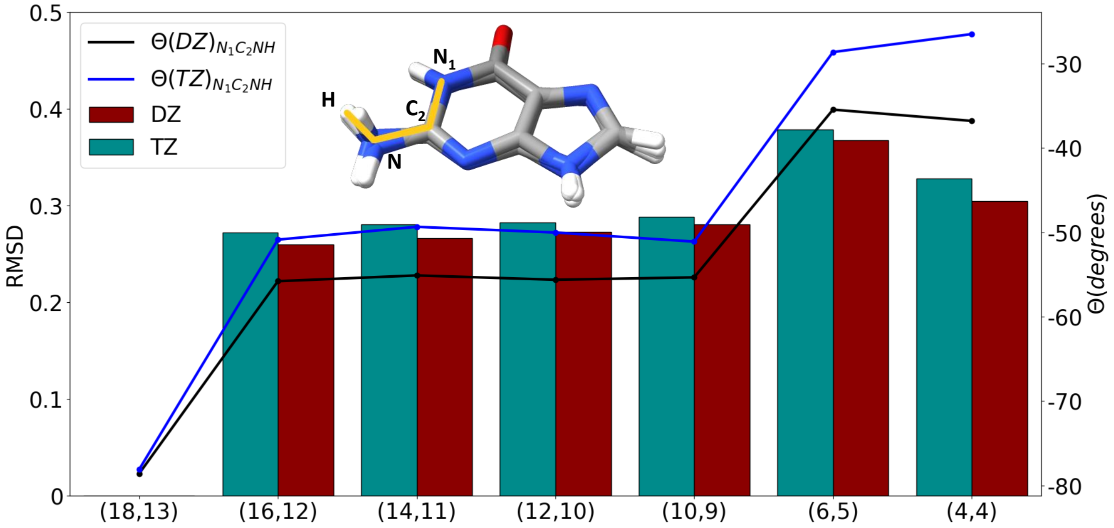

Figure S26: Root Mean Squared Deviation (RMSD) and dihedral angle variation between N_1-C_2-N-H atoms for the different active spaces used in the optimization of the conical intersection $(L_a(^1\pi\pi^*)/L_b(^1\pi\pi^*))_{CI}$ of guanine. Yellow line highlights atoms in the dihedral angle in the superimposed geometries (Inset).

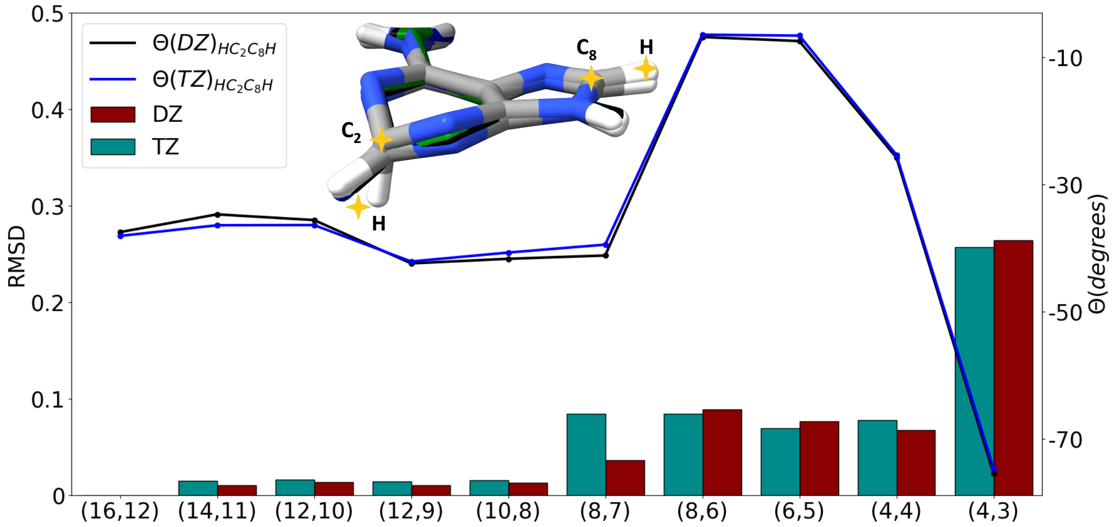

Figure S27: Root Mean Squared Deviation (RMSD) and dihedral angle variation between H-C_2-C_8-H atoms for the different active spaces used in the optimization of the conical intersection $(L_a(^1\pi\pi^*)/L_b(^1\pi\pi^*))_{CI}$ of adenine. Yellow symbols highlight atoms in the dihedral angle in the superimposed geometries (Inset).

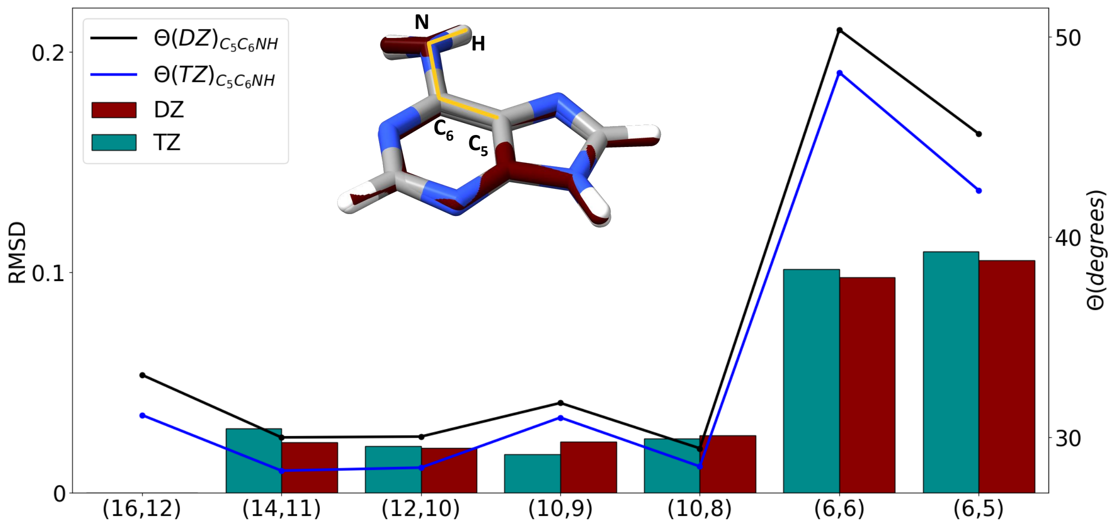

Figure S28: Root Mean Squared Deviation (RMSD) and dihedral angle variation between C_5-C_6-N-H atoms for the different active spaces used in the optimization of the conical intersection $(L_b(^1\pi\pi^*)/^1n_N\pi^*)_{CI}$ of adenine. Yellow line highlights atoms in the dihedral angle in the superimposed geometries (Inset).

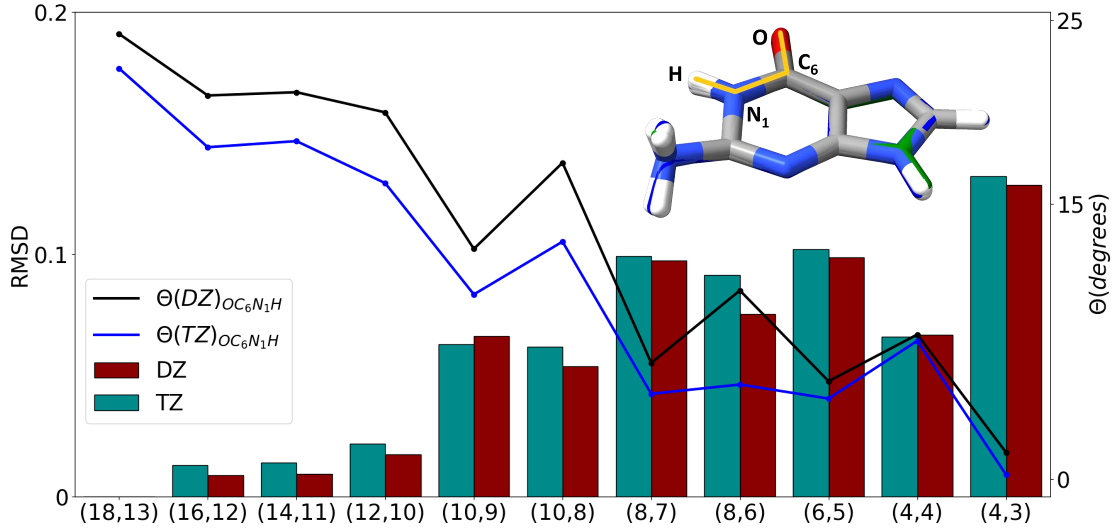

Figure S29: Root Mean Squared Deviation (RMSD) and dihedral angle variation between O-C_6-N_1-H atoms for the different active spaces used in the optimization of the conical intersection $(L_a(^1\pi\pi^*)/^1n_O\pi^*)_{CI}$ of guanine. Yellow line highlights atoms in the dihedral angle in the superimposed geometries (Inset).

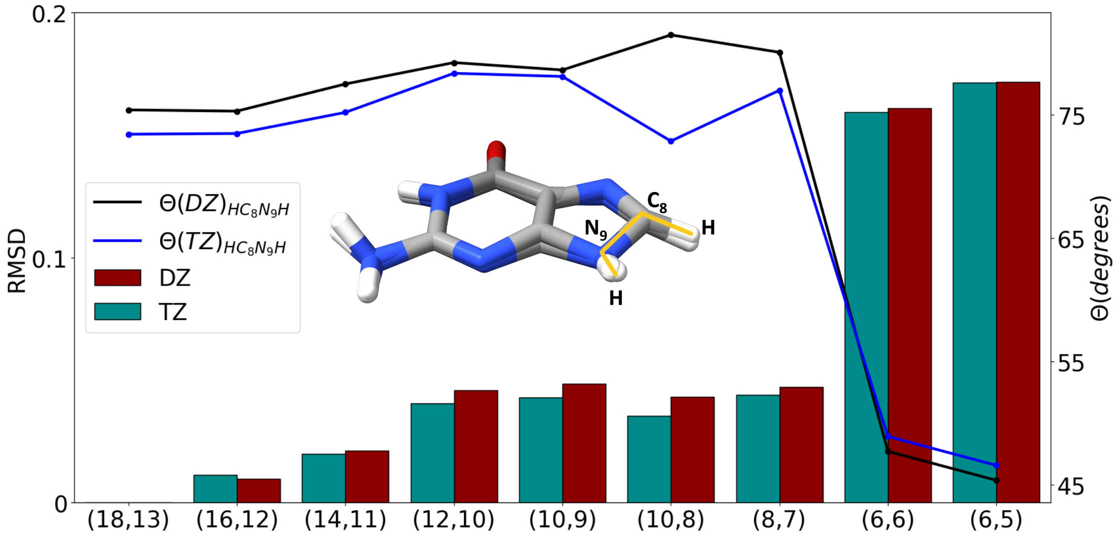

Figure S30: Root Mean Squared Deviation (RMSD) and dihedral angle variation between H-C_8-N_9-H atoms for the different active spaces used in the optimization of the conical intersection $(L_b(^1\pi\pi^*)/^1n_N\pi^*)_{CI}$ of guanine. Yellow line highlights atoms in the dihedral angle in the superimposed geometries (Inset).
